# Decline of *Tephroseris helenitis* in Hessia (Germany) over the last 120 years: Modeling implies the gradual disappearance of its temperature niche for flower induction and germination

**DOI:** 10.1002/ece3.10769

**Published:** 2023-12-06

**Authors:** Eva Maria Griebeler, Joachim W. Kadereit

**Affiliations:** ^1^ Institut für Organismische und Molekulare Evolutionsbiologie Johannes Gutenberg‐Universität Mainz Mainz Germany; ^2^ Present address: Systematik, Biodiversität und Evolution der Pflanzen Ludwig‐Maximilians‐Universität München Munich Germany

**Keywords:** climate change, devernalization, germination, niche broadening, niche modeling, niche shift, vernalization

## Abstract

*Tephroseris helenitis* is a perennial herb that experienced a severe decline of species records over the last 120 years in the state of Hessia, Germany. Here, the species is found in humid habitats with moderate temperatures. In this modeling study, we assessed changes in climatic conditions between the periods 1900–1949, 1950–1979, 1980–1999 and 2000–2020 and explored whether these changes can explain the decline of records of *T. helenitis*. Climatic variables used were monthly precipitation sums, monthly mean, minimum and maximum temperatures, monthly temperature ranges as well as annual precipitation sum and annual mean temperature. For the majority of these variables, changes were significant across periods. Minimum temperatures in March, April and July (Tmin_Mar, Tmin_Apr, Tmin_Jul) best explained species presences and absences in 1900–1949 and 1950–1979. The species shifted its realized niche towards lower Tmin_Mar and narrowed its niche on Tmin_Apr and Tmin_Jul between these two periods. March, April and July are crucial in the life cycle of *T. helenitis*. Tmin_Mar and Tmin_Apr are related to the induction of flowering through a period of low temperatures (vernalization), and Tmin_Jul is related to seed germination. Documented increasing March and April temperatures as well as autumn and winter temperatures in the past 120 years may imply that vernalization became increasingly unsuccessful for the species and increasing July temperatures may have decreased its germination success. Given the disappearance of its temperature niche (Tmin_Mar, Tmin_Apr, Tmin_Jul) due to ongoing global warming not only in Hessia and Germany, we anticipate that *T. helenitis* will go extinct in Europe.

## Graphical abstract

*Tephroseris helenitis* has experienced a severe decline in records over the last 120 years in the state Hessia, Germany. In our statistical modeling study, we make it likely that ongoing climate change is responsible for its decline by affecting flower induction and seed germination. Increasing March and April temperatures as well as autumn and winter temperatures between 1900 and 2020 may imply that vernalization became increasingly unsuccessful for the species, and increasing July temperatures may have decreased its germination success.

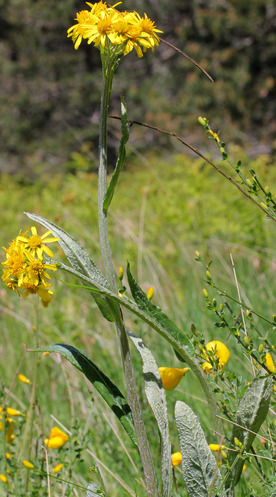

## 1 INTRODUCTION

It is beyond reasonable doubt that anthropogenic global change strongly affects biodiversity (IPBES, 2019; IPCC, 2022). Considering global warming, organisms potentially can respond (1) by tracking their original niches through the founding of new populations in more or less adjacent areas (depending on the dispersal/migration capability of species) matching their ecological requirements, which in turn results in changes of geographical distribution, (2) by changing their niche (i.e., shifting and/or broadening) either through plastic responses or evolutionary adaptation or (3) by going extinct (Wiens, 2016). Although changes in geographical distribution, either elevationally or latitudinally, have been observed in thousands of species (Moritz & Agudo, 2010; Parmesan, 2006), niche changes by evolutionary adaptation (Jump & Peñuelas, 2005) in most cases probably is too slow to keep pace with environmental change (Jezkova & Wiens, 2016; Quintero & Wiens, 2013). However, a more important role for adaptation (in combination with dispersal) has recently been suggested by Román‐Palacios and Wiens (2020). With respect to extinction, it has been pointed out repeatedly that establishment of a causative relationship between local extinction and high temperatures is difficult (Cahill et al., 2013). Although Wiens (2016) found that local extinction is related to climate in 47% of the 976 species surveyed by him, identification of proximate causes remains difficult (Cahill et al., 2013; Moritz & Agudo, 2010). In 136 case studies analyzed by Cahill et al. (2013), only seven identified anthropogenic climate change as proximate cause of local extinction, and none showed a straightforward relationship between extinction and limited tolerance of high temperature. In a recent analysis of temperature effects on altogether 538 species, Román‐Palacios and Wiens (2020) found that extinctions are better explained by hottest annual temperatures than by mean annual temperatures or other climate‐related variables. However, separating a direct effect of high temperature from indirect effects such as changed biotic interactions (Ockendon et al., 2014) will remain difficult and requires long‐term observational and experimental approaches (Harte et al., 2015; Panetta et al., 2018).

*Tephroseris* (Asteraceae: Senecioneae) is a flowering plant genus containing seven species in Europe outside Russia according to the latest revision available (Kadereit et al., 2021). Several European red lists record the decline of essentially all species of this genus. Many authors explained the decline of *Tephroseris* in Europe by habitat loss or habitat modification (Isaakson, 2009; Martínez‐García et al., 2012; Meindl, 2010, 2011; Pflugbeil, 2012; Stroh et al., 2017), whereas Kadereit et al. (2021), based on the observation that the decline of these species gathered pace near the end of the 19th century, hypothesized a role for rising temperatures.

**FIGURE 1:**
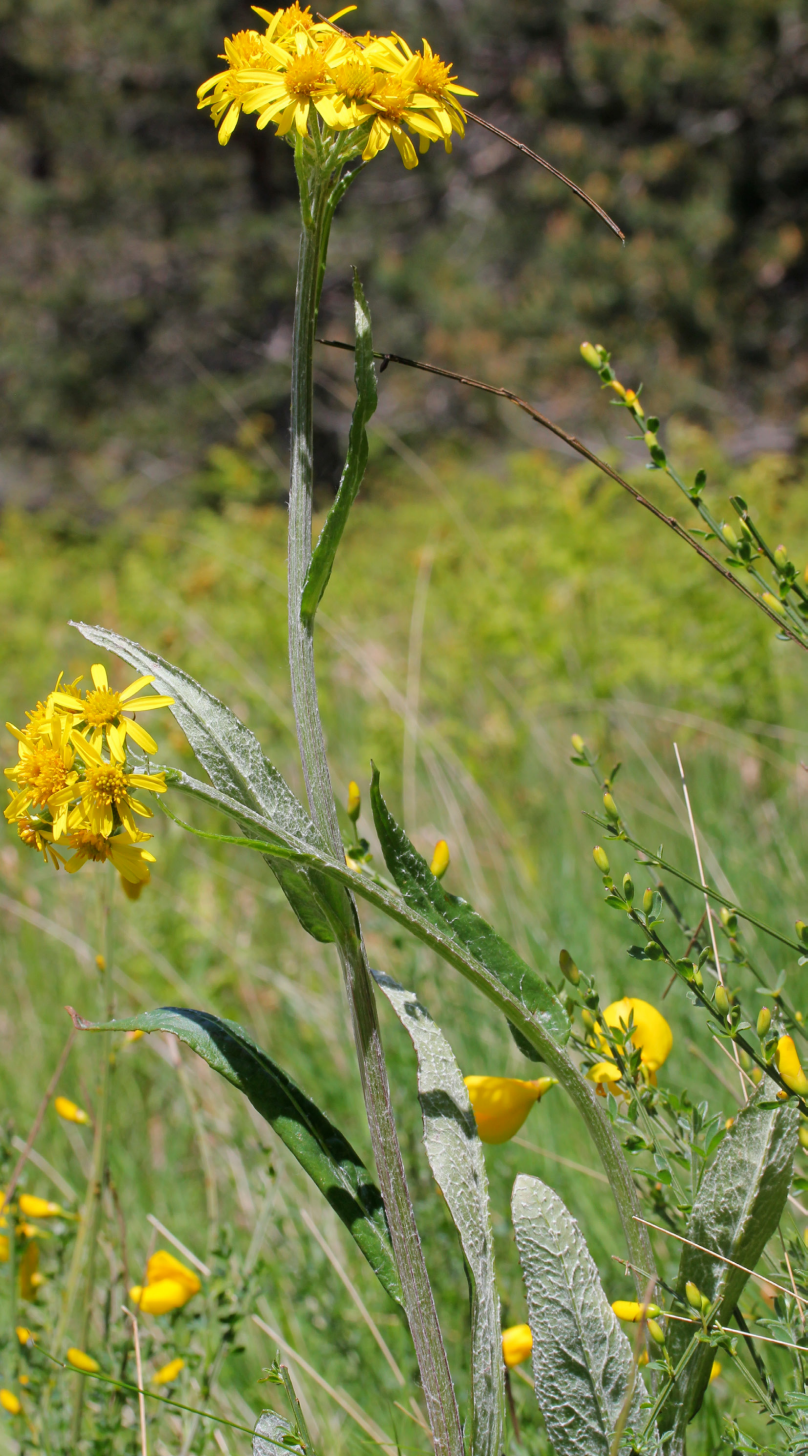
*Tephroseris helenitis*. Picture from Wikimedia (license cc‐by‐sa‐2.0‐fr; https://upload.wikimedia.org/wikipedia/commons/9/94/Tephroseris_helenitis_flower_%2809%29.jpg; by Marie Portas).

*Tephroseris helenitis*, one of the species studied by Kadereit et al. (2021), is a perennial herbaceous flowering plant (Figure 1). It has been assessed as endangered in Germany, and as vulnerable, endangered, critically endangered or in danger of extinction across its European range (Kadereit et al., 2021). In Germany, detailed data on the spatial distribution of *T. helenitis* over the last ca. 150 years are available for the area of the state of Hessia in its modern delimitation (Bönsel et al., 2021; Kadereit et al., 2021). These data document a steep decline of the species between 1850 and 1899. In the latest survey (Bönsel et al., 2021), the species has been recorded only in a single locality in Hessia. This long period of documentation of decline in combination with the availability of continuous climate data since 1900 for Germany, and the information available on its ecology (Brunerye, 1969; Schmidt, 1986), which is better than ecological information on most other *Tephroseris* species studied by Kadereit et al. (2021), caused us to investigate possible climatic causes of the decline of *T. helenitis* in Hessia using a statistical modeling approach.

## 2 MATERIALS AND METHODS

### 2.1 Study system

*Tephroseris helenitis* is a perennial herbaceous flowering plant. Although it is unknown how long exactly individuals live, longevity has been estimated to be high based on rhizome diameters of up to 15 mm by Schmidt (1986). Seeds of *T. helenitis* mostly germinate shortly after seed formation in summer (Schmidt, 1986). Brunerye (1969) observed no seed dormancy in his experiments, optimum germination temperature was found to range between 18 and 25°C, and no germination below 15°C was observed by him. Above 30°C seeds rot quickly (Brunerye, 1969). Spring germination has also been observed (Brunerye, 1969). Germination rate never exceeded 40% in the experiments by Brunerye (1969), who also found that seed viability decreased sharply after 4–5 years. Rosettes formed after germination always overwinter and often grow for 1 or 2 more years before first flowering (Schmidt, 1986). Following Brunerye (1969), rosettes require vernalization to induce flowering, and individuals do not flower every year (Schmidt, 1986). Brunerye (1969) hypothesized that February and March temperatures may be relevant for vernalization of rosettes. The major flowering time of the species in Germany is May to June (Müller et al., 2021; Schmidt, 1986), but flowering can start as early as March in the more southern parts of its European range (Brunerye, 1969; Kadereit et al., 2021). Flowering is independent of photoperiod (Brunerye, 1969). The major fruiting time in Germany is June to July (Schmidt, 1986), but in other parts of its European range fruiting can start as early as May and end in mid‐August. Reproduction is mainly vegetative according to Schmidt (1986), and new rosettes are formed from the axillary buds of rosette leaves.

According to Ellenberg's (1996) indicator values (EIV), given for *Tephroseris helenitis* subsp. *salisburgensis* (an intraspecific taxon not recognized by Kadereit et al., 2021; but see Pflugbeil, 2012; Pflugbeil et al., 2021), and using the verbal descriptions of indicator values as used by Hill et al. (1999), the species is light‐loving (EIV 8), grows at damp to wet (EIV 8) and more or less infertile to extremely infertile (EIV 2) sites. It is indifferent to soil pH, with soil pH values ranging between four and eight (Brunerye, 1969). While most authors characterize the growing conditions of the species in a similar way, they disagree about soil humidity, whereas Ellenberg (1996) claimed that soil humidity is constant throughout the year, other authors described soil humidity as fluctuating (wechselfeucht; Fischer et al., 2005; Landolt et al., 2010; Müller et al., 2021; Pflugbeil, 2012). Ellenberg (1996) considered the species part of extensively used *Molinia caerulea*‐meadows (Molinion; Pfeifengras‐Streuwiesen), whereas Pflugbeil (2012) more generally described the species to occur in wet meadows and fens. However, *T. helenitis* also occurs in open sites in or along the edges of wet to swampy broad‐leaved forests. Such forest sites were considered to be one of the main habitats of the species in France by Brunerye (1969).

The overall distribution of the species in Europe, based mainly on Meusel and Jäger (1992), is shown in Kadereit et al. (2021), and more detailed distribution maps are available for Germany (Bundesamt für Naturschutz (BfN), 2022), Switzerland (Info Flora, 2022) and France (Tela Botanica, 2022). *Tephroseris helenitis* has been assessed as vulnerable to endangered in Switzerland and as endangered in Austria and Germany, and regional accounts assess it as critically endangered or in danger of extinction, and as near threatened (subsp. *candida*; if recognized; see Kadereit et al., 2021) or vulnerable (subsp. *macrochaeta*; if recognized; see Kadereit et al., 2021) in France.

In Hessia, *T. helenitis* is found in humid habitats with moderate temperatures (Ellenberg, 1996). Here, records have declined substantially from a total of 103 records from before 1850 until 2009 (S. Hodvina, pers. comm.) to a single record in 2019 (Bönsel et al., 2021). At the same time, mean annual temperature has increased from 8.02°C in the period 1900–1949 to 9.34°C in 2000–2020 (Figure S1), whereas mean annual precipitation sum was rather similar in these two periods (765 mm in 1900–1949, 782.9 mm in 2000–2020, Figure S1). Those sites in Hessia where the species went extinct recently (2001, 2007) were forest sites (Bönsel et al., 2018, 2021).

### 2.2 Our modeling approach

To assess changes in climatic conditions over the past 120 years and to test our hypothesis that changes caused the severe decline of records of *T. helenitis* in Hessia, we conducted several analyses. All were carried out for an area defined by species records from before 1850 and the period 1850–1899 (Kadereit et al., 2021) and for grid cells without any species record (absences) and cells with at least one record (presences). We first analyzed how 62 climatic variables had changed from 1900 until the present in this area. All analyses of the effect of climate on the species' distribution were limited to the two periods 1900–1949 and 1950–1979. The period 2000–2020 was used for model validation. To assess which of the 62 climatic variables can explain presences and absences of *T. helenitis* in the periods 1900–1949 and 1950–1979, respectively, we established differences in climatic variables between presences and absences and we modeled the climatic niche of the species by logistic regression analysis. To identify possible shifts in the realized climatic niche of *T. helenitis* between the periods 1900–1949 and 1950–1979 for each of the 62 climatic variables, we compared their medians for presences of the species between the periods as well as their thresholds on presences calculated from the logit curves. For presences, we further tested changes in variances between both periods for all climatic variables, which would indicate a narrowing or broadening of the realized niche of the species with respect to a climatic variable, irrespective of whether the change in niche breadth comes along with a niche shift or not. All statistical analyses were conducted in the software R 4.2.1. We used the R packages *corrplot* for Spearman rank correlation analysis (Wei et al., 2018) and *ggplot2* for data visualization (Wickham et al., 2023).

### 2.3 Climatic variables

We retrieved the climatic variables used in this study from the website “opendata.dwd.de” (Deutscher Wetterdienst, DWD). The DWD provides monthly values for mean, minimum and maximum air temperature as well as precipitation sum for every year starting in 1900 for Germany. All 48 climatic variables are given at a spatial resolution of 1 km × 1 km grid cells. However, such small grid cells result in substantial spatial autocorrelation of climatic variables and their usage can result in model overfitting. We therefore first established grids of a larger spatial resolution for all 48 climatic variables for every year between 1900 and 2020 in QGIS Version 3.6. The size of our grid cells corresponds to quadrants of ordinance survey maps (QTK25, ca. 5.5 km × 5.5 km). From the respective monthly values, we next calculated QTK25 grids for monthly temperature ranges (maximum – minimum air temperature of a month), annual mean temperature and annual sum of precipitation for every year. This yielded further 14 climatic variables used in this study. From all grids (62 climatic variables × 120 years), we finally created 62 climatic variables for the whole of Germany for four periods, i.e., 1900–1949, 1950–1979, 1980–1999 and 2000–2020. In the following, we abbreviate these variables as Precip_Jan through Precip_Dec, Precip_Year, Tmean_Jan through Tmean_Dec, Tmean_Year, Tmax_Jan through Tmax_Dec, Tmin_Jan through Tmin_Dec and Tmax‐Tmin_Jan through Tmax‐Tmin_Dec (Table S1).

### 2.4 Study area, presences and absences

Our analysis on whether changes in climatic conditions over the past 120 years can explain the decline of records of *T. helenitis* in Hessia was based on two literature sources (Bönsel et al., 2021; Kadereit et al., 2021) providing recent and past species records. While Kadereit et al. (2021) showed records for five periods (before 1850, 1850–1899, 1900–1949, 1950–1979, 1980–2009), Bönsel et al. (2021) showed records for only three periods (before 1949, 1950–1999, 2000 onwards). In order to maximize the length of the period in which the species declined in the past, and to include the substantial changes in climate after the turn of the millennium in Germany and Hessia (Figure S1), we chose the periods 1900–1949, 1950–1979 and 2000–2020 for our modeling study. The number of presences for 1980–2009 (seven QTK25s with at least one species record; Kadereit et al., 2021) was too small for a statistical modeling of an influence of climatic changes on the species' decline.

The area modeled was restricted to 55 (our study area) of 892 QTK25s covering Hessia. In these 55 QTK25s, the species was present before 1850 and in the period 1850–1899 (Kadereit et al., 2021). Information on presences and absences of the species in a QTK25 was generated for two periods, i.e., for 1900–1949 and 1950–1979 using the species distribution map in Kadereit et al. (2021). To establish QTK25s with presences and absences from this map for a period, we first determined how many species records are located in each QTK25 of the study area. We then used all QTK25s with at least one species record as presences and all QTK25s with no record as absences. Presences in 1900–1949 (1950–1979) were those QTK25s in which the species was present after 1900 (1950), and absences were those QTK25s in which the species was extinct in 1900–1949 (1900–1979). This resulted in 19 presences and 36 absences for 1900–1949 and in 10 presences and 45 absences for 1950–1979. For 2000–2020, for which presences were taken from Bönsel et al. (2018, 2021), three presences and 52 absences were used.

### 2.5 Climatic changes between the four periods

We first analyzed whether changes in each of the 62 climatic variables had occurred over the past 120 years in the study area. For this, a Friedman test (non‐parametric ANOVA for paired samples) across the four periods (1900–1949, 1950–1979, 1980–1999, 2000–2020), followed by pairwise Wilcoxon posthoc tests with Bonferroni correction, was performed for each of the 62 climatic variables.

### 2.6 Modeling the climatic niche of *T. helenitis*

In order to identify climatic variables' best explaining presences and absences in 1900–1949 (1950–1979), we first checked for differences in medians of the 62 climatic variables between presences and absences. This was done with Wilcoxon tests.

**FIGURE 2:**
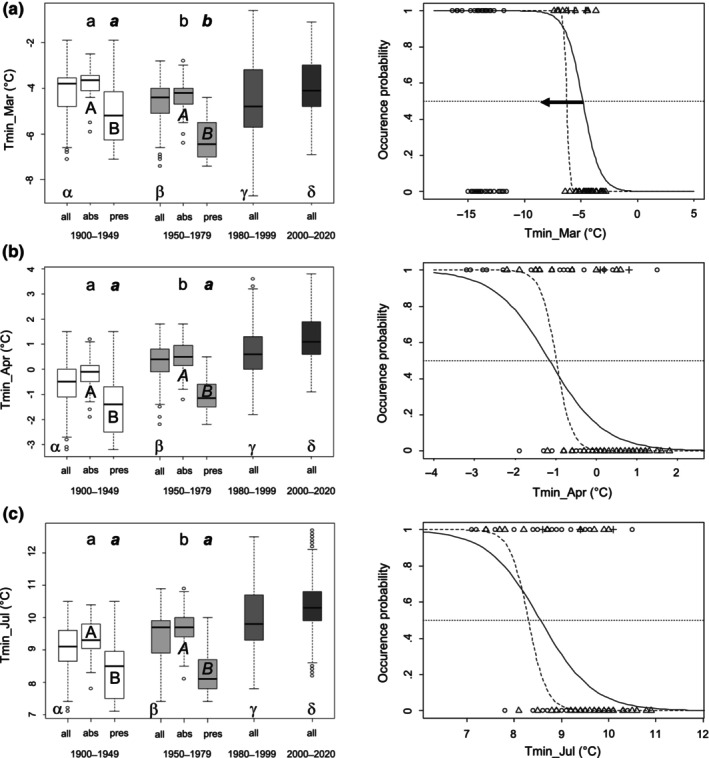
Changes in climatic variables and fitted logit curves for the overall best climatic variables. (a) Tmin_Mar, (b) Tmin_Apr, and (c) Tmin_Jul. Boxplots in the left column show climatic conditions in the entire study area for four periods (all) and only for 1900–1949 and 1950–1979 for QTK25s with presences (pres) and with absences (abs). Greek letters indicate results of pairwise Wilcoxon posthoc tests with Bonferroni correction for changes in the climatic variable over the past 120 years in our study area (all, Table S2). Differences in medians between absences and presences for both periods were assessed by Wilcoxon tests (Tables S3 and S4) and significance is indicated by uppercase letters for 1900–1949 and italics uppercase letters for 1950–1979. Differences in absences (presences) between 1900–1949 and 1950–1979 were assessed by Wilcoxon tests and significance is given by lowercase letters for absences and italics lowercase letters for presences (Table S10). Plots in the right column show fitted logit curves for occurrence probability against the climatic variable for 1900–1949 (solid line) and 1950–1979 (dashed line). Dotted line = occurrence probability is .5 (threshold), circles = presences/absences in 1900–1949, triangles = presences/absences in 1950–1979, crosses = presences in 2000–2020. For Tmin_Mar, a shift in the realized climatic niche (arrow) towards lower temperatures is statistically supported, but no change in niche breadth (Table 2, Table S10). For Tmin_Apr and Tmin_Jul, a narrowing of the realized climatic niche is significant and marginally significant, respectively, but not a niche shift (Table 2, Table S11).

Next, we modeled the climatic niche of the species with a univariate logistic regression analysis conducted for each of the 62 climatic variables and each of the periods 1900–1949 and 1950–1979. We did not perform multivariate logistic regression analysis because in both periods many and especially those climatic variables that finally yielded good models (Table 1) showed high pairwise intercorrelation (Figure S2, |*r*_Spearman_| > .7, Fielding & Haworth, 1995). Our univariate logistic regression analysis related presences and absences of a period (binary information) to each of the climatic variables and predicts the occurrence probability (between 0 and 1) of the species from a climatic variable. Specifically, we fitted a linear logit model (hereafter climatic model) using all presences (coded as 1) and absences (0) as values on the response variable and the climatic values of the respective QTK25s as values on the predictor variable. All climatic models of a period for which the fitted linear logit model had a significant slope (the predictor) and a significant likelihood ratio test were passed to an AIC‐based model selection process (Burnham & Anderson, 2002) in order to identify the climatic model(s) which best predicted all presences and absences of a period. The accuracy of the 62 climatic models established for each of the periods 1900–1949 and 1950–1979 was assessed by sensitivity (percentage of correctly predicted presences of the period), specificity (percentage of correctly predicted absences of the period) and correct classification rate (sensitivity + specificity). To establish the three rates, we first calculated a threshold value for each climatic model and period, which was the climatic value for which the model calculates an occurrence probability of .5. Then, these climatic thresholds were used to infer whether each species' presence and absence, respectively, were correctly predicted from the value of the respective climatic variable in its QTK25 (e.g., the Tmin_Mar value of the QTK25 with a presence was indeed smaller than the threshold and it was larger than the threshold for an absence, respectively, Figure 2). Finally, for model validation, all climatic models for both periods were applied to the period 2000–2020. Specifically, the occurrence probability for the three presences and 52 absences in 2000–2020 was calculated from each climatic model of the periods 1900–1949 and 1950–1979. Then sensitivity and specificity rates were calculated for each model for the three presences and 52 absences by using again an occurrence probability of .5 for thresholds of models and periods. We did not calculate the correct classification rates of models, because in the period 2000–2020, the proportion of presences and absences was strongly biased towards absences.

**TABLE 1:** Summary of differences in climatic variables and modeling of *T. helenitis* in the study area.

Predictor	1900–1949 versus 1950–1979 (all)	1950–1979 versus 2000–2020 (all)	1900–1949 pres versus abs	1900–1949 ΔAIC (LR)	1900–1949 sens/spec in 2000–2020	1950–1979 pres versus abs	1950–1979 ΔAIC (LR)	1950–1979 sens/spec in 2000–2020
Tmean_Jan	n.s.	<	<	11.275		<	**0.895**	1/51
Tmean_Feb	n.s.	<	<	11.354		<	**1.528**	1/47
Tmean_Mar	>	<	<	11.055		<	**2.341**	1/50
Tmean_Apr	n.s.	<	<	11.053		<	**5.340**	1/47
Tmean_May	>	<	<	10.852		<	**5.686**	1/51
Tmean_Jun	<	<	<	10.808		<	**6.082**	1/46
Tmean_Jul	>	<	<	11.264		<	**7.139**	1/51
Tmean_Aug	n.s.	<	<	11.392		<	**5.255**	1/51
Tmean_Sep	n.s.	<	<	12.069		<	**3.936**	1/47
Tmean_Oct	<	<	<	11.946		<	**0**	1/47
Tmean_Nov	<	<	<	11.756		<	**1.638**	1/47
Tmean_Dec	<	<	<	12.530		<	**1.960**	1/49
Tmax _Feb	n.s.	<	<	13.612		<	**6.282**	1/52
Tmax _Mar	>	<	<	15.199		<	**1.263**	1/45
Tmax _Apr	>	<	<	17.863		<	**2.479**	2/52
Tmax _May	>	<	<	18.031		<	**5.852**	1/28
Tmax _Jun	>	<	<	15.515		<	**6.574**	2/52
Tmax _Jul	<	<	n.s.	n.s.		<	**5.170**	0/51
Tmax _Sep	>	<	<	n.s.		<	**5.709**	1/52
**Tmin_Mar**	>	<	<	**2.966**	3/36	<	n.s.	2/45
**Tmin_Apr**	<	<	<	**3.616**	0/51	<	**5.343**	0/51
Tmin_May	<	<	<	12.203		<	**8.583**	1/50
Tmin_Jun	<	<	<	**6.881**	1/51	<	14.656	
**Tmin_Jul**	<	<	<	**4.586**	1/50	<	**5.203**	1/51
Tmin_Sep	<	<	<	**4.933**	0/52	<	28.725	
Tmin_Oct	<	<	<	**8.528**	0/52	<	24.154	
Tmin_Dec	n.s.	<	<	**0**	0/52	<	17.260	
Tmax‐Tmin_Oct	>	<	>	**9.137**	0/52	<	25.245	

*Note*: Across the two periods 1900–1949 and 1950–1979, these 28 climatic variables revealed significant logistic regression models (LR) with ΔAIC < 10 at least for one period. “all” refers to the 55 QTK25 in which the species was present before 1900 (our study area). n.s. *p* > .05. ΔAIC values smaller than 10 are in bold. Tmin_Mar, Tmin_Apr and Tmin_Jul revealed good models for species presence and absence for both periods (in bold, see text). “1900–1949 versus 1950–1979 (all)” and “1950–1979 versus 2000–2020 (all)”: climatic changes in the study area, results of pairwise Wilcoxon posthoc tests with Bonferroni correction of differences in medians of respective periods (Table S2 and Figure S3). “<” (“>”) = median of 1900–1949 (1950–1979) is significantly smaller (larger) than the median of 1950–1979 (2000–2020). “1900–1949 pres versus abs”: results of Wilcoxon tests of differences in medians of presences and absences from 1900 to 1949, “<” (“>”) = median of presences is significantly smaller (larger) than that of absences (Table S3 and Figure S3). “1900–1949 ΔAIC (LR)”: ΔAIC value of the respective LR model, period 1900–1949. “1900–1949 sens./spec. in 2000–2020”: number of correctly predicted presences (sens.) and absences (spec.) when applying the 1900–1949 model to the three records from 2000 to 2020, both are based on an occurrence probability of .5 that was used to discriminate between absences and presences (Table S9). Only given for models with ΔAIC < 10. “1950–1979 pres versus abs,” “1950–1979 ΔAIC (LR),” and “1950–1979 sens/spec in 2000–2020”: as described above for 1900–1949, but see Table S4 for statistics on median differences between presences and absences of 1950–1979.

### 2.7 Assessment of changes in the realized climatic niche of *T. helenitis*

To detect changes in the realized climatic niche of *T. helenitis* between the periods 1900–1949 and 1950–1979, we compared values of climatic variables of its presences for all climatic variables applying Wilcoxon tests of median differences (i.e., a niche shift, irrespective of whether this comes along with a change in niche breadth) and Ansari‐Bradley tests of variance homogeneity (i.e., niche broadening or narrowing, irrespective of whether this comes along with a niche shift). We also inspected changes in climatic thresholds (occurrence probability = .5) from the logit curves between the two periods for all climatic variables, which also could indicate a niche shift. Finally, we compared values of climatic variables of absences between both periods for all climatic variables using Wilcoxon tests in order to find out whether climatic conditions had changed in QTK25s in which the species was present in 1900–1949 but not in 1950–1979.

### 2.8 Distribution of *T. helinitis* in Hessia and Germany for 2000–2020

We used our final best climatic models (three for each of the two periods 1900–1949 and 1950–1979) to visualize habitat suitability in 2000–2020 for Hessia and Germany. For this purpose, each climatic model was evaluated for all QTK25s comprising Hessia and Germany, respectively to predict the species' occurrence probability in each QTK25 from the respective climatic value.

## 3 RESULTS

### 3.1 Climatic changes between the four periods

Friedman tests of differences in medians of climatic variables across periods (1900–1949, 1950–1979, 1980–1999, 2000–2020) were highly significant for all 62 variables (Table S2) and indicated substantial climatic changes in the study area throughout the past 120 years. Pairwise Wilcoxon posthoc tests with Bonferroni correction further showed significant differences between all four periods for the majority of variables (Table S2 and Figure S3). Of all 372 (= 62 × 6) pairwise comparisons, only 21 were not significant. Medians of all climatic variables differed significantly between 1900–1949 and 1980–1999 and between 1980–1999 and 2000–2020. For 1950–1979 and 2000–2020, median differences were all significant except for one variable, for 1950–1979 and 1980–1999 except for two and for 1900–1949 and 2000–2020 except for four variables. Fourteen variables did not differ between 1900–1949 and 1950–1979 (Table S2 and Figure S3).

### 3.2 Modeling the climatic niche of *T. helenitis*

For the period 1900–1949, medians of presences and absences did not differ significantly for all 13 precipitation‐related variables, whereas those of the majority of temperature‐related variables differed (Table S3 and Figure S3). In particular, medians were different for Tmean_Jan through Tmean_Dec and for Tmean_Year. For monthly maximum temperatures, they were not different for three variables (Tmax_Jan, Tmax_Oct, Tmax_Nov), for monthly minimum temperatures for two (Tmin_Jan, Tmin_Feb) and for monthly temperature ranges for nine (for Tmax‐Tmin_Apr, Tmax‐Tmin_Oct and Tmax‐Tmin_Nov medians were significantly different; Table S3 and Figure S3). For the period 1950–1979, only medians for three (Tmax‐Tmin_Mar, Tmax‐Tmin_Nov, Tmax‐Tmin_Dec) of the 62 climatic variables did not differ significantly between presences and absences (Table S4 and Figure S3).

Temperature and precipitation‐related climatic models showed complementary logit curves for both periods. While occurrence probabilities always increased for Precip_Jan through Precip_Dec and for Precip_Year with increasing precipitation, they decreased with increasing temperatures for the majority of the 49 temperature‐related variables (Figures S4 and S5).

Of the 62 climatic models applied to presences and absences for 1900–1949, both the slopes of the fitted logit curves and the likelihood ratio tests were significant for 35 models (Table S5 and Figure S4). The climatic model with the smallest AIC value was Tmin_Dec, and seven other temperature‐related models had ΔAIC values smaller than 10 (Table 1 and Table S5). Of these eight models, seven used monthly minimum temperatures (Tmin_Mar through Tmin_Jul, Tmin_Sep, Tmin_Oct) and one a monthly temperature range (Tmax‐Tmin_Oct, largest ΔAIC value). Sensitivities of these eight models ranged from 47.37% to 63.16%, specificities from 88.89% to 94.44%, and their correct classification rates from 76.36% to 83.64% (Table S6). Only the Tmin_Mar model correctly predicted the three presences in the period 2000–2020, but its specificity was the smallest (36 of 52 absences correctly predicted) among these eight models for 1900–1949 (Table 1 and Table S9). Conversely, models Tmin_Jun and Tmin_Jul predicted only one presence correctly, but correctly predicted absences in 51 and 50 QTK25s, respectively (Table 1 and Table S9). The remaining models Tmin_Sep, Tmin_Oct, Tmin_Dec and Tmax‐Tmin_Oct did not predict any presences but all 52 absences (Table 1 and Table S9).

For the period 1950–1979, the slopes of the fitted logit curves and the likelihood ratio tests were significant for the majority of climatic models (51 of 62 models, Table S7 and Figure S5). The climatic model with the smallest AIC value was Tmean_Oct, and 21 others had ΔAIC values smaller than 10 (Table 1 and Table S7). With ΔAIC values smaller than two, five models (Tmean_Jan, Tmean_Feb, Tmean_Nov, Tmean_Dec, Tmax_Mar) were as well supported as the Tmean_Oct model. Of these 22 models, all monthly mean temperature models as well as seven monthly maximum temperature models (Tmax_Feb through Tmax_Jul, Tmax_Sep) and three monthly minimum temperature models (Tmin_Apr, Tmin_May, Tmin_Jul) had ΔAIC values smaller than 10. Their sensitivities ranged from 60% to 70%, specificities from 95.56% to 100% and correct classification rates from 80% to 94.54% (Table 1 and Table S8). Of the 22 climatic models with ΔAIC values smaller than two, two models (Tmax_Jul, Tmin_Apr) predicted none of the three presences, 18 models predicted one presence, and two models (Tmax_Apr, Tmin_Mar) predicted two presences in 2000–2020 (Table 1 and Table S9). The Tmax_May model correctly predicted the smallest number of absences (28), whereas all other models predicted 45–52 absences in 2000–2020 (Table 1 and Table S9).

Across the two periods 1900–1949 and 1950–1979, 28 climatic models had ΔAIC values smaller than 10 and all were temperature‐related (Table 1). Of these 28 models, only the Tmin_Apr and Tmin_Jul models were shared between the two periods (Table 1). The Tmin_Apr and Tmin_Jul models had small AIC values for both periods (Table 1). Both models had a sensitivity rate of 52.65% (60%), a specificity rate of 91.67% (97.78%) and a correct classification rate of 78.18% (90.91%) for the period 1900–1949 (1950–1979; Tables S6 and S8). Neither the Tmin_Apr model for 1900–1949 nor that for 1950–1979 predicted any of the three presences in 2000–2020, but both predicted all 52 absences (Table 1 and Table S9). The Tmin_Jul model for 1900–1949 also did not predict any presence in 2000–2020 and predicted all 52 absences. The Tmin_Jul model for 1950–1979 predicted one presence and 51 absences for the period 2000–2020 (Table 1 and Table S9).

However, the Tmin_Mar model met our two criteria for goodness of fit only for the period 1900–1949, but not for 1950–1979. While for 1950–1979 the likelihood ratio test was highly significant (1.5 × 10^−9^), the *p*‐value of the slope of the logit curve was .173 (Table S7). The Tmin_Mar model for 1950–1979 had an AIC value only slightly smaller than that of the best model Tmean_Oct (Table 1 and Table S7) and showed a good discrimination power for presences and absences with a sensitivity rate of 70%, a specificity rate of 97.78% and a correct classification rate of 92.72%. These rates were all larger than the rates of the Tmin_Apr and Tmin_Jul models (Table S8). It also predicted two of three presences and 45 of 52 absences in 2000–2020 (Table 1 and Table S9). All this qualifies Tmin_Mar as another good model for species presence and absence in QTK25s throughout the past 120 years in addition to the statistically well‐supported Tmin_Apr and Tmin_Jul models.

### 3.3 Changes in the realized climatic niche of *T. helenitis*

The differences in medians of presences for the periods 1900–1949 and 1950–1979 were significant for 42 of the 62 climatic variables tested, whereas for absences differences of only 33 variables were significant (Table S10 and Figure S3). Variance inhomogeneity of presences was supported statistically for 17 of the 62 climatic variables (Table S11). For six further climatic variables, *p*‐values of variance homogeneity tests were at least marginally significant (<.1, Table S11). Of the 28 variables with ΔAIC values smaller than 10, 16 variables also had significantly different medians for presences and 14 variables for absences (Table 2 and Table S10). For presences, all medians of mean monthly temperatures were higher in 1900–1949 than in 1950–1979, but there was no statistical support for 4 months (Table 2 and Table S10). Consistent with differences in medians, the climatic thresholds of the logit curves indicated presences for higher temperatures in 1900–1949 compared to 1950–1979, i.e., thresholds were smaller in 1950–1979 than in 1900–1949 (Table 2 and Tables S6 and S8). Tests on variance homogeneity indicated no difference between presences for 1900–1949 and 1950–1979 for all monthly mean temperature variables (Table 2 and Table S11). For three monthly mean temperature variables, medians of absences differed between periods, but there was no consistent pattern in the change of the median across these three variables (Table 2).

**TABLE 2:** Summary of results for changes in the realized climatic niche of *T. helenitis*.

Predictor	1900–1949 Thr. (LR)	1950–1979 Thr. (LR)	m_presence_ 1950–1979 versus 1900–1949	m_absence_ 1950–1979 versus 1900–1949	var_presence_ 1950–1979 versus 1900–1949
Tmean_Jan	−1.021	−1.721	<	n.s.	n.s.
Tmean_Feb	−0.236	−0.857	<	n.s.	n.s.
Tmean_Mar	3.026	2.214	<	n.s.	n.s.
Tmean_Apr	6.821	6.081	<	n.s.	n.s.
Tmean_May	11.661	10.611	<	<	n.s.
Tmean_Jun	14.399	13.990	n.s.	n.s.	n.s.
Tmean_Jul	16.130	15.415	<	n.s.	n.s.
Tmean_Aug	15.398	14.859	n.s.	n.s.	n.s.
Tmean_Sep	12.378	11.930	<	n.s.	n.s.
Tmean_Oct	7.685	7.697	n.s.	>	n.s.+
Tmean_Nov	2.905	2.634	n.s.	>	n.s.
Tmean_Dec	0.013	−0.473	<	n.s.	n.s.
Tmax _Feb	7.989	7.526	n.s.	n.s.	n.s.
Tmax _Mar	12.543	11.526	<	<	n.s.
Tmax _Apr	16.734	15.575	<	<	n.s.+
Tmax _May	21.199	19.942	<	<	<
Tmax _Jun	24.660	22.852	<	<	<
Tmax _Jul	25.424	25.401	<	n.s.	n.s.
Tmax _Sep	23.428	21.631	<	<	<
**Tmin_Mar**	−4.838	−6.253	<	<	n.s.
**Tmin_Apr**	−1.153	−0.985	n.s.	>	<
Tmin_May	2.574	2.467	n.s.	n.s.	<
Tmin_Jun	6.117	6.380	n.s.	>	n.s.+
**Tmin_Jul**	8.558	8.301	n.s.	>	n.s.+
Tmin_Sep	4.247	3.891	n.s.	>	n.s.
Tmin_Oct	0.610	0.262	n.s.	n.s.	n.s.
Tmin_Dec	−7.737	−9.228	n.s.	n.s.	n.s.
Tmax‐Tmin_Oct	16.933	12.960	<	<	<

*Note*: Across the two periods 1900–1949 and 1950–1979, these 28 climatic variables revealed significant logistic regression models (LR) with ΔAIC < 10 at least for one period (Table 1). Tmin_Mar, Tmin_Apr and Tmin_Jul revealed good models for species presence and absence for both periods (in bold, see text). Significance levels: n.s. *p* > .05, n.s. + .1 ≥ *p* > .05. “1900–1949 Thr. (LR)” = model threshold for 1900–1949 and “1950–1979 Thr. (LR)” = model threshold for 1950–1979: niche shift, each model threshold reveals an occurrence probability of .5 for the respective LR. “m_presence_ 1950–1979 versus 1900–1949”: niche shift, results of Wilcoxon tests of differences in medians of presences from 1900 to 1949 and 1950 to 1979, “<” = median of 1950–1979 is smaller than median of 1900–1949 (Table S10 and Figure S3). “m_absence_ 1950–1979 versus 1900–1949”: niche shift, results of Wilcoxon tests of differences in medians of absences from 1900 to 1949 and 1950 to 1979, “<” (“>”) = median of 1950–1979 is smaller (larger) than median of 1900–1949 (Table S10 and Figure S3). “var_presence_ 1950–1979 versus 1900–1949”: change in niche breadth, results of Ansari‐Bradley tests of variance homogeneity of climatic conditions observed for presences from 1900 to 1949 and 1950 to 1979, “<” = variance of presences for 1950–1979 is significantly smaller than that for 1900–1949 (Table S11 and Figure S3).

For presences, medians of six of seven monthly maximum temperatures (not Tmax_Feb, Table 2 and Table S10) were significantly higher in 1900–1949 than in 1950–1979, and thresholds were higher in 1900–1949 than in 1950–1979 for all seven variables (Table 2). For presences, the variance in Tmax_May, Tmax_Jun and Tmax_Sep values was significantly larger in 1900–1949 than in 1950–1979, whereas variances did not differ statistically for all other monthly maximum temperature variables (Table 2 and Table S11). For the six variables with significant medians found for presences, the medians of absences were higher in 1900–1949 than in 1950–1979 (Table 2 and Table S10).

Of the eight climatic variables related to monthly minimum temperatures, the medians of presences of the two periods differed statistically only for Tmin_Mar. For Tmin_Mar the median was significantly smaller in 1950–1979 than in 1900–1949 (Table 2 and Table S10), and the threshold was smaller in 1950–1979 than in 1900–1949 (Table 2 and Tables S6 and S8). The variance in Tmin_Apr and Tmin_May values was significantly larger in 1900–1949 than in 1950–1979 for presences, but variances did not differ significantly for all other monthly minimum temperature variables (Table 2 and Table S11). Five monthly minimum temperature variables showed significant differences in medians in these two periods for absences (Table 2 and Table S10). For Tmin_Mar the median was smaller in 1950–1979 than in 1900–1949 for absences, whereas medians were larger in 1950–1979 than in 1900–1949 for the four others Tmin_Apr, Tmin_Jun, Tmin_Jul and Tmin_Sep (Table 2 and Table S10).

For Tmax‐Tmin_Oct, the medians of both presences and absences were significantly larger in 1950–1979 than in 1900–1949, and for presences, the variance in values was significantly larger in 1900–1949 than in 1950–1979 (Table 2 and Tables S10 and S11).

### 3.4 Summary of results

We found considerable changes in climatic variables across the four periods 1900–1949, 1950–1979, 1980–1999 and 2000–2020 (Table S2 and Figure S3). Consistent with the hypothesis by Kadereit et al. (2021), we also found multiple evidence that these climatic changes affected the realized ecological niche of *T. helenitis* in our study area throughout time and in particular for climatic variables yielding good models in terms of ΔAIC values on species presence and absence in 1900–1949 and 1950–1979 (Table 1). Climatic effects on presences or absences of *T. helenitis* were observed for 22 of the 28 variables with ΔAIC values smaller than 10 either for the 1900–1949 or the 1950–1979 period (Table 2). Specifically, the substantial changes in climatic variables between the two periods were corroborated for presences (absences) by significant differences in medians for 16 (14) of the 28 variables (Table 2). Thresholds of presences calculated by climatic models were larger in 1900–1949 than in 1950–1979 for 21 of the 22 climatic variables (except for Tmean_Oct, Table 2), and this was consistent with significantly smaller medians of presences in 1950–1979 than in 1900–1949 for 15 climatic variables (except for Tmean_Oct, Table 2). For presences, smaller variances in 1950–1979 than in 1900–1949 were significant for six climatic variables (Table 2). All changes in medians of climatic variables observed for presences and in the thresholds of climatic models indicated a shift in the realized niche of *T. helenitis* for the respective climatic variable between 1900–1949 and 1950–1979 (Table 2). The smaller variances found in 1950–1979 than in 1900–1949 indicated a narrowing of the realized niche of *T. helenitis* for the respective climatic variables between both periods (Table 2).

The Tmin_Apr and Tmin_Jul model shared between the periods 1900–1949 and 1950–1979 were good models in terms of small AIC values for both periods (Table 1). In terms of sensitivities and specificities, both models had a good discrimination power for presences and absences in 1900–1949 and 1950–1979, but the power of the Tmin_Mar model was slightly higher (Tables S6 and S8). The Tmin_Mar model for the period 1900–1949 even correctly predicted all three presences in 2000–2020 and the Tmin_Mar model for 1950–1979 two, whereas the Tmin_Apr and Tmin_Jul models for 1900–1949 and 1950–1979 correctly predicted one presence at the most (Table 1 and Table S9). For the Tmin_Apr and Tmin_Jul models, insignificant differences in medians of presences and rather similar thresholds of logit curves indicated no shift in the realized ecological niche between 1900–1949 and 1950–1979 for both climatic variables (Table 2 and Figure 2), but smaller variances in 1950–1979 than in 1900–1949 indicated a narrowing of the realized niche for both variables (Table 2 and Figure 2). However, the variance inhomogeneity was only marginally significant for Tmin_Jul (Table 2). Conversely, for Tmin_Mar significant differences in medians of presences and changes in thresholds of logit curves indicated a shift in the realized niche towards smaller Tmin_Mar values, whereas the homogeneous variances suggested that this shift was neither accompanied by a niche broadening nor a narrowing between 1900–1949 and 1950–1979 (Table 2 and Figure 2).

### 3.5 Distribution of *T. helinitis* in Hessia and Germany for 2000–2020

The habitat suitability (occurrence probability) maps for 2000–2020 established from Tmin_Mar, Tmin_Apr and Tmin_Jul for Hessia and the whole of Germany are shown in the Appendix S1 (Figure S9). When using a probability of at least .5 for species occurrence, the Tmin_Apr and Tmin_Jul models for 1900–1949 and 1950–1979 predict virtually no presence of *T. helenitis* in Hessia (Figure S9). While the Tmin_Mar model for 1900–1949 still suggests a considerable number of suitable QTK25s in northern Hessia, the model for 1950–1979 predicts only very few QTK25s (Figure S9). For the whole of Germany, only the Tmin_Mar model for 1900–1949 suggests many QTK25s being suitable for the occurrence of *T. helenitis*. These are found mainly in the eastern part of Germany. However, all six models predict high occurrence probabilities only for some mountainous regions of Germany (e.g., Alps, Black Forest, and Bavarian Forest).

## 4 DISCUSSION

Of the 62 climatic variables examined in order to explain the decline of *T. helenitis* in Hessia over the past 120 years, 28 temperature‐related variables revealed models with ΔAIC values smaller than 10 (Table 1). None of the 13 precipitation‐related variables tested was in this set of variables, although changes in medians across the periods 1900–1949, 1950–1979 and 2000–2020 were significant for several of these variables (Table S2 and Figure S3). Considering that *T. helenitis* is a species from humid habitats such as wet meadows, fens and the edges of wet to swampy broad‐leaved forests, it is somewhat surprising that changes in precipitation do not explain the decline of the species. In explanation, it seems possible that soil humidity is a microclimatic condition related more to, e.g., topography than to precipitation. Also, spatial resolution of the precipitation‐related climatic variables used in this study could be too low to describe such fine‐grained microclimatic conditions of the sites inhabited by *T. helenitis*, especially because absolute and relative changes in monthly and annual precipitation sums between periods are small, when compared to such in temperature‐related variables (Table S2 and Figure S3).

Of the 28 temperature models, the Tmin_Mar, Tmin_Apr and Tmin_Jul models best explained the distribution through time of *T. helenitis* in our study area. They worked well for the periods 1900–1949 and 1950–1979 in terms of small ΔAIC values and high sensitivity, specificity and correct classification rates (Table 1 and Tables S6 and S8). These three temperature‐related variables essentially coincide with two critical phases in the life cycle of the species.

With respect to March and April temperatures, these may well be related to the induction of flowering through a period of low temperatures, i.e., vernalization. As Brunerye (1969) hypothesized that February and March temperatures may be relevant for the vernalization of overwintering rosettes, it seems possible that increased March and April temperatures either are not sufficiently low for successful vernalization to complete (i.e., insufficient chilling), or that high March or April temperatures lead to devernalization, i.e., the reversal of vernalization which can be affected by short spells of high temperatures (Penfield et al., 2021). As the effects of low temperatures resulting in vernalization as a rule accumulate over prolonged periods in autumn and winter, temperatures in these months also are informative regarding induction of flowering. Many of our results related to temperatures in autumn and winter months are consistent with the insufficient chilling hypothesis. Median values of Tmean_Oct, Tmean_Nov and Tmean_Dec increased significantly from 1900–1949 to 1950–1979 and from 1950–1979 to 2000–2020, and medians of Tmean_Sep, Tmean_Jan and Tmean_Feb are smaller in 1900–1949 than in 1950–1979 and smaller in 1950–1979 than in 2000–2020 in the study area (Figure S3 and Table S2). Absences in 1950–1979 have significantly larger medians for Tmean_Oct and Tmean_Nov than in 1900–1949 (Figure S3 and Table S10). Median values of Tmin_Sep, Tmin_Nov and Tmin_Jan are larger for absences in 1950–1979 than in 1900–1949 (Figure S3 and Table S10). Median values of Tmax_Nov also are larger for absences in 1950–1979 than in 1900–1949 (Figure S3 and Table S10). All these differences in climatic conditions observed between periods may imply that vernalization could not be completed through autumn and winter and that low March and April temperatures are necessary to complete vernalization. In line with this conclusion, but in contrast to the devernalization hypothesis, we found that the median of Tmean_Mar is slightly but significantly smaller in 1950–1979 than in 1900–1949 and that medians of Tmean_Apr do not differ between the two periods. Especially, higher Tmax_Mar and Tmax_Apr medians in 1950–1979 than in 1900–1949, which could indicate short spells of high temperatures leading to devernalization (Penfield et al., 2021), are not significant for the study area (Figure S3 and Table S2). Increased March and April temperatures, which are also consistent with the devernalization hypothesis, are only corroborated by significantly larger median values of Tmean_Mar and Tmean_Apr for 2000–2020 compared to 1950–1979 and for Tmean_Mar compared to 1980–1999 (Figure S3 and Table S2). Conversely, an avoidance of warm March and April temperatures, which is in line with the insufficient chilling hypothesis, is further documented by both significantly smaller median values of Tmin_Mar in 1950–1979 than in 1900–1949 for absences and by significantly smaller Tmax_Mar and Tmax_Apr values for presences (Figure S3 and Table S10). In summary, it seems most likely that increasing temperatures in autumn, winter, March and April made vernalization increasingly unsuccessful for *T. helenitis* over the past 120 years due to insufficient chilling. In consequence, the species in the study area became more and more restricted to QTK25s with low temperatures.

For the closely related *Tephroseris integrifolia* (Widén, 1987), which differs from *T. helenitis* by being a species of calcareous grasslands, Meindl (2011) could show that cold temperatures during December, January and February, measured as low minimum values, increased the probability of previously flowering individuals to flower again, and of rosettes to change into a generative state in the following growing season. In contrast, mild winters resulted in a lower probability of flower formation in plants that had flowered in the previous year. These observations by Meindl (2011) on *T. integrifolia*, illustrating the negative effect of higher temperatures on flowering, clearly fit our findings for *T. helenitis*.

With respect to July temperatures, these may affect seed germination. It is well known that high temperatures can either inhibit germination (thermoinhibition) or induce dormancy (thermodormancy; Corbineau et al., 1988; Geshnizjani et al., 2018; Gorzin et al., 2021; Lafta & Mou, 2013) and that temperatures lower or higher than optimal temperatures result in decreased germination rates (Bewley et al., 2013). As Brunerye (1969) observed no dormancy in his seed germination experiments, thermodormancy due to rising temperatures seems unlikely in *T. helenitis*.

Schmidt (1986) reported that seeds are formed mainly in June and July (Schmidt, 1986) in our study area, and their optimum germination temperature was found to range between 18 and 25°C by Brunerye (1969), who also observed no germination below 15°C and found that seeds rot quickly above 30°C. Consistent with the finding of Brunerye (1969) that germination in *T. helenitis* requires temperatures higher than 15°C, the medians of Tmean_Jul were larger than 15°C for presences in the periods 1900–1949 and 1950–1979 (Tables S3 and S4 and Figure S3). Although Tmin_Jul values observed for presences were all substantially smaller than 15°C for each year of both periods, Tmean_Jul values larger than 15°C indicate that the number of years in which minimum temperatures in July potentially reduced overall germination success was small in 1900–1949 and 1950–1979. In our study area, maximum temperatures in June, July and August on average (median) exceeded the upper threshold of optimal germination of 25°C (Brunerye, 1969) in all four periods (Table S2 and Figure S3). This could indicate that at least in some of the past 120 years conditions were suboptimal for seed germination. In 2000–2020, the median value of Tmax_Jul was 29.8°C and thus only marginally smaller than 30°C. The maximum value of Tmax_Jun even exceeded 30°C in 1900–1949 (31.1°C), that of Tmax_Jul in 1980–1999 (30.1°C) and 2000–2020 (32.1°C) and that of Tmax_Aug in 2000–2020 (31.7°C, Table S2 and Figure S3).

The possibly lethal effect of temperature on seeds hypothesized by us for Tmax_Jul and Tmax_Aug in 1980–1999 and/or 2000–2020 may support Román‐Palacios and Wiens's (2020) finding that species decline (and extinction) is better explained by hottest than by mean annual temperatures.

Clearly, all hypothesized effects of higher temperatures on flowering and germination as inferred from our modeling require experimental verification. However, as *T. helenitis* is rare in Germany and other European countries, collecting seeds required for flowering and germination experiments under controlled conditions is unrealistic. Based on the temperatures the species experienced in the past 120 years in Hessia, we suggest, should vernalization and seed germination experiments be made, that for induction of flowering constant ambient temperatures tested should range from 0.5°C (lowest Tmean_Mar and Tmean_Apr value seen in a QTK25 with species records in 1900–1949 and 1950–1979) to 10°C (largest Tmean_Apr seen in a QTK25 with species records in 1900–1949 and 1950–1979). For seed germination, we suggest a temperature range from 15°C (no germination, Brunerye, 1969) to 32.1°C (maximum Tmax_Jul value, seen in 2000–2020).

For 15 climatic variables including Tmin_Mar, we found significantly smaller medians in 1950–1979 than in 1900–1949 for presences and smaller thresholds derived from fitted logit curves (Tables 1 and 2 and Tables S5, S7 and S10, Figure S3). For eight (including Tmin_Mar) of these 15 variables differences in medians between periods were also significant for absences (Table 2 and Table S10 and Figure S3). For six climatic variables including Tmin_Apr, the variance of values was significantly smaller in 1950–1979 than in 1900–1979 and for four others including Tmin_Jul this decrease was at least marginally significant (Table 2 and Table S11). Thus, it becomes evident that with respect to all these variables the realized climatic niche of *T. helenitis* in Hessia has shifted and/or narrowed, and that only colder sites of the spectrum of sites occupied in the older period were occupied in the younger period (Figure 2 and Figure S3). This finding is consistent with the observation that the last record of the species in Hessia is a beech forest site (Bönsel et al., 2021) and that those sites where the species became extinct recently (2001, 2007) also were forest or forest edge sites (Bönsel et al., 2018). As described above, *T. helenitis* is a species of both wet meadows and fens and of open sites in or along the edges of wet to swampy broad‐leaved forests. Considering that the one extant and the two most recent but now extinct records of the species in Hessia are from forest sites, its disappearance from warmer sites as revealed by our analysis corresponds to its disappearance from sites outside forests, i.e., from wet meadows and fens. Whether this is the result of a direct temperature effect (on vernalization and/or germination), the result of changes in land use, highly likely for wet meadows and fens which have suffered greatly from human influence (Ellenberg, 1996), or the result of changing biotic interactions (Ockendon et al., 2014), probably mainly competition, we do not know. However, the Tmin_Mar models for 1900–1949 and 1950–1979 correctly predict two or three records for 2000–2020 (Table 1) and thus point at a temperature effect.

Our observation that the number of presences of *T. helenitis* declined substantially between 1900 and 2020, and that its realized niche did not match changes in many climatic variables across this period (Tables 1 and 2), most probably implies that the species did not respond to rising temperatures by a niche shift and/or broadening through evolutionary adaptation in order to survive. This fits the hypothesis that in most species evolutionary adaptation probably is too slow to keep pace with environmental change (Jezkova & Wiens, 2016; Quintero & Wiens, 2013). Although we do know which sites in Hessia were occupied last by *T. helenitis*, we do not know when these sites were first colonized. Accordingly, our data do not allow us to reach any conclusion about past migration of *T. helenitis* in response to rising temperatures, i.e., whether the species tracked its original niche in our study area.

In summary, our study detected the gradual disappearance of the temperature niche for flower induction and germination of *T. helenitis* and corroborated the hypothesis by Kadereit et al. (2021) on the species' decline in Europe for our study area. Especially, Tmin_Mar, Tmin_Apr and Tmin_Jul, coinciding with flower induction and germination of the species, have changed substantially not only in the study area (Figure 2) but also across the whole of Hessia (Figures S6–S8), and habitat suitability as assessed by these three climatic models has severely decreased over the last 120 years (Figures S6–S8). Given ongoing climatic warming, we anticipate from our modeling study that *T. helenitis* will go extinct in Hessia and in the whole of Germany in the near future as in 2000–2020 only some mountainous regions (e.g., Alps, Black Forest, and Bavarian Forest) have thermal conditions suitable for flowering and germination and thus for the survival of the species (i.e., high occurrence probabilities) (Figure S9). Given the decline of *T. helenitis* in its entire European range, and that trends of change in climatic conditions as seen in Hessia are essentially the same for this much larger geographic region, we consider it highly likely that the results from our study are valid not only for Hessia but also for the entire European distribution range of the species.

## AUTHOR CONTRIBUTIONS

**Eva Maria Griebeler:** Conceptualization (equal); data curation (equal); methodology (lead); validation (equal); writing – original draft (equal); writing – review and editing (equal). **Joachim W. Kadereit:** Conceptualization (equal); data curation (equal); validation (equal); writing – original draft (equal); writing – review and editing (equal).

## CONFLICT OF INTEREST STATEMENT

The authors declare no conflicts of interest.

## Supporting information


Appendix S1

Data S1

Data S2

Data S3

Data S4

## Data Availability

All data used in this manuscript are listed in the manuscript and provided in the Data [Link], [Link].
