# Supplementary material for: Decline of Tephroseris helenitis in Hessia (Germany) over the last 120 years: Modeling implies the gradual disappearance of its temperature niche for flower induction and germination
Source: Ecol Evol. 2023 Dec 6;13(12):e10769. doi: 10.1002/ece3.10769 (PMC10700670; doi:10.1002/ece3.10769)

**Appendix S1**

**Table S1.** Climatic variables used for modelling the distribution of *T. helenitis*. Values are given at the spatial resolution of QTK25 (ca. 5.5 km x 5.5 km) and were calculated from 1 km x 1 km grid cells (Deutscher Wetterdienst, DWD) in QGIS Version 3.6 for four different periods (1900–1949, 1950–1979, 1980–1999 and 2000–2020) and the whole of Germany. Precipitation sums and mean temperatures are means across 1 km x 1 km cells and years of periods. Minimum and maximum temperatures are minimum and maximum values, respectively, across 1 km x 1 km cells and years of periods.

| Abbreviation | meaning |
| --- | --- |
| Precip_Jan | Precipitation sum of the month January (mm) |
| Precip_Feb | Precipitation sum of the month February (mm) |
| Precip_Mar | Precipitation sum of the month March (mm) |
| Precip_Apr | Precipitation sum of the month April (mm) |
| Precip_May | Precipitation sum of the month May (mm) |
| Precip_Jun | Precipitation sum of the month June (mm) |
| Precip_Jul | Precipitation sum of the month July (mm) |
| Precip_Aug | Precipitation sum of the month August (mm) |
| Precip_Sep | Precipitation sum of the month September (mm) |
| Precip_Oct | Precipitation sum of the month October (mm) |
| Precip_Nov | Precipitation sum of the month November (mm) |
| Precip_Dec | Precipitation sum of the month December (mm) |
| Precip_Year | Sum of Precip_Jan through Precip_Dec, annual precipitation sum (mm) |
|  |  |
| Tmean_Jan | Mean of air temperature of the month January (°C) |
| Tmean_Feb | Mean of air temperature of the month February (°C) |
| Tmean_Mar | Mean of air temperature of the month March (°C) |
| Tmean_Apr | Mean of air temperature of the month April (°C) |
| Tmean_May | Mean of air temperature of the month May (°C) |
| Tmean_Jun | Mean of air temperature of the month June (°C) |
| Tmean_Jul | Mean of air temperature of the month July (°C) |
| Tmean_Aug | Mean of air temperature of the month August (°C) |
| Tmean_Sep | Mean of air temperature of the month September (°C) |
| Tmean_Oct | Mean of air temperature of the month October (°C) |
| Tmean_Nov | Mean of air temperature of the month November (°C) |
| Tmean_Dec | Mean of air temperature of the month December (°C) |
| Tmean_Year | Mean of Tmean_Jan through Tmean_Dec, annual mean temperature (°C) |
|  |  |
| Tmax_Jan | Maximum of air temperature of the month January (°C) |
| Tmax_Feb | Maximum of air temperature of the month February (°C) |
| Tmax_Mar | Maximum of air temperature of the month March (°C) |
| Tmax_Apr | Maximum of air temperature of the month April (°C) |
| Tmax_May | Maximum of air temperature of the month May (°C) |
| Tmax_Jun | Maximum of air temperature of the month June (°C) |
| Tmax_Jul | Maximum of air temperature of the month July (°C) |
| Tmax_Aug | Maximum of air temperature of the month August (°C) |
| Tmax_Sep | Maximum of air temperature of the month September (°C) |
| Tmax_Oct | Maximum of air temperature of the month October (°C) |
| Tmax_Nov | Maximum of air temperature of the month November (°C) |
| Tmax_Dec | Maximum of air temperature of the month December (°C) |
|  |  |
| Tmin_Jan | Minimum of air temperature of the month January (°C) |
| Tmin_Feb | Minimum of air temperature of the month February (°C) |
| Tmin_Mar | Minimum of air temperature of the month March (°C) |
| Tmin_Apr | Minimum of air temperature of the month April (°C) |
| Tmin_May | Minimum of air temperature of the month May (°C) |
| Tmin_Jun | Minimum of air temperature of the month June (°C) |
| Tmin_Jul | Minimum of air temperature of the month July (°C) |
| Tmin_Aug | Minimum of air temperature of the month August (°C) |
| Tmin_Sep | Minimum of air temperature of the month September (°C) |
| Tmin_Oct | Minimum of air temperature of the month October (°C) |
| Tmin_Nov | Minimum of air temperature of the month November (°C) |
| Tmin_Dec | Minimum of air temperature of the month December (°C) |
|  |  |
| Tmax - Tmin_Jan | = Tmax_Jan – Tmin_Jan, air temperature range of the month January (°C) |
| Tmax - Tmin_Feb | = Tmax_Feb – Tmin_Feb, air temperature range of the month February (°C) |
| Tmax - Tmin_Mar | = Tmax_Mar – Tmin_Mar, air temperature range of the month March (°C) |
| Tmax - Tmin_Apr | = Tmax_Apr – Tmin_Apr, air temperature range of the month April (°C) |
| Tmax - Tmin_May | = Tmax_May – Tmin_May, air temperature range of the month May (°C) |
| Tmax - Tmin_Jun | = Tmax_Jun – Tmin_Jun, air temperature range of the month June (°C) |
| Tmax - Tmin_Jul | = Tmax_Jul – Tmin_Jul, air temperature range of the month July (°C) |
| Tmax - Tmin_Aug | = Tmax_Aug – Tmin_Aug, air temperature range of the month August (°C) |
| Tmax - Tmin_Sep | = Tmax_Sep – Tmin_Sep, air temperature range of the month September (°C) |
| Tmax - Tmin_Oct | = Tmax_Oct – Tmin_Oct, air temperature range of the month October (°C) |
| Tmax - Tmin_Nov | = Tmax_Nov – Tmin_Nov, air temperature range of the month November (°C) |
| Tmax - Tmin_Dec | = Tmax_Dec – Tmin_Dec, air temperature range of the month December (°C) |

**Figure S1.** Changes in climatic variables of QTK25s in Hessia between 1900–1949, 1950–1979, 1980–1999 and 2000–2020. A) Annual precipitation sum (Precip_Year), B) annual mean temperature (Tmean_Year). Hessia comprises 892 QTK25s. Letters (a,b,c) used in each boxplot indicate significant pairwise Wilcoxon posthoc tests after Bonferroni correction (p-value < 0.05) of the four periods. Medians Precip_Year: 1900–1949: 765.00 mm, 1950–1979: 799.00 mm, 1980–1999: 842.10 mm, 2000–2020: 782.90 mm; medians Tmean_Year: 1900–1949: 8.02°C, 1950–1979: 8.03°C, 1980–1999: 8.37°C, 2000–2020: 9.34°C.

| A) | B) |
| --- | --- |
| **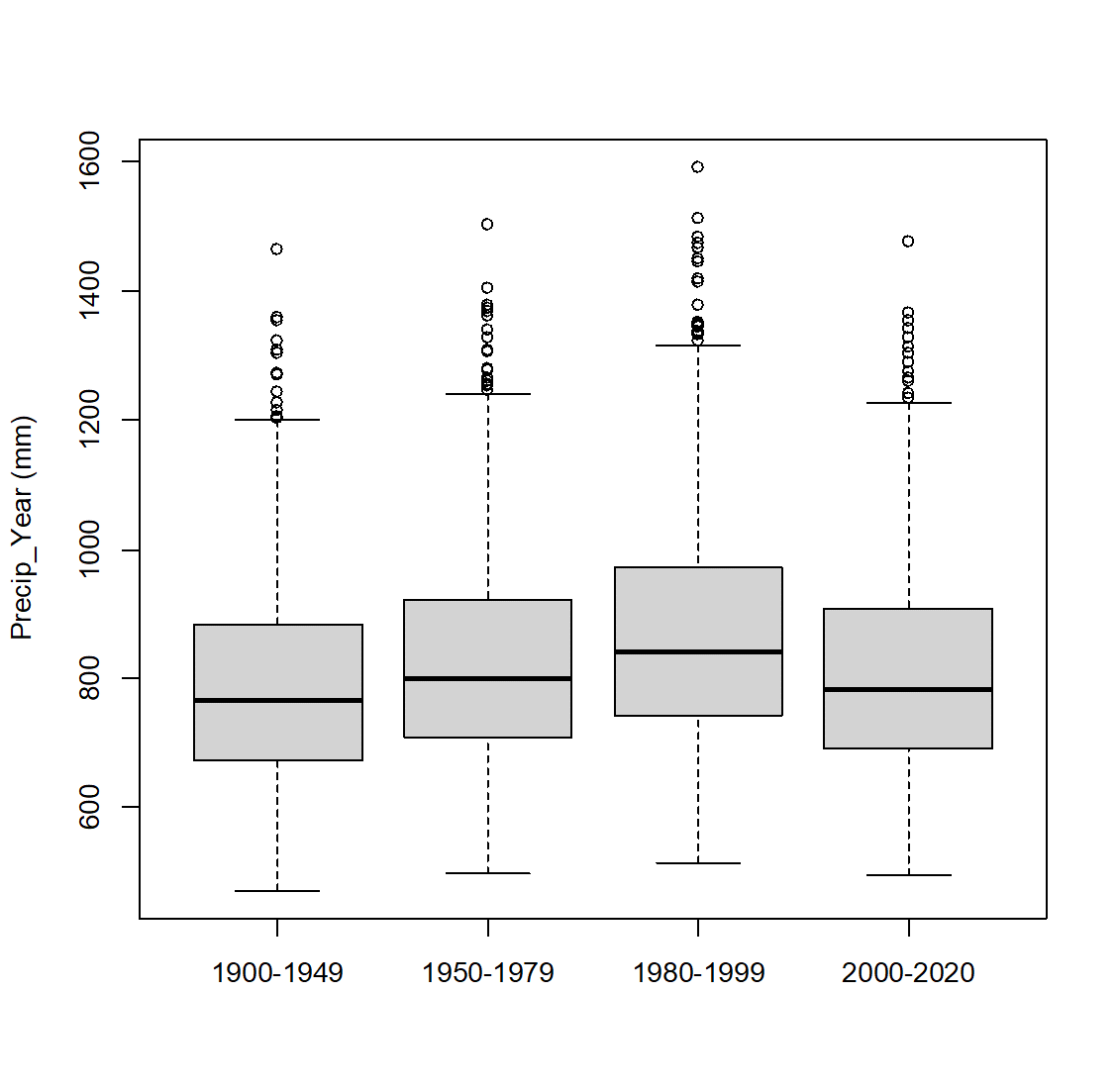** | **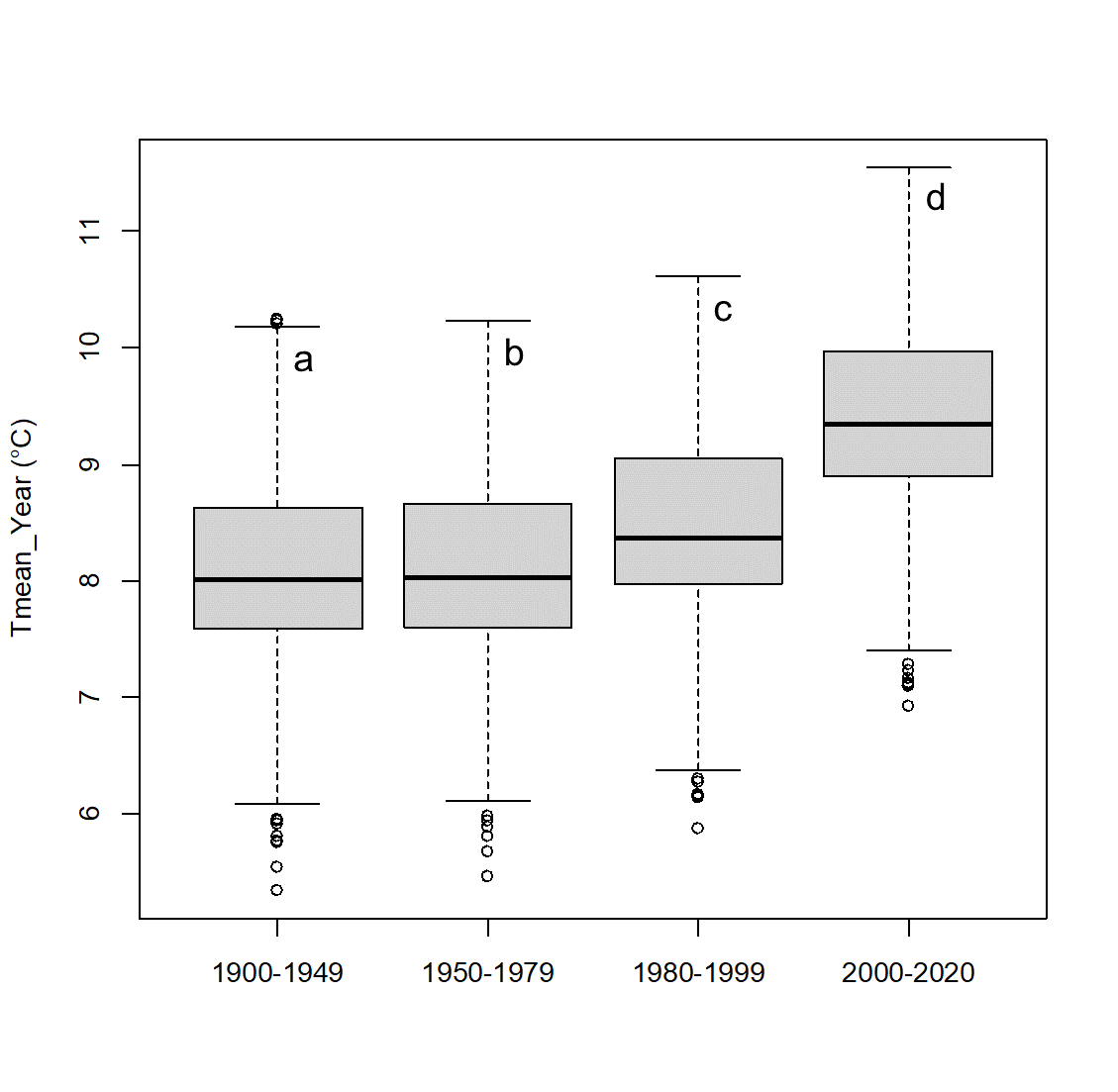** |

**Figure S2**. Spearman rank correlation analysis of the 62 climatic variables used for modelling *T. helenitis* in the study area (Table S1). A) 1900–1949, B) 1950–1979. White indicates non-significant correlation coefficients, blue indicates significant positive coefficients and red significant negative coefficients (for values of coefficients, see scale bars; Wei et al., 2018).

| A) | B) |
| --- | --- |
|  |  |

**Figure S3**. Changes in climatic variables of QTK25s in the study area between 1900–1949, 1950–1979, 1980–1999 and 2000–2020. A) Monthly precipitation sums, B) monthly mean temperatures, C) monthly maximum temperatures, D) monthly minimum temperatures, E) monthly temperature ranges (maximum – minimum temperature), F) annual precipitation sum and annual mean temperature. all: across the study area of *T. helenitis*, defined as the 55 QTK25 with records from before 1850 and 1850-1899 (Kadereit et al., 2021), abs: only absences, pre: only presences. Letters (a,b,c) in boxplots indicate significant differences in medians of climatic variables (pairwise Wilcoxon posthoc tests with Bonferroni correction). Greek letters: differences between 1900–1949, 1950–1979, 1980–1999 and 2000–2020 (Table S2), capital letters: presences vs. absences for 1900–1949 (Table S3), capital letters in italics: presences vs. absences for 1950–1979 (Table S4), lowercase letters: absences in 1900–1949 vs. absences in 1950–1979 (Table S10), lowercase letters in italics: presences in 1900–1949 vs. presences in 1950–1979 (Table S10).

A)


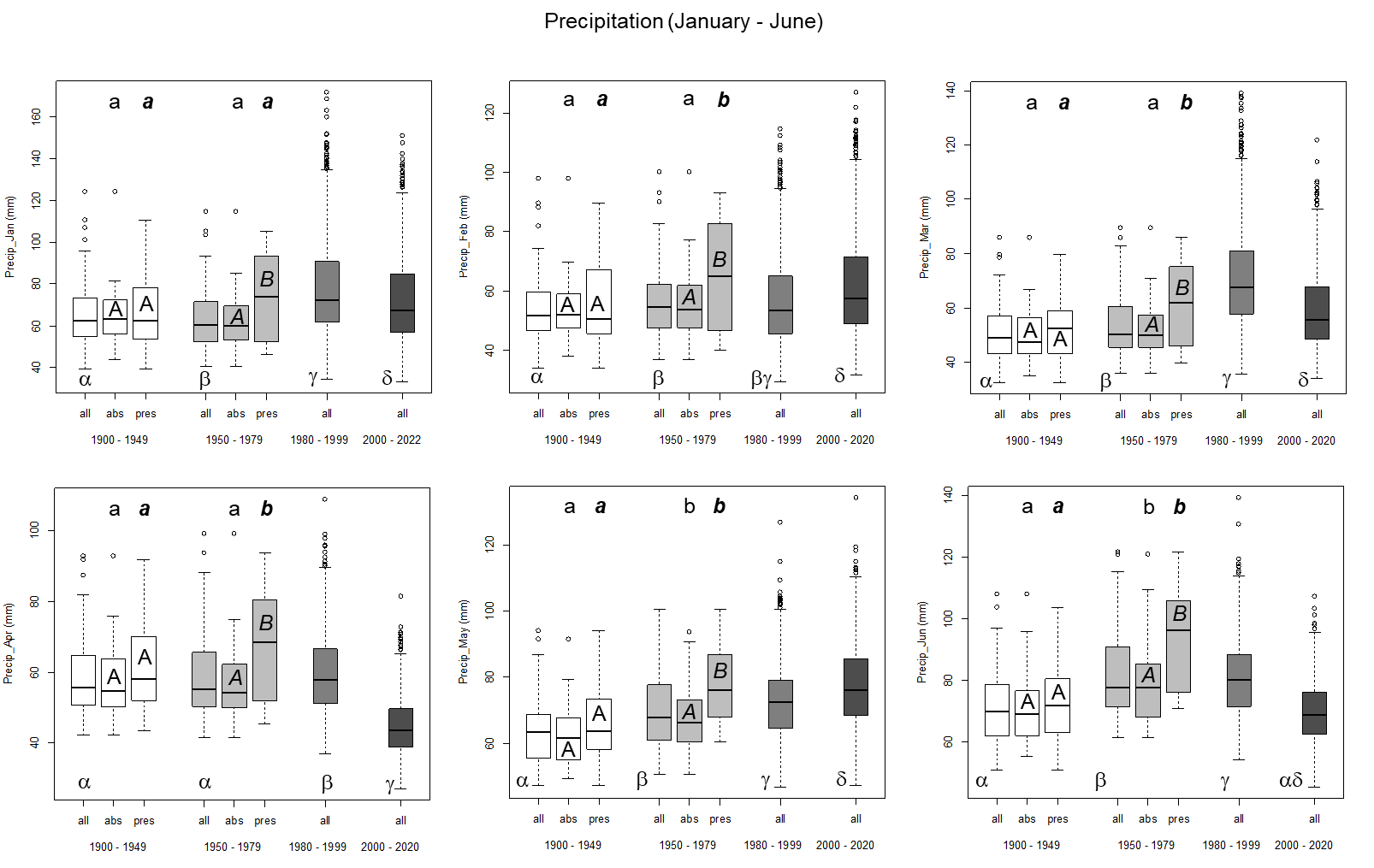


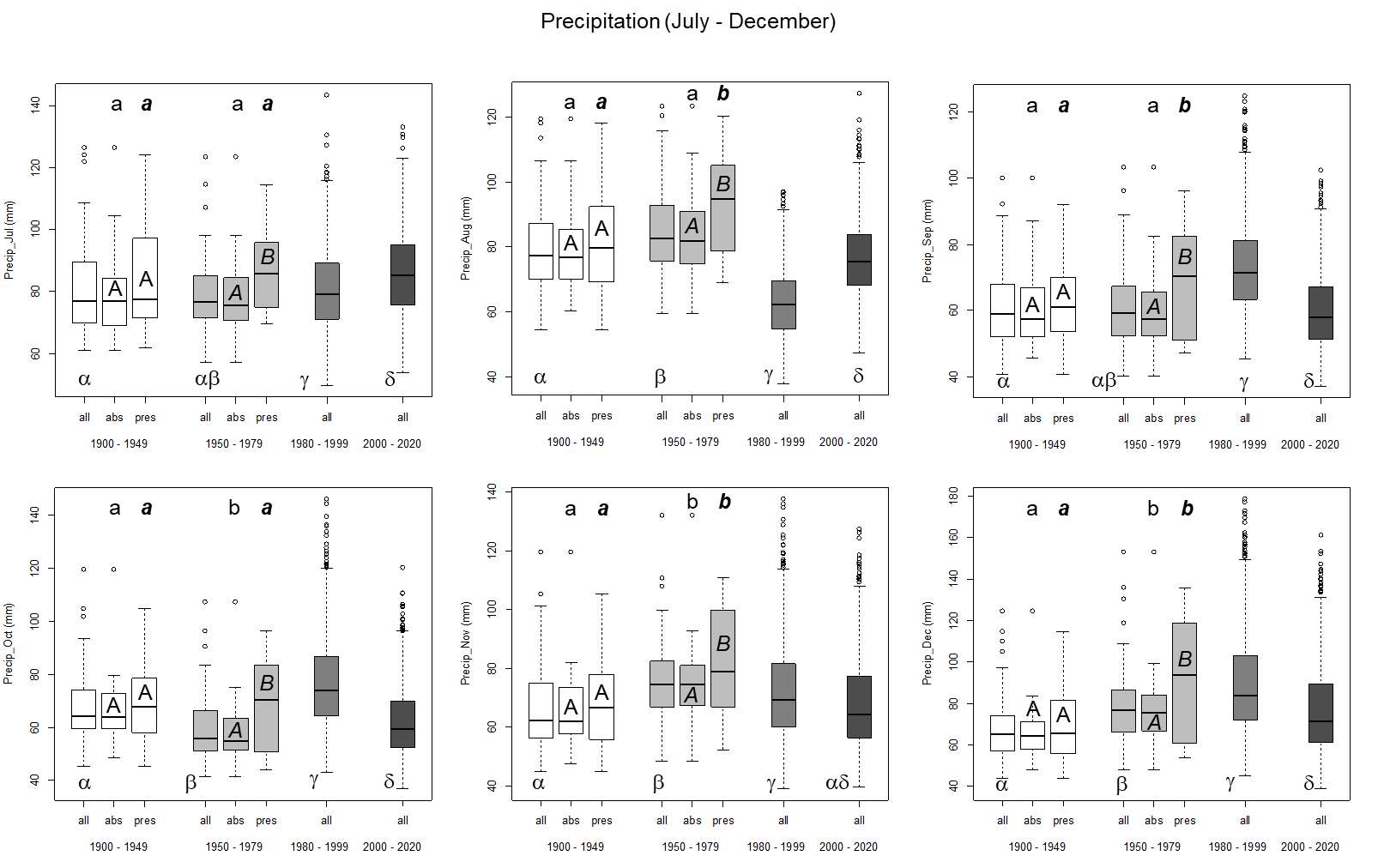


B)


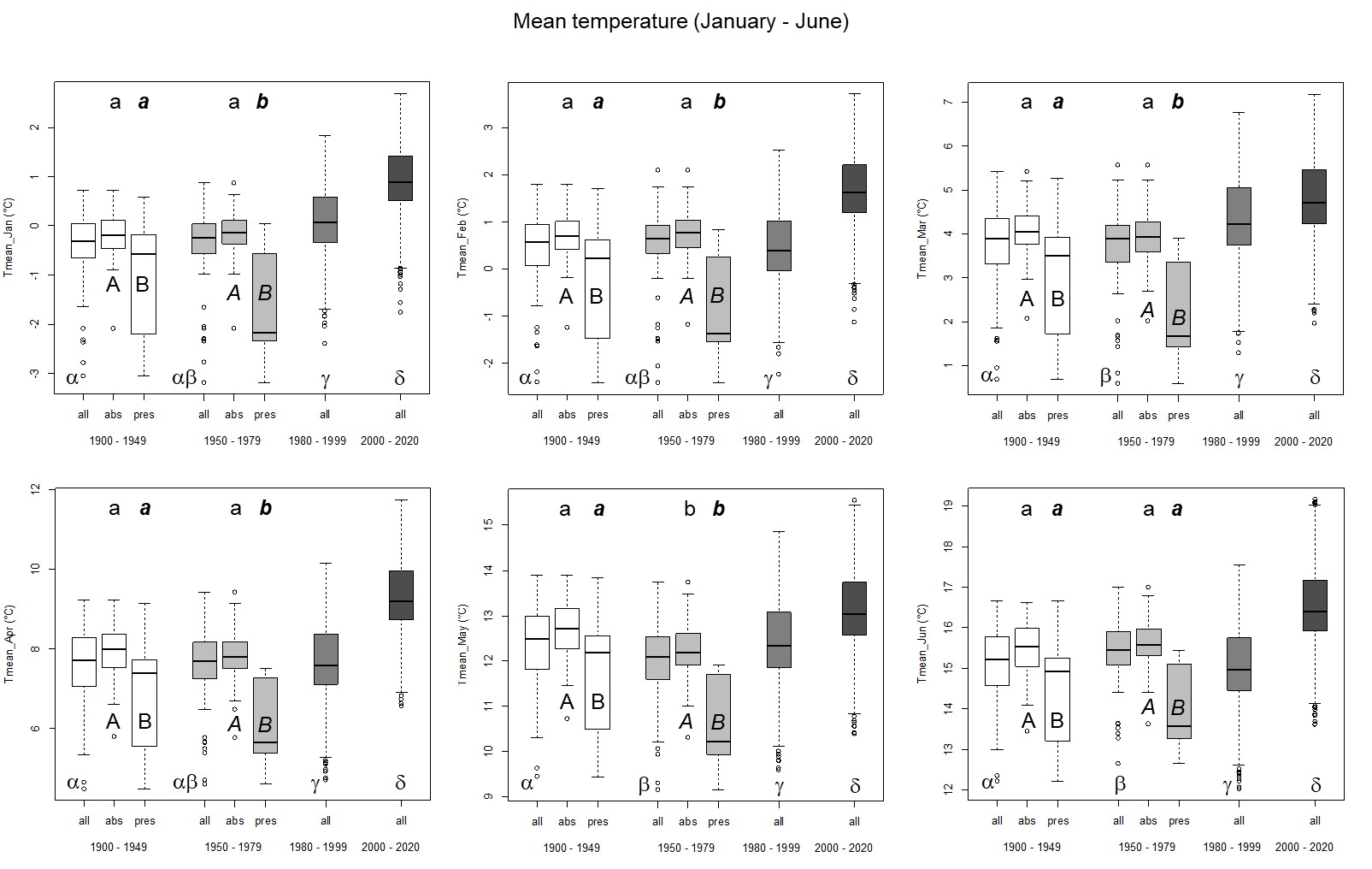


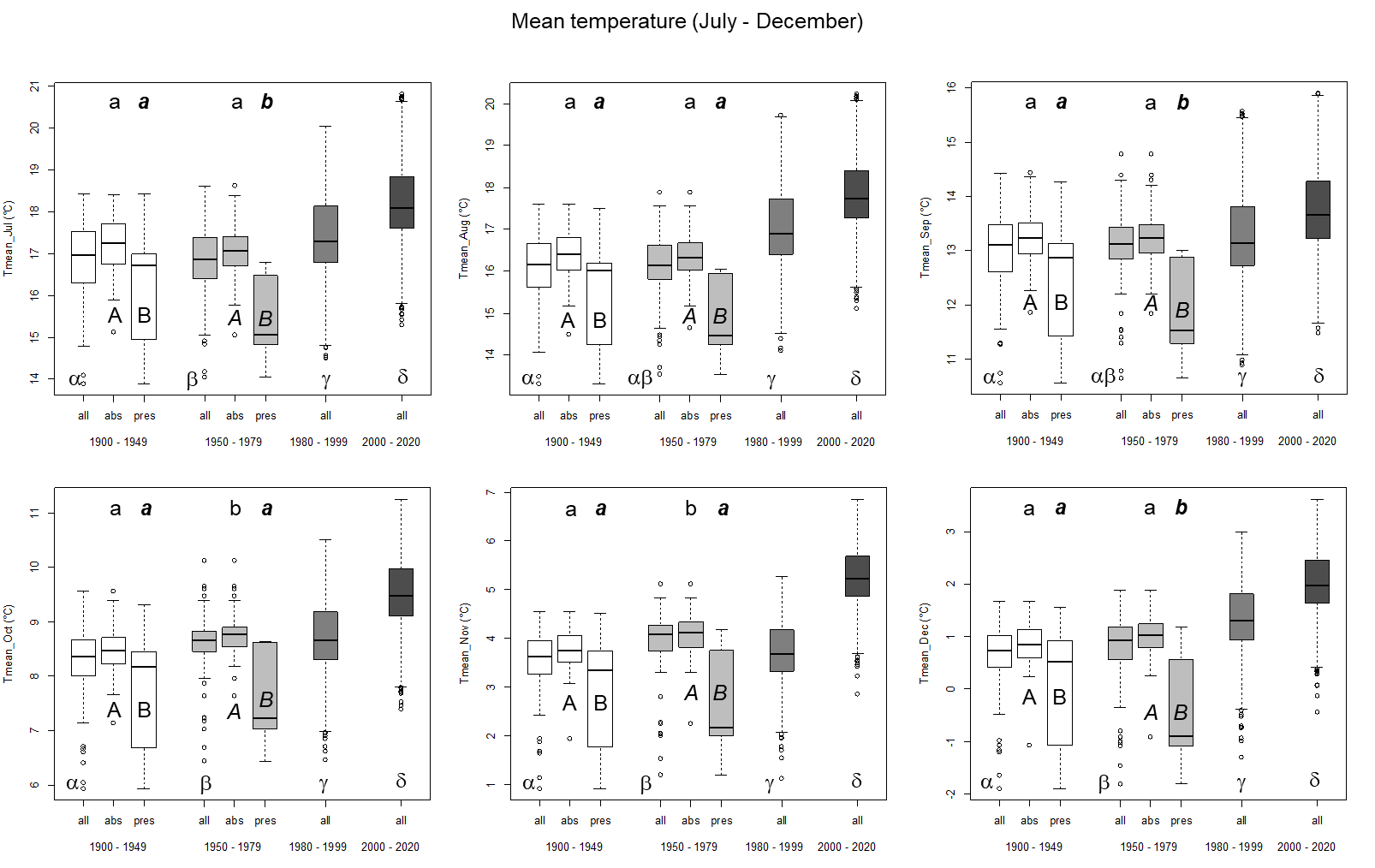


C)


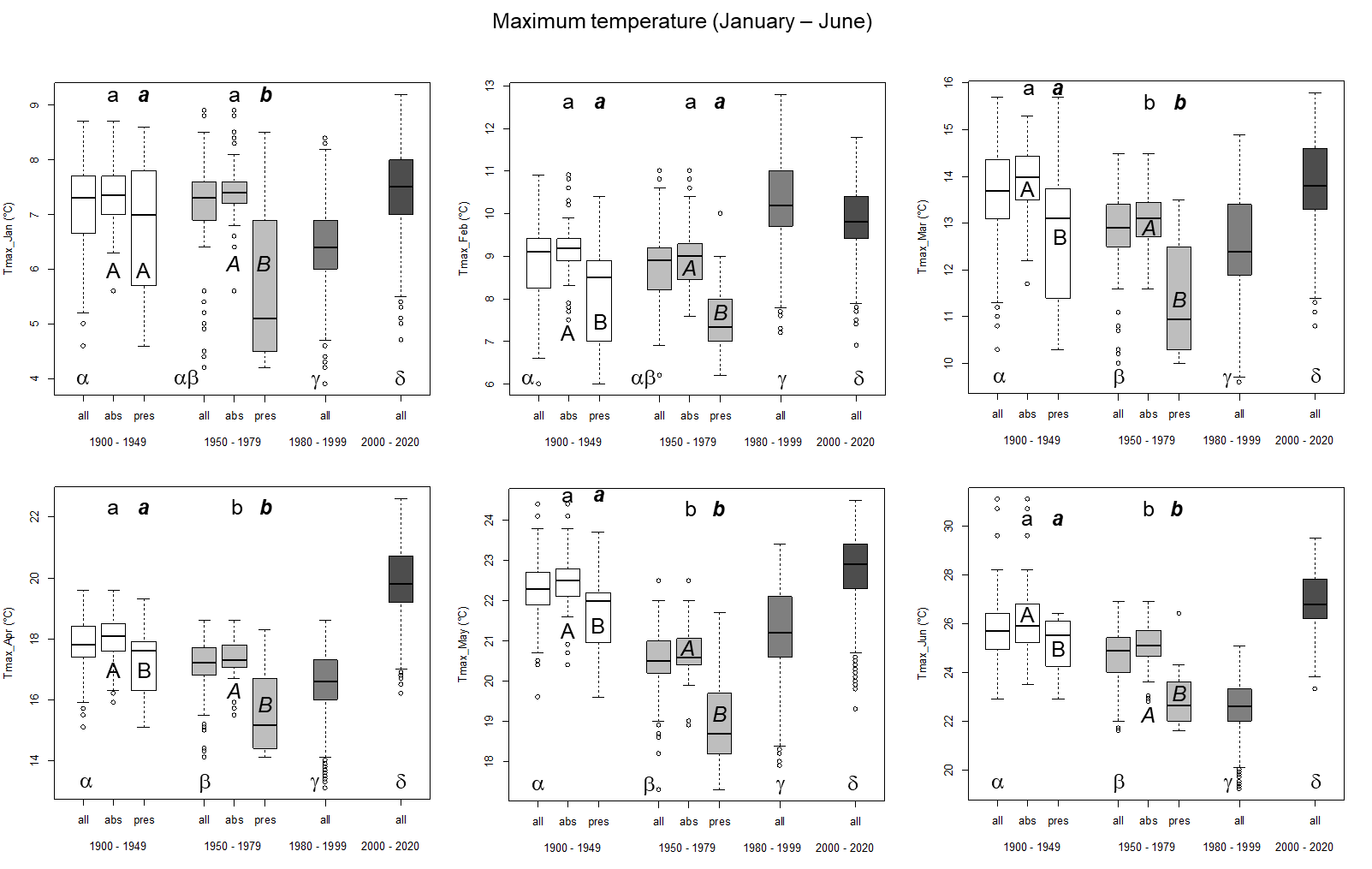


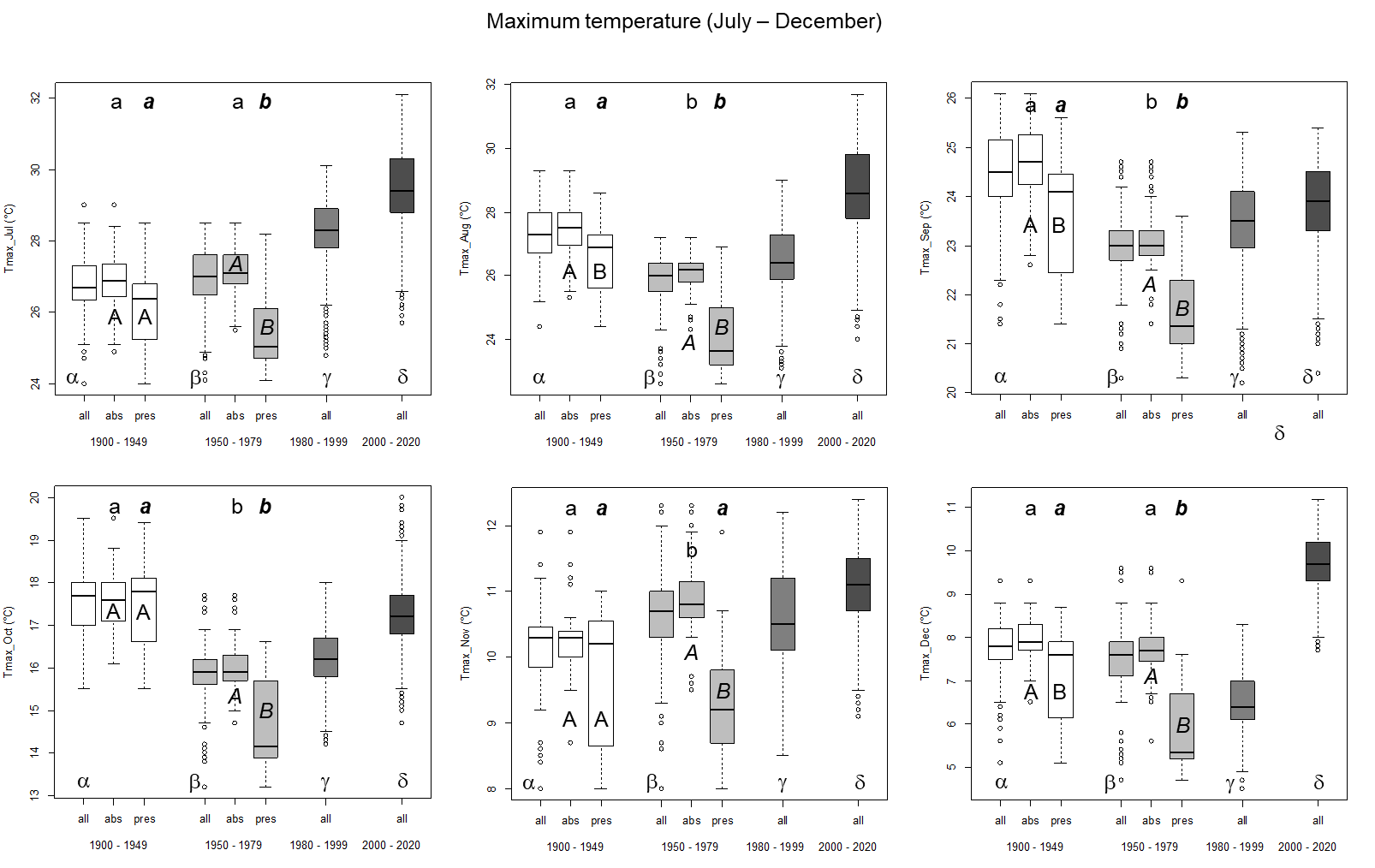


D)

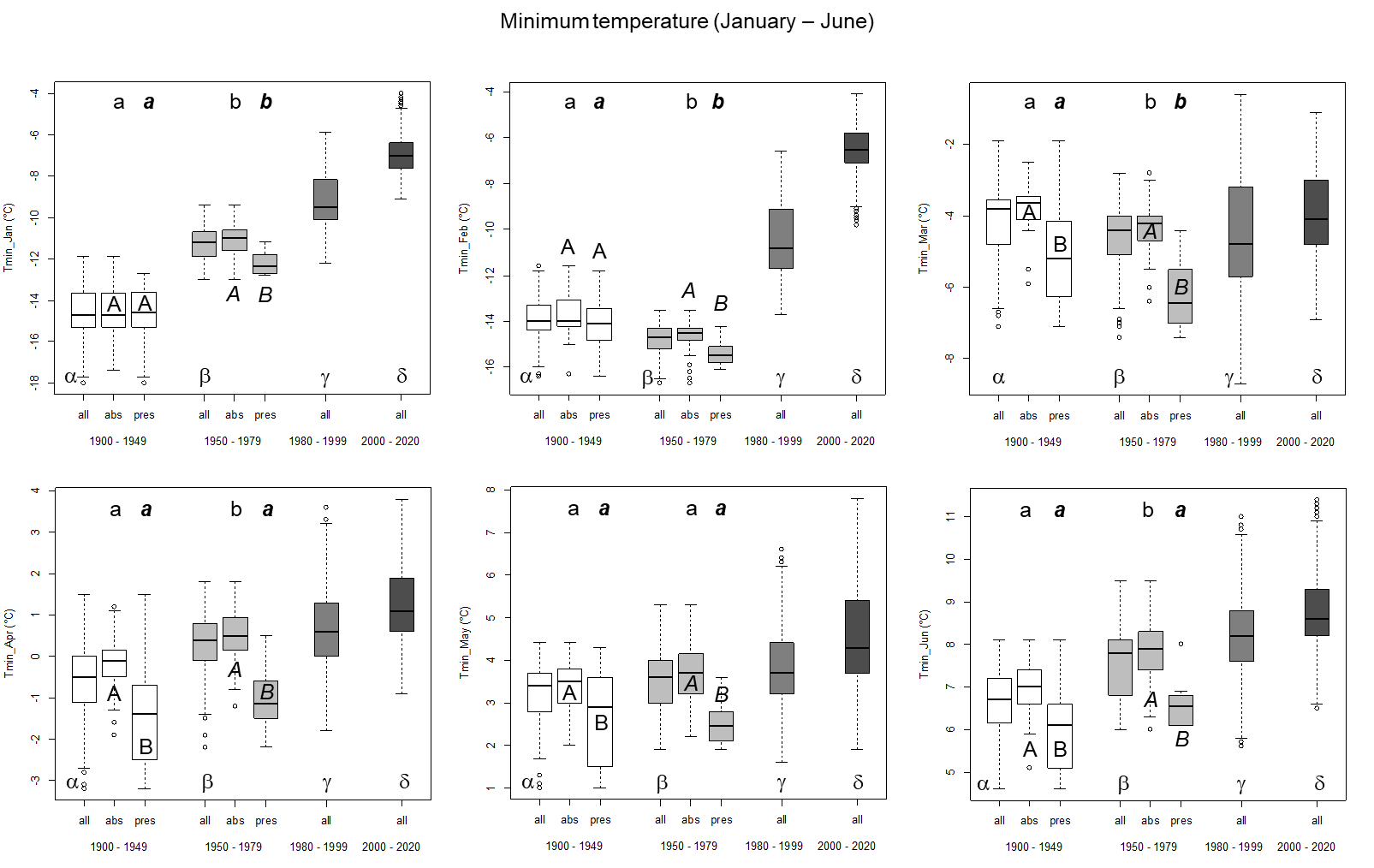


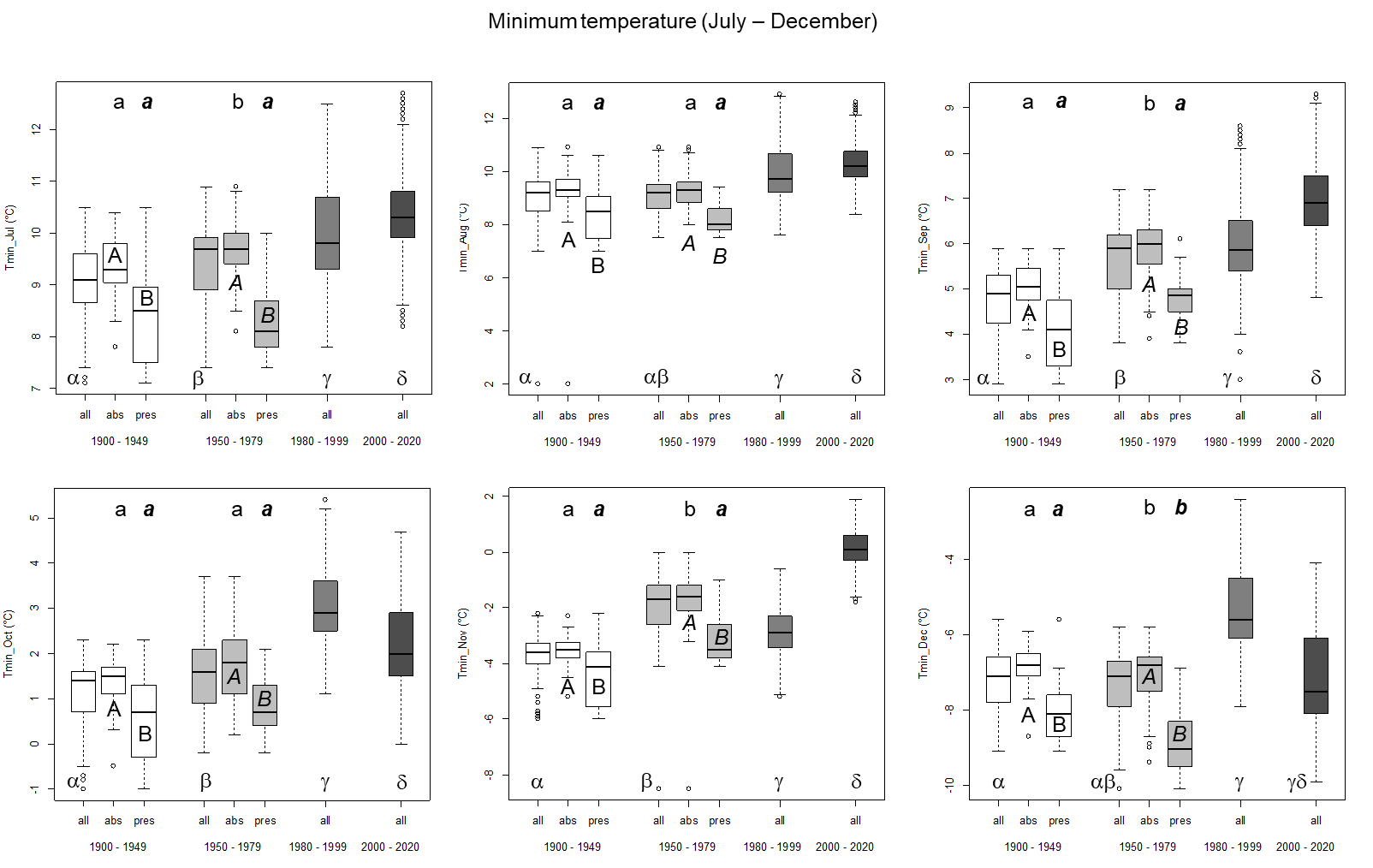


E)


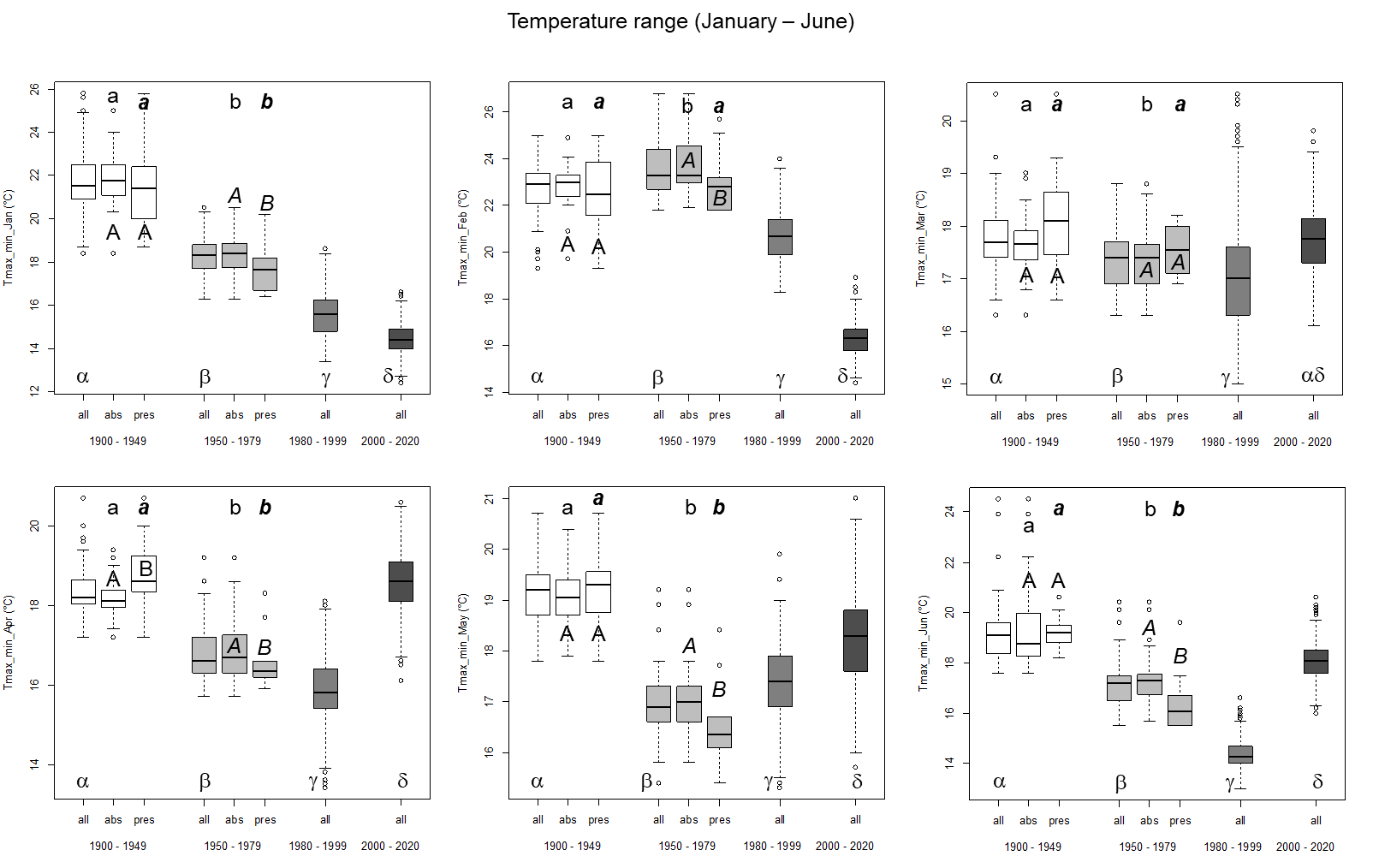


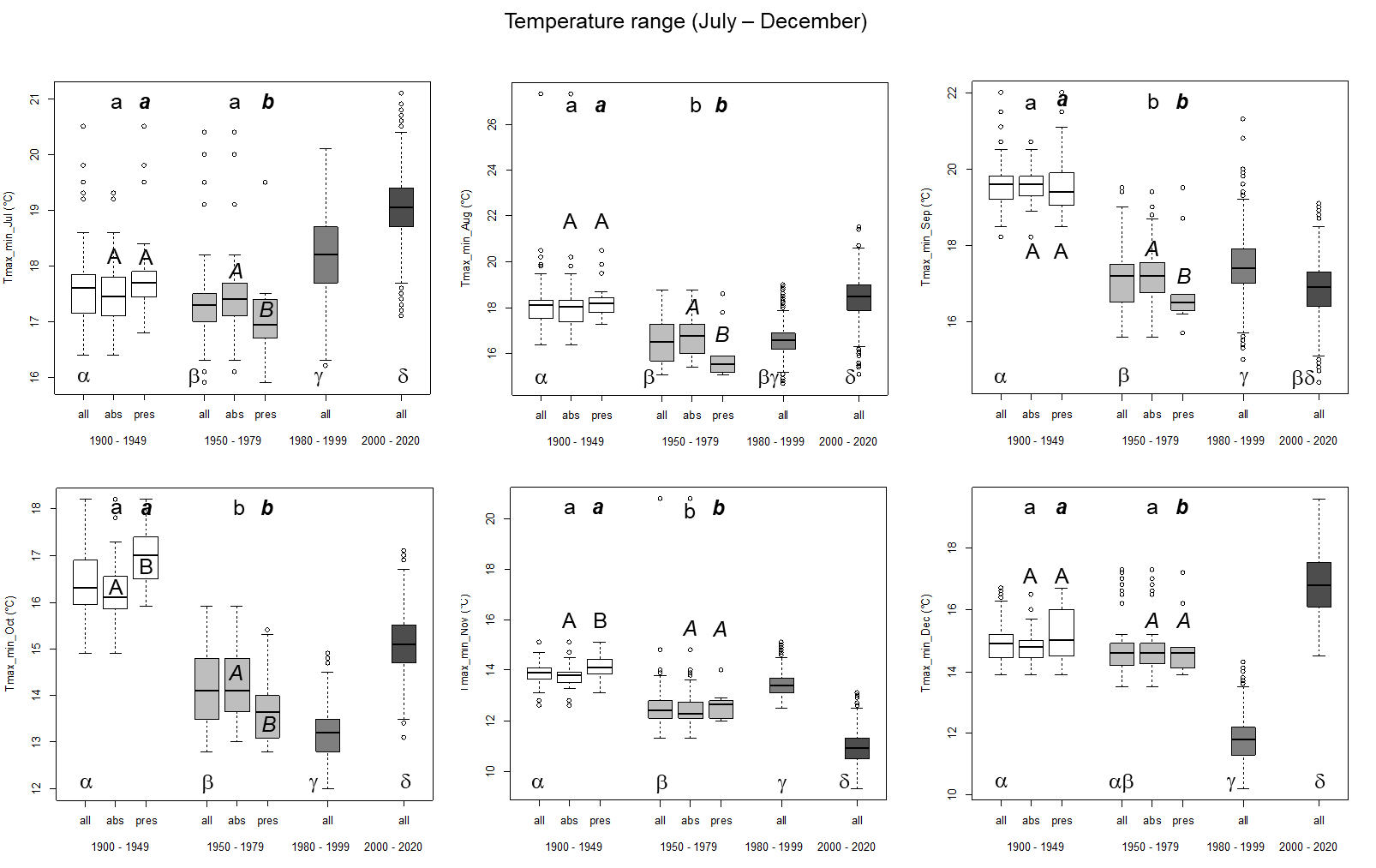


F)


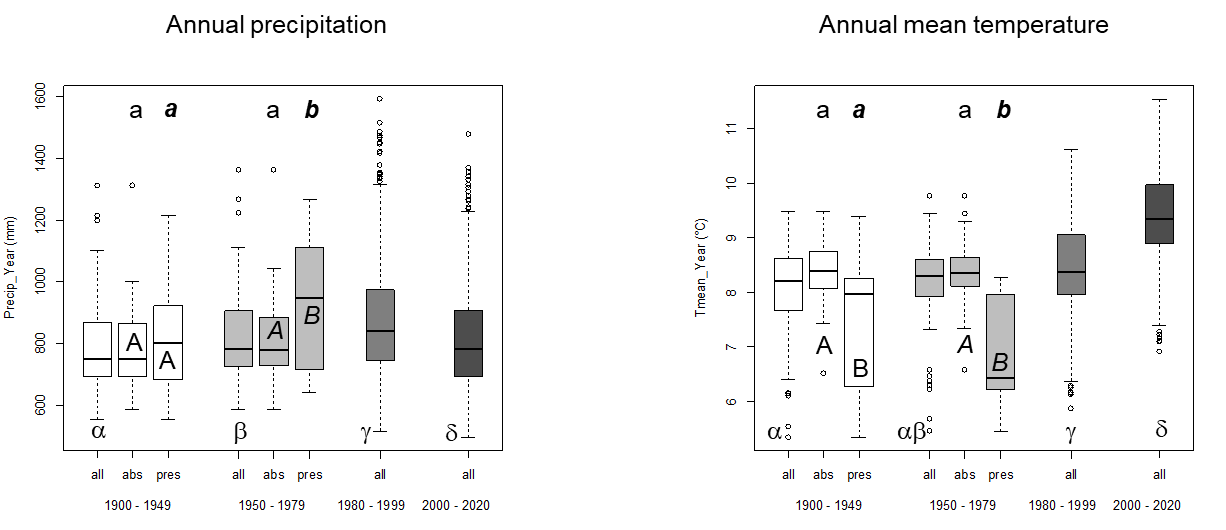


**Table S2**. Changes in climatic variables of QTK25s over three periods in the study area of *T. helenitis*. A) m_1_ = median for 1900–1949, m_2_ = median for 1950–1979, m_3_ = median for 1980–1999, m_4_ = median for 2000–2020; min_1_, max_1_, min_2_, max_3_, min_3_, max_3_, min_4_ and max_4_ = minimum and maximum of the periods. B) m_i_-m_j_ = difference between m_i_ and m_j_; Chi^2^, df, p_Friedman_ = results of Friedman tests (non-parametric ANOVA for paired samples) of median differences across periods; asterisks give significance levels of pairwise Wilcoxon posthoc tests after Bonferroni correction; significance levels: red p > 0.05, * p ≤ 0.05, ** p ≤ 0.01, *** p ≤ 0.001. For graphical representation see Figure S3.

A)

| Predictor | m_1_ | min_1_ | max_1_ | m_2_ | min_2_ | max_2_ | m_3_ | min_3_ | max_3_ | m_4_ | min_4_ | max_4_ |
| --- | --- | --- | --- | --- | --- | --- | --- | --- | --- | --- | --- | --- |
| Precip_Jan | 61.86 | 39.47 | 124.34 | 60.55 | 40.59 | 114.76 | 72.32 | 34.38 | 171.89 | 64.71 | 49.75 | 147.69 |
| Precip_Feb | 51.55 | 34.00 | 97.80 | 53.75 | 36.97 | 100.15 | 53.59 | 29.40 | 114.53 | 57.21 | 33.21 | 92.36 |
| Precip_Mar | 48.29 | 32.42 | 85.84 | 50.38 | 35.71 | 89.40 | 67.70 | 35.46 | 139.15 | 54.25 | 43.51 | 124.32 |
| Precip_Apr | 55.02 | 41.97 | 92.83 | 55.16 | 41.34 | 99.05 | 57.66 | 36.93 | 108.77 | 44.09 | 42.02 | 93.80 |
| Precip_May | 62.33 | 47.09 | 93.91 | 67.70 | 50.65 | 100.31 | 72.33 | 46.61 | 126.76 | 74.37 | 54.54 | 105.62 |
| Precip_Jun | 69.19 | 50.94 | 107.90 | 77.68 | 61.39 | 121.70 | 79.99 | 53.99 | 139.33 | 67.78 | 62.20 | 119.35 |
| Precip_Jul | 76.77 | 61.01 | 126.29 | 76.64 | 57.05 | 123.48 | 78.91 | 49.58 | 143.29 | 81.25 | 59.78 | 130.36 |
| Precip_Aug | 77.00 | 54.24 | 119.51 | 82.66 | 59.46 | 123.36 | 62.12 | 37.74 | 96.89 | 74.15 | 44.77 | 92.46 |
| Precip_Sep | 58.46 | 40.64 | 100.01 | 58.51 | 40.20 | 103.36 | 71.45 | 45.41 | 124.84 | 54.35 | 53.95 | 120.12 |
| Precip_Oct | 63.59 | 45.07 | 119.60 | 55.54 | 41.39 | 107.40 | 73.95 | 42.90 | 146.25 | 56.74 | 47.87 | 139.60 |
| Precip_Nov | 61.71 | 44.89 | 119.47 | 73.38 | 48.36 | 132.18 | 69.03 | 38.94 | 137.64 | 63.02 | 48.54 | 124.21 |
| Precip_Dec | 62.93 | 44.13 | 124.41 | 75.83 | 48.12 | 152.99 | 83.77 | 45.10 | 178.59 | 73.67 | 54.27 | 167.14 |
| Precip_Year | 749.90 | 554.80 | 1309.40 | 782.20 | 587.90 | 1360.60 | 842.10 | 515.80 | 1590.30 | 768.20 | 613.30 | 1448.90 |
|  |  |  |  |  |  |  |  |  |  |  |  |  |
| Tmean_Jan | -0.40 | -3.05 | 0.73 | -0.24 | -3.19 | 0.87 | 0.06 | -2.40 | 1.84 | 1.11 | -2.40 | 1.60 |
| Tmean_Feb | 0.60 | -2.41 | 1.81 | 0.65 | -2.42 | 2.10 | 0.40 | -2.24 | 2.53 | 1.88 | -2.24 | 2.37 |
| Tmean_Mar | 4.01 | 0.69 | 5.41 | 3.89 | 0.59 | 5.56 | 4.23 | 1.29 | 6.76 | 5.12 | 1.29 | 6.26 |
| Tmean_Apr | 7.80 | 4.46 | 9.23 | 7.74 | 4.58 | 9.42 | 7.59 | 4.70 | 10.18 | 9.51 | 4.91 | 9.46 |
| Tmean_May | 12.39 | 9.44 | 13.90 | 12.10 | 9.15 | 13.75 | 12.34 | 9.58 | 14.87 | 13.28 | 9.78 | 14.12 |
| Tmean_Jun | 15.30 | 12.21 | 16.66 | 15.46 | 12.64 | 17.00 | 14.97 | 12.02 | 17.56 | 16.70 | 12.35 | 16.68 |
| Tmean_Jul | 17.09 | 13.89 | 18.42 | 16.80 | 14.05 | 18.62 | 17.30 | 14.51 | 20.03 | 18.36 | 14.76 | 19.33 |
| Tmean_Aug | 16.32 | 13.31 | 17.60 | 16.15 | 13.54 | 17.88 | 16.90 | 14.11 | 19.72 | 17.98 | 14.61 | 19.08 |
| Tmean_Sep | 13.03 | 10.56 | 14.43 | 13.14 | 10.65 | 14.78 | 13.13 | 10.89 | 15.56 | 13.91 | 10.97 | 15.21 |
| Tmean_Oct | 8.43 | 5.93 | 9.56 | 8.73 | 6.44 | 10.13 | 8.66 | 6.46 | 10.51 | 9.75 | 6.46 | 10.51 |
| Tmean_Nov | 3.70 | 0.91 | 4.55 | 4.10 | 1.20 | 5.12 | 3.68 | 1.13 | 5.27 | 5.43 | 1.13 | 5.04 |
| Tmean_Dec | 0.82 | -1.90 | 1.66 | 0.93 | -1.81 | 1.88 | 1.32 | -1.31 | 2.99 | 2.18 | -1.31 | 2.74 |
| Tmean_Year | 8.29 | 5.34 | 9.48 | 8.30 | 5.45 | 9.76 | 8.37 | 5.86 | 10.63 | 9.60 | 5.86 | 10.20 |
|  |  |  |  |  |  |  |  |  |  |  |  |  |
| Tmax_Jan | 7.40 | 4.60 | 8.70 | 7.40 | 4.20 | 8.90 | 6.40 | 3.90 | 8.40 | 7.80 | 4.70 | 9.00 |
| Tmax_Feb | 9.10 | 6.00 | 10.90 | 9.00 | 6.20 | 11.00 | 10.20 | 7.20 | 12.80 | 10.00 | 6.90 | 11.60 |
| Tmax_Mar | 13.90 | 10.30 | 15.70 | 12.90 | 10.00 | 14.50 | 12.40 | 9.60 | 14.90 | 14.00 | 11.30 | 15.60 |
| Tmax_Apr | 17.90 | 15.10 | 19.60 | 17.20 | 14.10 | 18.60 | 16.60 | 13.10 | 18.60 | 19.90 | 17.60 | 22.20 |
| Tmax_May | 22.30 | 19.60 | 24.40 | 20.50 | 17.30 | 22.50 | 21.20 | 17.90 | 23.40 | 23.00 | 20.60 | 24.10 |
| Tmax_Jun | 25.70 | 22.90 | 31.10 | 25.00 | 21.60 | 26.90 | 22.60 | 19.20 | 25.10 | 27.00 | 24.50 | 29.50 |
| Tmax_Jul | 26.80 | 24.00 | 29.00 | 27.00 | 24.10 | 28.50 | 28.30 | 24.80 | 30.10 | 29.80 | 26.90 | 31.50 |
| Tmax_Aug | 27.30 | 24.40 | 29.30 | 26.00 | 22.60 | 27.20 | 26.40 | 23.10 | 29.00 | 28.90 | 26.40 | 31.50 |
| Tmax_Sep | 24.50 | 21.40 | 26.10 | 23.00 | 20.30 | 24.70 | 23.50 | 20.20 | 25.30 | 24.20 | 21.30 | 25.30 |
| Tmax_Oct | 17.80 | 15.50 | 19.50 | 15.90 | 13.20 | 17.70 | 16.20 | 14.20 | 18.00 | 17.40 | 15.00 | 19.20 |
| Tmax_Nov | 10.30 | 8.00 | 11.90 | 10.80 | 8.00 | 12.30 | 10.50 | 8.50 | 12.20 | 11.10 | 9.50 | 12.40 |
| Tmax_Dec | 7.90 | 5.10 | 9.30 | 7.60 | 4.70 | 9.60 | 6.40 | 4.50 | 8.30 | 9.80 | 7.70 | 10.90 |
|  |  |  |  |  |  |  |  |  |  |  |  |  |
| Tmin_Jan | -14.70 | -18.00 | -11.90 | -11.20 | -13.00 | -9.40 | -9.50 | -12.20 | -5.90 | -6.80 | -11.50 | -7.20 |
| Tmin_Feb | -14.00 | -16.40 | -11.60 | -14.60 | -16.70 | -13.50 | -10.80 | -13.70 | -6.60 | -6.10 | -13.00 | -7.80 |
| Tmin_Mar | -3.80 | -7.10 | -1.90 | -4.40 | -7.40 | -2.80 | -4.80 | -8.70 | -0.60 | -3.40 | -8.10 | -2.10 |
| Tmin_Apr | -0.30 | -3.20 | 1.50 | 0.40 | -2.20 | 1.80 | 0.60 | -1.80 | 3.60 | 1.10 | -1.40 | 2.10 |
| Tmin_May | 3.50 | 1.00 | 4.40 | 3.60 | 1.90 | 5.30 | 3.70 | 1.60 | 6.60 | 5.00 | 1.90 | 5.10 |
| Tmin_Jun | 6.80 | 4.60 | 8.10 | 7.80 | 6.00 | 9.50 | 8.20 | 5.60 | 11.00 | 8.90 | 5.90 | 9.70 |
| Tmin_Jul | 9.20 | 7.10 | 10.50 | 9.70 | 7.40 | 10.90 | 9.80 | 7.80 | 12.50 | 10.30 | 8.20 | 11.50 |
| Tmin_Aug | 9.20 | 2.00 | 10.90 | 9.20 | 7.50 | 10.90 | 9.70 | 7.60 | 12.90 | 10.30 | 8.40 | 11.70 |
| Tmin_Sep | 4.90 | 2.90 | 5.90 | 6.00 | 3.70 | 7.20 | 5.85 | 3.00 | 8.60 | 7.00 | 4.50 | 7.50 |
| Tmin_Oct | 1.40 | -1.00 | 2.30 | 1.70 | -0.20 | 3.70 | 2.90 | 1.10 | 5.40 | 2.40 | 1.10 | 4.60 |
| Tmin_Nov | -3.60 | -6.00 | -2.20 | -1.70 | -8.50 | 0.00 | -2.90 | -5.20 | -0.60 | 0.10 | -5.20 | -1.50 |
| Tmin_Dec | -7.00 | -9.10 | -5.60 | -7.00 | -10.10 | -5.80 | -5.60 | -7.90 | -2.40 | -6.60 | -7.40 | -3.60 |
|  |  |  |  |  |  |  |  |  |  |  |  |  |
| Tmax - Tmin_Jan | 21.50 | 18.40 | 25.80 | 18.40 | 16.30 | 20.50 | 15.20 | 13.90 | 18.40 | 14.50 | 13.90 | 18.40 |
| Tmax - Tmin_Feb | 22.90 | 19.30 | 25.00 | 23.30 | 21.80 | 26.80 | 20.10 | 18.80 | 23.40 | 16.00 | 18.80 | 23.40 |
| Tmax - Tmin_Mar | 17.70 | 16.30 | 20.50 | 17.40 | 16.30 | 18.80 | 16.80 | 15.80 | 20.50 | 17.70 | 15.80 | 20.50 |
| Tmax - Tmin_Apr | 18.20 | 17.20 | 20.70 | 16.70 | 15.70 | 19.20 | 15.90 | 14.00 | 18.00 | 18.80 | 14.00 | 18.00 |
| Tmax - Tmin_May | 19.10 | 17.80 | 20.70 | 16.90 | 15.40 | 19.20 | 17.70 | 15.60 | 19.00 | 18.20 | 15.60 | 19.00 |
| Tmax - Tmin_Jun | 18.90 | 17.60 | 24.50 | 17.30 | 15.50 | 20.40 | 14.50 | 13.40 | 16.60 | 18.20 | 13.40 | 16.60 |
| Tmax - Tmin_Jul | 17.50 | 16.40 | 20.50 | 17.30 | 15.90 | 20.40 | 18.20 | 17.20 | 20.10 | 19.40 | 17.20 | 20.10 |
| Tmax - Tmin_Aug | 18.10 | 16.40 | 27.30 | 16.70 | 15.10 | 18.80 | 16.70 | 15.30 | 19.00 | 18.60 | 15.30 | 19.00 |
| Tmax - Tmin_Sep | 19.60 | 18.20 | 22.00 | 17.20 | 15.60 | 19.50 | 17.50 | 15.70 | 19.40 | 17.10 | 15.70 | 19.40 |
| Tmax - Tmin_Oct | 16.30 | 14.90 | 18.20 | 14.10 | 12.80 | 15.90 | 13.30 | 12.20 | 14.80 | 15.10 | 12.20 | 14.80 |
| Tmax - Tmin_Nov | 13.90 | 12.60 | 15.10 | 12.40 | 11.30 | 20.80 | 13.50 | 12.70 | 15.10 | 10.80 | 12.70 | 15.10 |
| Tmax - Tmin_Dec | 14.90 | 13.90 | 16.70 | 14.60 | 13.50 | 17.40 | 11.80 | 10.80 | 13.80 | 16.50 | 10.80 | 13.80 |

B)

| Predictor | m_2_ - m_1_ | m_3_ - m_1_ | m_4_ – m_1_ | m_3_ - m_2_ | m_4_ - m_2_ | m_4_ - m_3_ | Chi^2^ | df | p_Friedman_ |
| --- | --- | --- | --- | --- | --- | --- | --- | --- | --- |
| Precip_Jan | -1.31*** | 10.46*** | 2.85*** | 11.77*** | 4.16*** | -7.61*** | 2169.6 | 3 | < 2.2e-16 |
| Precip_Feb | 2.20*** | 2.04*** | 5.66*** | 0.16 | 3.46*** | 3.62*** | 1321.9 | 3 | < 2.2e-16 |
| Precip_Mar | 2.09*** | 19.41*** | 5.96*** | 17.32*** | 3.87*** | -13.45*** | 2582.5 | 3 | < 2.2e-16 |
| Precip_Apr | 0.14 | 2.64*** | -10.93*** | 2.50* | -11.07*** | -13.57*** | 1620.2 | 3 | < 2.2e-16 |
| Precip_May | 5.37*** | 10.00*** | 12.04*** | 4.63*** | 6.67*** | 2.04*** | 2063.4 | 3 | < 2.2e-16 |
| Precip_Jun | 8.49*** | 10.80*** | -1.41 | 2.31*** | -9.90*** | -12.21*** | 2143.3 | 3 | < 2.2e-16 |
| Precip_Jul | -0.13 | -2.14*** | 4.48*** | 2.27* | 4.61*** | 2.34*** | 430.9 | 3 | < 2.2e-16 |
| Precip_Aug | 5.66*** | -14.88*** | -2.85*** | -20.54*** | -8.51*** | 12.03*** | 2285.4 | 3 | < 2.2e-16 |
| Precip_Sep | 0.05 | 12.99*** | -4.11** | 12.94*** | -4.16*** | -17.10*** | 1737.1 | 3 | < 2.2e-16 |
| Precip_Oct | -8.05*** | 10.36*** | -6.85*** | 10.36*** | 1.20*** | -17.21*** | 2267.6 | 3 | < 2.2e-16 |
| Precip_Nov | 11.67*** | 7.32*** | 1.31 | -4.35*** | -10.36*** | -6.01*** | 1381.0 | 3 | < 2.2e-16 |
| Precip_Dec | 12.90*** | 20.84*** | 10.74*** | 7.94*** | -1.71* | -10.10*** | 1315.3 | 3 | < 2.2e-16 |
| Precip_Year | 32.30*** | 92.20*** | 18.30*** | 59.90*** | -14.00*** | -73.90*** | 2128.0 | 3 | < 2.2e-16 |
|  |  |  |  |  |  |  |  |  |  |
| Tmean_Jan | 0.16 | 0.46*** | 1.51*** | 0.30*** | 1.35*** | 1.51*** | 2412.6 | 3 | < 2.2e-16 |
| Tmean_Feb | 0.05 | -0.20*** | 1.28*** | -0.25*** | 1.23*** | 1.48*** | 1842.9 | 3 | < 2.2e-16 |
| Tmean_Mar | -0.12*** | 0.22*** | 1.11*** | 0.34*** | 1.23*** | 0.89*** | 2529.5 | 3 | < 2.2e-16 |
| Tmean_Apr | -0.06 | -0.21*** | 1.71*** | -0.15*** | 1.77*** | 1.92*** | 2201.8 | 3 | < 2.2e-16 |
| Tmean_May | -0.29*** | -0.05*** | 0.89*** | 0.24*** | 1.18*** | 0.94*** | 2447.0 | 3 | < 2.2e-16 |
| Tmean_Jun | 0.16*** | -0.33*** | 1.40*** | -0.49*** | 1.24*** | 1.73*** | 2306.9 | 3 | < 2.2e-16 |
| Tmean_Jul | -0.29** | 0.21*** | 1.27*** | 0.50*** | 1.56*** | 1.06*** | 2471.4 | 3 | < 2.2e-16 |
| Tmean_Aug | -0.17 | 0.58*** | 1.66*** | 0.75*** | 1.83*** | 1.08*** | 2409.5 | 3 | < 2.2e-16 |
| Tmean_Sep | 0.11 | 0.10*** | 0.88*** | -0.01*** | 0.77*** | 0.78*** | 2252.7 | 3 | < 2.2e-16 |
| Tmean_Oct | 0.30*** | 0.23*** | 1.32*** | -0.07*** | 1.02*** | 1.09*** | 2481.9 | 3 | < 2.2e-16 |
| Tmean_Nov | 0.40*** | -0.02*** | 1.73*** | -0.42*** | 1.33*** | 1.75*** | 2605.3 | 3 | < 2.2e-16 |
| Tmean_Dec | 0.11*** | 0.50*** | 1.36*** | 0.39*** | 1.25*** | 0.86*** | 2562.1 | 3 | < 2.2e-16 |
| Tmean_Year | 0.01 | 0.08*** | 1.31*** | 0.07*** | 1.30*** | 1.23*** | 2407.1 | 3 | < 2.2e-16 |
|  |  |  |  |  |  |  |  |  |  |
| Tmax_Jan | 0.00 | -1.00*** | 0.40*** | -1.00*** | 0.40*** | 1.40*** | 2116.8 | 3 | < 2.2e-16 |
| Tmax_Feb | -0.10 | 1.10*** | 0.90*** | 1.20*** | 1.00*** | -0.20*** | 2322.1 | 3 | < 2.2e-16 |
| Tmax_Mar | -1.00*** | -1.50*** | 0.10*** | -0.50*** | 1.10*** | 1.60*** | 2344.1 | 3 | < 2.2e-16 |
| Tmax_Apr | -0.70*** | -1.30*** | 2.00*** | -0.60*** | 2.70*** | 3.30*** | 2539.9 | 3 | < 2.2e-16 |
| Tmax_May | -1.80*** | -1.10*** | 0.70*** | 0.70*** | 2.50*** | 1.80*** | 2552.9 | 3 | < 2.2e-16 |
| Tmax_Jun | -0.70*** | -3.10*** | 1.30*** | -2.40*** | 2.00*** | 4.40*** | 2537.7 | 3 | < 2.2e-16 |
| Tmax_Jul | 0.20*** | 1.50*** | 3.00*** | 1.30*** | 2.80*** | 1.30*** | 2455.3 | 3 | < 2.2e-16 |
| Tmax_Aug | -1.30*** | -0.90*** | 1.60*** | 0.40*** | 2.90*** | 2.50*** | 2538.7 | 3 | < 2.2e-16 |
| Tmax_Sep | -1.50*** | -1.00*** | -0.30*** | 0.50*** | 1.20*** | 0.70*** | 2250.6 | 3 | < 2.2e-16 |
| Tmax_Oct | -1.90*** | -1.60*** | -0.40** | 0.30*** | 1.50*** | 1.20*** | 2389.3 | 3 | < 2.2e-16 |
| Tmax_Nov | 0.50*** | 0.20*** | 0.80*** | -0.30*** | 0.30*** | 0.60*** | 1553.3 | 3 | < 2.2e-16 |
| Tmax_Dec | -0.30*** | -1.50*** | 1.90*** | -1.20*** | 2.20*** | 3.40*** | 2598.9 | 3 | < 2.2e-16 |
|  |  |  |  |  |  |  |  |  |  |
| Tmin_Jan | 3.50*** | 5.20*** | 7.90*** | 1.70*** | 4.40*** | 2.70*** | 2663.8 | 3 | < 2.2e-16 |
| Tmin_Feb | -0.60*** | 3.20*** | 7.90*** | 3.80*** | 8.50*** | 4.70*** | 2527.1 | 3 | < 2.2e-16 |
| Tmin_Mar | -0.60*** | -1.00*** | 0.40*** | 0.40*** | 1.00*** | 0.60*** | 1356.4 | 3 | < 2.2e-16 |
| Tmin_Apr | 0.70*** | 0.90*** | 1.40*** | 0.20*** | 0.70*** | 0.50*** | 2260.4 | 3 | < 2.2e-16 |
| Tmin_May | 0.10*** | 0.20*** | 1.50*** | 0.10*** | 1.40*** | 1.30*** | 1750.6 | 3 | < 2.2e-16 |
| Tmin_Jun | 1.00*** | 1.40*** | 2.10*** | 0.40*** | 1.10*** | 0.70*** | 2433.2 | 3 | < 2.2e-16 |
| Tmin_Jul | 0.50*** | 0.60*** | 1.10*** | 0.10*** | 0.60*** | 0.50*** | 2084.8 | 3 | < 2.2e-16 |
| Tmin_Aug | 0.00 | 0.50*** | 1.10*** | 0.50*** | 1.10*** | 0.60*** | 2063.8 | 3 | < 2.2e-16 |
| Tmin_Sep | 1.10*** | 0.95*** | 2.10*** | -0.15*** | 1.00*** | 1.15*** | 2199.8 | 3 | < 2.2e-16 |
| Tmin_Oct | 0.30*** | 1.50*** | 1.00*** | 1.20*** | 0.70*** | -0.50*** | 2375.9 | 3 | < 2.2e-16 |
| Tmin_Nov | 1.90*** | 0.70*** | 3.70*** | -1.20*** | 1.80*** | 3.00*** | 2605.8 | 3 | < 2.2e-16 |
| Tmin_Dec | 0.00 | 1.40*** | 0.40 | 1.40*** | 1.60*** | -1.00*** | 1949.9 | 3 | < 2.2e-16 |
|  |  |  |  |  |  |  |  |  |  |
| Tmax - Tmin_Jan | -3.10*** | -6.30*** | -7.00*** | -3.20*** | -3.90*** | -0.70*** | 158.2 | 3 | < 2.2e-16 |
| Tmax - Tmin_Feb | 0.40*** | -2.80*** | -6.90*** | -3.20*** | -7.30*** | -4.10*** | 148.3 | 3 | < 2.2e-16 |
| Tmax - Tmin_Mar | -0.30*** | -0.90*** | 0.00 | -0.60*** | 0.30*** | 0.90*** | 80.7 | 3 | < 2.2e-16 |
| Tmax - Tmin_Apr | -1.50*** | -2.30*** | 0.60*** | -0.80*** | 2.10*** | 2.90*** | 150.8 | 3 | < 2.2e-16 |
| Tmax - Tmin_May | -2.20*** | -1.40*** | -0.90*** | 0.80*** | 1.30*** | 0.50*** | 112.6 | 3 | < 2.2e-16 |
| Tmax - Tmin_Jun | -1.60*** | -4.40*** | -0.70*** | -2.80*** | 0.90*** | 3.70*** | 140.3 | 3 | < 2.2e-16 |
| Tmax - Tmin_Jul | -0.20*** | 0.70*** | 1.90*** | 0.90*** | 2.10*** | 1.20*** | 132.1 | 3 | < 2.2e-16 |
| Tmax - Tmin_Aug | -1.40*** | -1.40*** | 0.50*** | 0.00 | 1.90*** | 1.90*** | 132.5 | 3 | < 2.2e-16 |
| Tmax - Tmin_Sep | -2.40*** | -2.00*** | -2.50*** | 0.40* | -0.10 | -0.50*** | 114.2 | 3 | < 2.2e-16 |
| Tmax - Tmin_Oct | -2.20*** | -3.00*** | -1.20*** | -0.80*** | 1.00*** | 1.80*** | 152.1 | 3 | < 2.2e-16 |
| Tmax - Tmin_Nov | -1.50*** | -0.40*** | -3.10*** | 1.10*** | -1.60*** | -2.70*** | 148.7 | 3 | < 2.2e-16 |
| Tmax - Tmin_Dec | -0.30 | -3.10*** | 1.60*** | -2.80*** | 1.90*** | 4.70*** | 148.0 | 3 | < 2.2e-16 |

**Table S3**. Climatic variables of presences and absences for 1900–1949. Shown are medians (m_presence_, m_absence_) as well as minima (min_presence_, min_absence_) and maxima (max_presence_, max_absence_). Results of Wilcoxon tests of differences between medians of presences and absences (W, p). Red = non-significant p-value.

|  | m_presence_ | min_presence_ | max_presence_ | m_absence_ | min_absence_ | max_absence_ | W | p |
| --- | --- | --- | --- | --- | --- | --- | --- | --- |
| Precip_Jan | 62.40 | 39.47 | 110.74 | 63.53 | 43.81 | 124.34 | 332 | 0.868 |
| Precip_Feb | 50.41 | 34.00 | 89.55 | 51.82 | 38.11 | 97.80 | 316 | 0.655 |
| Precip_Mar | 52.63 | 32.42 | 79.76 | 47.54 | 34.95 | 85.84 | 301 | 0.477 |
| Precip_Apr | 58.13 | 43.26 | 91.73 | 54.68 | 41.97 | 92.83 | 285 | 0.320 |
| Precip_May | 63.55 | 47.09 | 93.91 | 61.52 | 49.17 | 91.39 | 269 | 0.201 |
| Precip_Jun | 71.81 | 50.94 | 103.79 | 69.13 | 55.10 | 107.90 | 303 | 0.499 |
| Precip_Jul | 77.51 | 61.76 | 124.05 | 76.82 | 61.01 | 126.29 | 284 | 0.312 |
| Precip_Aug | 79.69 | 54.24 | 118.12 | 76.57 | 60.36 | 119.51 | 304 | 0.510 |
| Precip_Sep | 61.10 | 40.64 | 92.15 | 57.58 | 45.70 | 100.01 | 297 | 0.434 |
| Precip_Oct | 67.92 | 45.07 | 104.91 | 63.91 | 48.30 | 119.60 | 306 | 0.533 |
| Precip_Nov | 66.56 | 44.89 | 105.13 | 61.93 | 47.48 | 119.47 | 325 | 0.770 |
| Precip_Dec | 65.51 | 44.13 | 114.62 | 64.30 | 48.10 | 124.41 | 322 | 0.732 |
| Precip_Year | 804.00 | 554.8 | 1213.6 | 749.9 | 587.50 | 1309.40 | 309 | 0.568 |
|  |  |  |  |  |  |  |  |  |
| Tmean_Jan | -0.56 | -3.05 | 0.59 | -0.19 | -2.08 | 0.73 | 493 | 0.007 |
| Tmean_Feb | 0.21 | -2.41 | 1.69 | 0.69 | -1.25 | 1.81 | 499 | 0.005 |
| Tmean_Mar | 3.49 | 0.69 | 5.26 | 4.05 | 2.07 | 5.41 | 507 | 0.004 |
| Tmean_Apr | 7.40 | 4.46 | 9.14 | 7.99 | 5.78 | 9.23 | 514 | 0.002 |
| Tmean_May | 12.18 | 9.44 | 13.84 | 12.72 | 10.71 | 13.90 | 515 | 0.002 |
| Tmean_Jun | 14.92 | 12.21 | 16.66 | 15.54 | 13.45 | 16.63 | 516 | 0.002 |
| Tmean_Jul | 16.72 | 13.89 | 18.42 | 17.25 | 15.13 | 18.39 | 513 | 0.002 |
| Tmean_Aug | 16.01 | 13.31 | 17.50 | 16.41 | 14.50 | 17.60 | 511 | 0.002 |
| Tmean_Sep | 12.86 | 10.56 | 14.27 | 13.23 | 11.86 | 14.43 | 497 | 0.005 |
| Tmean_Oct | 8.17 | 5.93 | 9.305 | 8.47 | 7.14 | 9.56 | 487 | 0.010 |
| Tmean_Nov | 3.34 | 0.91 | 4.51 | 3.75 | 1.94 | 4.55 | 488 | 0.009 |
| Tmean_Dec | 0.52 | -1.90 | 1.55 | 0.84 | -1.07 | 1.66 | 476 | 0.017 |
| Tmean_Year | 7.97 | 5.34 | 9.39 | 8.39 | 6.51 | 9.48 | 503 | 0.004 |
|  |  |  |  |  |  |  |  |  |
| Tmax_Jan | 7.00 | 4.60 | 8.60 | 7.35 | 5.60 | 8.70 | 405.5 | 0.264 |
| Tmax_Feb | 8.50 | 6.00 | 10.40 | 9.20 | 7.50 | 10.90 | 506.5 | 0.004 |
| Tmax_Mar | 13.10 | 10.30 | 15.70 | 14.00 | 11.70 | 15.30 | 486 | 0.011 |
| Tmax_Apr | 17.60 | 15.10 | 19.30 | 18.10 | 15.90 | 19.60 | 464 | 0.031 |
| Tmax_May | 22.00 | 19.60 | 23.70 | 22.50 | 20.40 | 24.40 | 480.5 | 0.014 |
| Tmax_Jun | 25.50 | 22.90 | 26.40 | 25.90 | 23.50 | 31.10 | 467 | 0.027 |
| Tmax_Jul | 26.40 | 24.00 | 28.50 | 26.90 | 24.90 | 29.00 | 449 | 0.059 |
| Tmax_Aug | 26.90 | 24.40 | 28.60 | 27.50 | 25.30 | 29.30 | 473 | 0.021 |
| Tmax_Sep | 24.10 | 21.40 | 25.60 | 24.70 | 22.60 | 26.10 | 475 | 0.019 |
| Tmax_Oct | 17.80 | 15.50 | 19.40 | 17.60 | 16.10 | 19.50 | 363.5 | 0.710 |
| Tmax_Nov | 10.20 | 8.00 | 11.00 | 10.30 | 8.70 | 11.90 | 392 | 0.380 |
| Tmax_Dec | 7.60 | 5.10 | 8.70 | 7.90 | 6.50 | 9.30 | 470.5 | 0.023 |
|  |  |  |  |  |  |  |  |  |
| Tmin_Jan | -14.60 | -18.00 | -12.70 | -14.70 | -17.40 | -11.90 | 349.5 | 0.901 |
| Tmin_Feb | -14.10 | -16.40 | -11.80 | -14.00 | -16.30 | -11.60 | 413 | 0.212 |
| Tmin_Mar | -5.20 | -7.10 | -1.90 | -3.65 | -5.90 | -2.50 | 573 | 4.4*10^-5^ |
| Tmin_Apr | -1.40 | -3.20 | 1.50 | -0.10 | -1.90 | 1.20 | 579.5 | 2.7*10^-5^ |
| Tmin_May | 2.90 | 1.00 | 4.30 | 3.50 | 2.00 | 4.40 | 481.5 | 0.014 |
| Tmin_Jun | 6.10 | 4.60 | 8.10 | 7.00 | 5.10 | 8.10 | 558 | 1.3*10^-4^ |
| Tmin_Jul | 8.50 | 7.10 | 10.50 | 9.30 | 7.80 | 10.40 | 567 | 6.9*10^-5^ |
| Tmin_Aug | 8.50 | 7.00 | 10.60 | 9.30 | 2.00 | 10.90 | 540.5 | 4.5*10^-4^ |
| Tmin_Sep | 4.10 | 2.90 | 5.90 | 5.05 | 3.50 | 5.90 | 559 | 1.2*10^-4^ |
| Tmin_Oct | 0.70 | -1.00 | 2.30 | 1.50 | -0.50 | 2.20 | 533 | 7.3*10^-4^ |
| Tmin_Nov | -4.10 | -6.00 | -2.20 | -3.50 | -5.20 | -2.30 | 510 | 0.003 |
| Tmin_Dec | -8.10 | -9.10 | -5.60 | -6.80 | -8.70 | -5.90 | 598 | 6.0*10^-6^ |
|  |  |  |  |  |  |  |  |  |
| Tmax - Tmin_Jan | 21.40 | 18.70 | 25.80 | 21.75 | 18.40 | 25.00 | 400 | 0.309 |
| Tmax - Tmin_Feb | 22.50 | 19.30 | 25.00 | 23.00 | 19.70 | 24.90 | 403 | 0.284 |
| Tmax - Tmin_Mar | 18.10 | 16.60 | 20.50 | 17.65 | 16.30 | 19.00 | 239 | 0.069 |
| Tmax - Tmin_Apr | 18.60 | 17.20 | 20.70 | 18.10 | 17.20 | 19.40 | 155.5 | 0.001 |
| Tmax - Tmin_May | 19.30 | 17.80 | 20.70 | 19.05 | 17.90 | 20.40 | 295 | 0.410 |
| Tmax - Tmin_Jun | 19.20 | 18.20 | 20.60 | 18.75 | 17.60 | 24.50 | 294 | 0.400 |
| Tmax - Tmin_Jul | 17.70 | 16.80 | 20.50 | 17.45 | 16.40 | 19.30 | 253.5 | 0.119 |
| Tmax - Tmin_Aug | 18.20 | 17.30 | 20.50 | 18.05 | 16.40 | 27.30 | 300.5 | 0.467 |
| Tmax - Tmin_Sep | 19.40 | 18.50 | 22.00 | 19.60 | 18.20 | 20.70 | 398 | 0.326 |
| Tmax - Tmin_Oct | 17.00 | 15.90 | 18.20 | 16.10 | 14.90 | 18.20 | 133.5 | 2.3*10^-4^ |
| Tmax - Tmin_Nov | 14.10 | 13.10 | 15.10 | 13.80 | 12.60 | 15.10 | 195 | 0.009 |
| Tmax - Tmin_Dec | 15.00 | 13.90 | 16.70 | 14.80 | 13.90 | 16.50 | 256 | 0.130 |

**Table S4**. Climatic variables of presences and absences for 1950–1979. Shown are medians (m_presence_, m_absence_) as well as minima (min_presence_, min_absence_) and maxima (max_presence_, max_absence_). Results of Wilcoxon tests of differences between medians of presences and absences (W, p). Red = non-significant p-value.

|  | m_presence_ | min_presence_ | max_presence_ | m_absence_ | min_absence_ | max_absence_ | W | P |
| --- | --- | --- | --- | --- | --- | --- | --- | --- |
| Precip_Jan | 83.74 | 66.76 | 105.28 | 59.37 | 40.59 | 114.76 | 38 | 3.9*10^-4^ |
| Precip_Feb | 73.18 | 60.61 | 92.96 | 53.29 | 36.97 | 100.15 | 35 | 2.5*10^-4^ |
| Precip_Mar | 68.22 | 57.33 | 85.79 | 49.98 | 35.71 | 89.40 | 29 | 9.9*10^-5^ |
| Precip_Apr | 72.23 | 65.69 | 93.67 | 53.96 | 41.34 | 99.05 | 25 | 4.9*10^-5^ |
| Precip_May | 78.66 | 72.85 | 100.31 | 66.47 | 50.65 | 93.55 | 45 | 9.5*10^-4^ |
| Precip_Jun | 97.25 | 88.59 | 121.70 | 76.94 | 61.39 | 120.97 | 28 | 8.4*10^-5^ |
| Precip_Jul | 89.03 | 81.37 | 114.43 | 75.22 | 57.05 | 123.48 | 56 | 0.003 |
| Precip_Aug | 95.82 | 87.00 | 120.34 | 81.84 | 59.46 | 123.36 | 36 | 2.9*10^-4^ |
| Precip_Sep | 73.79 | 66.72 | 96.11 | 57.27 | 40.20 | 103.36 | 28 | 8.4*10^-5^ |
| Precip_Oct | 72.92 | 66.34 | 96.46 | 54.69 | 41.39 | 107.40 | 20 | 1.8*10^-5^ |
| Precip_Nov | 89.34 | 70.16 | 110.70 | 73.10 | 48.36 | 132.18 | 67 | 0.009 |
| Precip_Dec | 108.79 | 83.94 | 135.75 | 75.31 | 48.12 | 152.99 | 26 | 5.9*10^-5^ |
| Precip_Year | 980.50 | 868.20 | 1265.20 | 776.30 | 587.90 | 1360.60 | 31 | 1.4*10^-4^ |
|  |  |  |  |  |  |  |  |  |
| Tmean_Jan | -2.30 | -3.19 | -1.65 | -0.17 | -2.08 | 0.87 | 334 | 3.9*10^-8^ |
| Tmean_Feb | -1.49 | -2.42 | -0.63 | 0.70 | -1.18 | 2.10 | 335 | 2.0*10^-8^ |
| Tmean_Mar | 1.56 | 0.59 | 2.65 | 3.90 | 2.01 | 5.56 | 335 | 2.0*10^-8^ |
| Tmean_Apr | 5.48 | 4.58 | 6.83 | 7.76 | 5.76 | 9.42 | 331 | 1.9*10^-7^ |
| Tmean_May | 10.06 | 9.15 | 11.36 | 12.13 | 10.31 | 13.75 | 331 | 1.9*10^-7^ |
| Tmean_Jun | 13.39 | 12.64 | 14.81 | 15.53 | 13.62 | 17.00 | 332 | 1.2*10^-7^ |
| Tmean_Jul | 14.91 | 14.05 | 16.27 | 16.95 | 15.05 | 18.62 | 329 | 4.4*10^-7^ |
| Tmean_Aug | 14.35 | 13.54 | 15.54 | 16.27 | 14.65 | 17.88 | 331 | 1.9*10^-7^ |
| Tmean_Sep | 11.40 | 10.65 | 12.46 | 13.18 | 11.84 | 14.78 | 331 | 1.9*10^-7^ |
| Tmean_Oct | 7.17 | 6.44 | 7.86 | 8.74 | 7.64 | 10.13 | 335 | 2.0*10^-8^ |
| Tmean_Nov | 2.03 | 1.20 | 2.80 | 4.11 | 2.25 | 5.12 | 334 | 3.9*10^-8^ |
| Tmean_Dec | -1.02 | -1.81 | -0.35 | 1.01 | -0.91 | 1.88 | 334 | 3.9*10^-8^ |
| Tmean_Year | 6.30 | 5.45 | 7.33 | 8.31 | 6.58 | 9.76 | 335 | 2.0*10^-8^ |
|  |  |  |  |  |  |  |  |  |
| Tmax_Jan | 4.90 | 4.20 | 5.40 | 7.40 | 5.60 | 8.90 | 336 | 2.2*10^-5^ |
| Tmax_Feb | 7.30 | 6.20 | 8.00 | 9.00 | 7.60 | 11.00 | 330 | 4.4*10^-5^ |
| Tmax_Mar | 10.70 | 10.00 | 11.90 | 13.05 | 11.60 | 14.50 | 333 | 3.2*10^-5^ |
| Tmax_Apr | 15.00 | 14.10 | 16.00 | 17.25 | 15.50 | 18.60 | 333 | 3.2*10^-5^ |
| Tmax_May | 18.60 | 17.30 | 19.50 | 20.60 | 18.90 | 22.50 | 332.5 | 3.4*10^-5^ |
| Tmax_Jun | 22.30 | 21.60 | 23.60 | 25.05 | 22.80 | 26.90 | 330 | 4.4*10^-5^ |
| Tmax_Jul | 24.80 | 24.10 | 26.10 | 27.00 | 25.50 | 28.50 | 331 | 4.0*10^-5^ |
| Tmax_Aug | 23.40 | 22.60 | 24.50 | 26.20 | 24.30 | 27.20 | 335 | 2.5*10^-5^ |
| Tmax_Sep | 21.20 | 20.30 | 22.20 | 23.00 | 21.40 | 24.70 | 331.5 | 3.7e*10^-5^ |
| Tmax_Oct | 14.00 | 13.20 | 14.60 | 15.90 | 14.70 | 17.70 | 336 | 2.2*10^-5^ |
| Tmax_Nov | 9.00 | 8.00 | 9.50 | 10.80 | 9.50 | 12.30 | 335.5 | 2.4*10^-5^ |
| Tmax_Dec | 5.30 | 4.70 | 5.80 | 7.70 | 5.60 | 9.60 | 335 | 2.5*10^-5^ |
|  |  |  |  |  |  |  |  |  |
| Tmin_Jan | -12.40 | -12.80 | -11.80 | -11.20 | -13.00 | -9.40 | 301 | 8.0*10^-4^ |
| Tmin_Feb | -15.40 | -15.80 | -14.90 | -14.50 | -16.70 | -13.50 | 271 | 0.009 |
| Tmin_Mar | -6.90 | -7.40 | -6.30 | -4.30 | -6.40 | -2.80 | 334 | 2.9*10^-5^ |
| Tmin_Apr | -1.40 | -2.20 | -0.60 | 0.50 | -1.20 | 1.80 | 330 | 4.4*10^-5^ |
| Tmin_May | 2.10 | 1.90 | 2.80 | 3.60 | 2.20 | 5.30 | 329 | 5.0*10^-5^ |
| Tmin_Jun | 6.10 | 6.10 | 6.90 | 7.90 | 6.00 | 9.50 | 318 | 1.5*10^-4^ |
| Tmin_Jul | 7.80 | 7.40 | 8.70 | 9.70 | 8.10 | 10.90 | 331.5 | 3.7*10^-5^ |
| Tmin_Aug | 8.00 | 7.50 | 8.60 | 9.20 | 7.80 | 10.90 | 315.5 | 2.0*10^-4^ |
| Tmin_Sep | 4.80 | 4.20 | 5.70 | 6.00 | 3.70 | 7.20 | 281 | 0.004 |
| Tmin_Oct | 0.50 | -0.20 | 1.80 | 1.75 | 0.20 | 3.70 | 290 | 0.002 |
| Tmin_Nov | -3.60 | -4.10 | -2.60 | -1.65 | -8.50 | 0.00 | 323.5 | 8.8*10^-5^ |
| Tmin_Dec | -9.30 | -10.10 | -8.30 | -6.90 | -9.60 | -5.80 | 312 | 2.8*10^-4^ |
|  |  |  |  |  |  |  |  |  |
| Tmax - Tmin_Jan | 17.20 | 16.40 | 17.80 | 18.45 | 16.30 | 20.50 | 299.5 | 9.3*10^-4^ |
| Tmax - Tmin_Feb | 22.50 | 21.80 | 23.20 | 23.40 | 21.80 | 26.80 | 270 | 0.010 |
| Tmax - Tmin_Mar | 17.40 | 16.90 | 18.20 | 17.45 | 16.30 | 18.80 | 151.5 | 0.685 |
| Tmax - Tmin_Apr | 16.30 | 15.90 | 16.60 | 16.70 | 15.70 | 19.20 | 254 | 0.031 |
| Tmax - Tmin_May | 16.20 | 15.40 | 16.70 | 17.00 | 15.80 | 19.20 | 293.5 | 0.002 |
| Tmax - Tmin_Jun | 15.90 | 15.50 | 16.70 | 17.30 | 15.50 | 20.40 | 295 | 0.001 |
| Tmax - Tmin_Jul | 16.90 | 16.40 | 17.40 | 17.40 | 15.90 | 20.40 | 258.5 | 0.023 |
| Tmax - Tmin_Aug | 15.50 | 15.10 | 15.90 | 16.80 | 15.40 | 18.80 | 320.5 | 1.2*10^-4^ |
| Tmax - Tmin_Sep | 16.50 | 15.70 | 16.70 | 17.20 | 15.60 | 19.50 | 286 | 0.003 |
| Tmax - Tmin_Oct | 13.20 | 12.80 | 14.00 | 14.15 | 13.00 | 15.90 | 285 | 0.003 |
| Tmax - Tmin_Nov | 12.60 | 12.10 | 12.80 | 12.35 | 11.30 | 20.80 | 156.5 | 0.781 |
| Tmax - Tmin_Dec | 14.50 | 13.90 | 14.80 | 14.60 | 13.50 | 17.40 | 210.5 | 0.288 |

**Figure S4**. Results of the logistic regression analysis of the relation between climatic variables and presences/absences of *T. helenitis* for the period 1900–1949. A) Monthly precipitation sums, B) monthly mean temperatures, C) monthly maximum temperatures, D) monthly minimum temperatures, E) monthly temperature ranges (maximum – minimum monthly temperature), F) annual precipitation sum and annual mean temperature. Shown are presences (N=19) and absences (N=36) used to establish models, the logistic regression (logit) curve fitted, the threshold of the model (i.e., the value of the climatic variable for which the model predicts an occurrence probability of 0.5, dotted line) as well as the model’s ΔAIC value for goodness of fit or n.s. when the fit was not significant. For detailed statistics and goodness of fit of models, see Tables S5 and S6.

A)

**
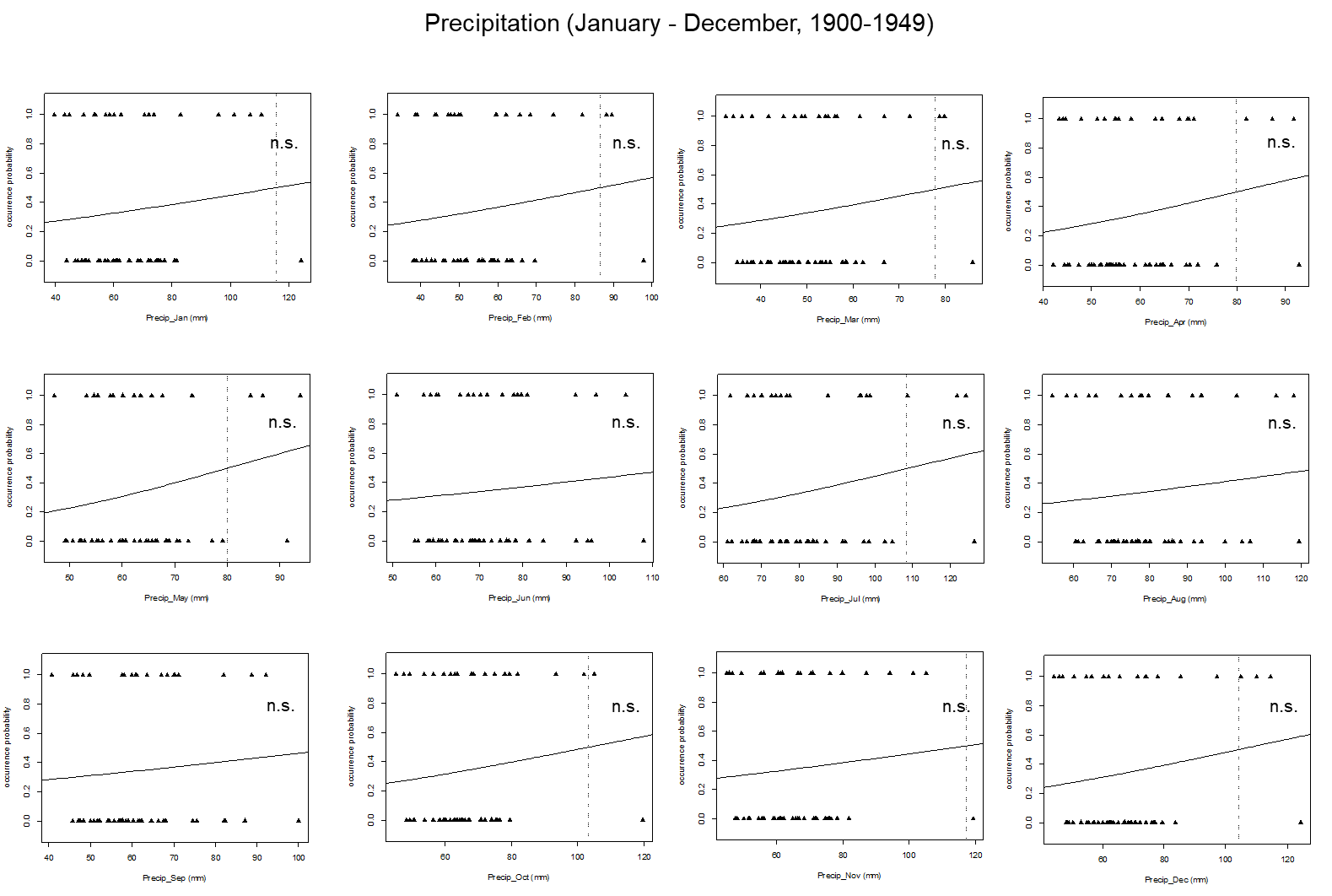
**

B)


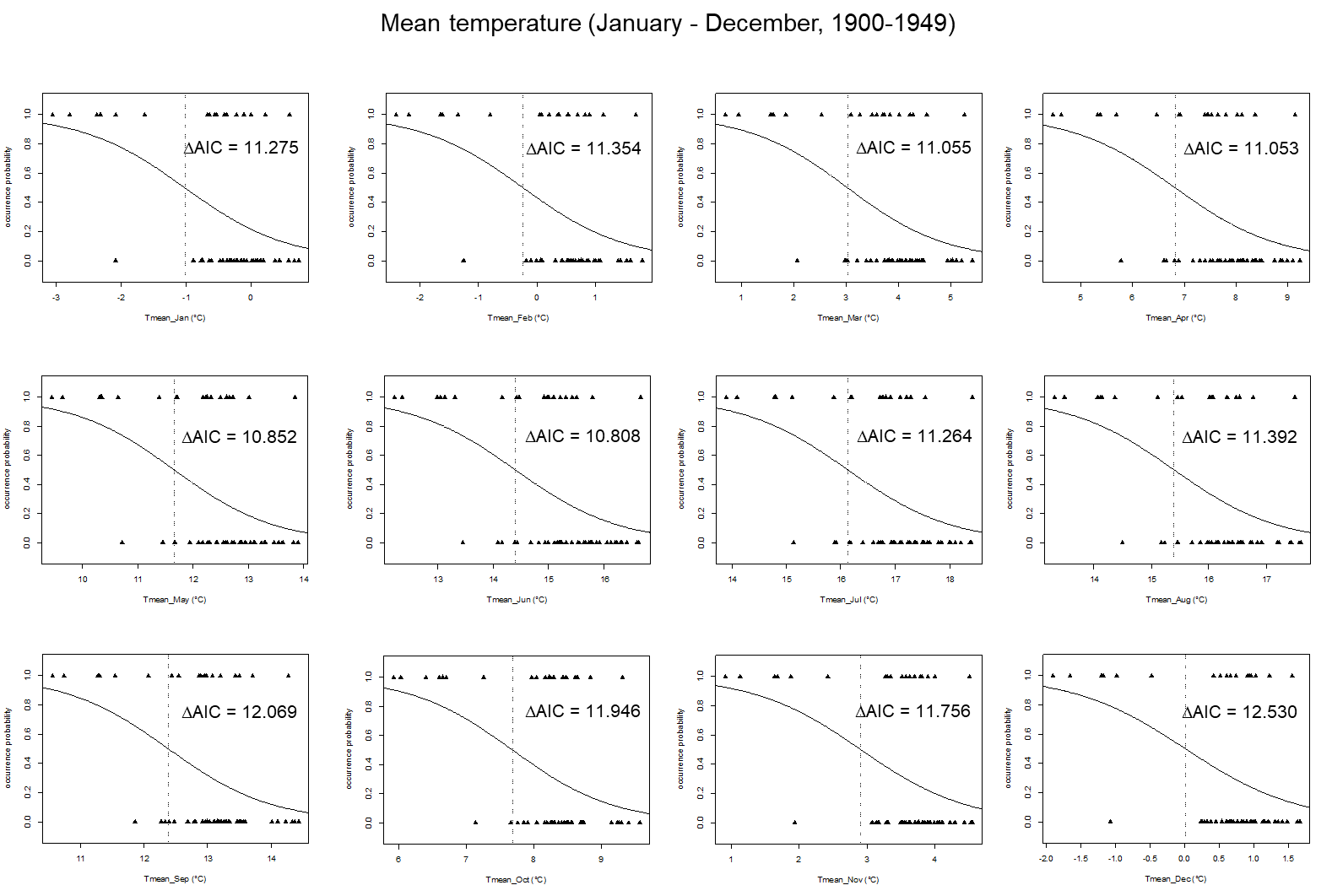


C)


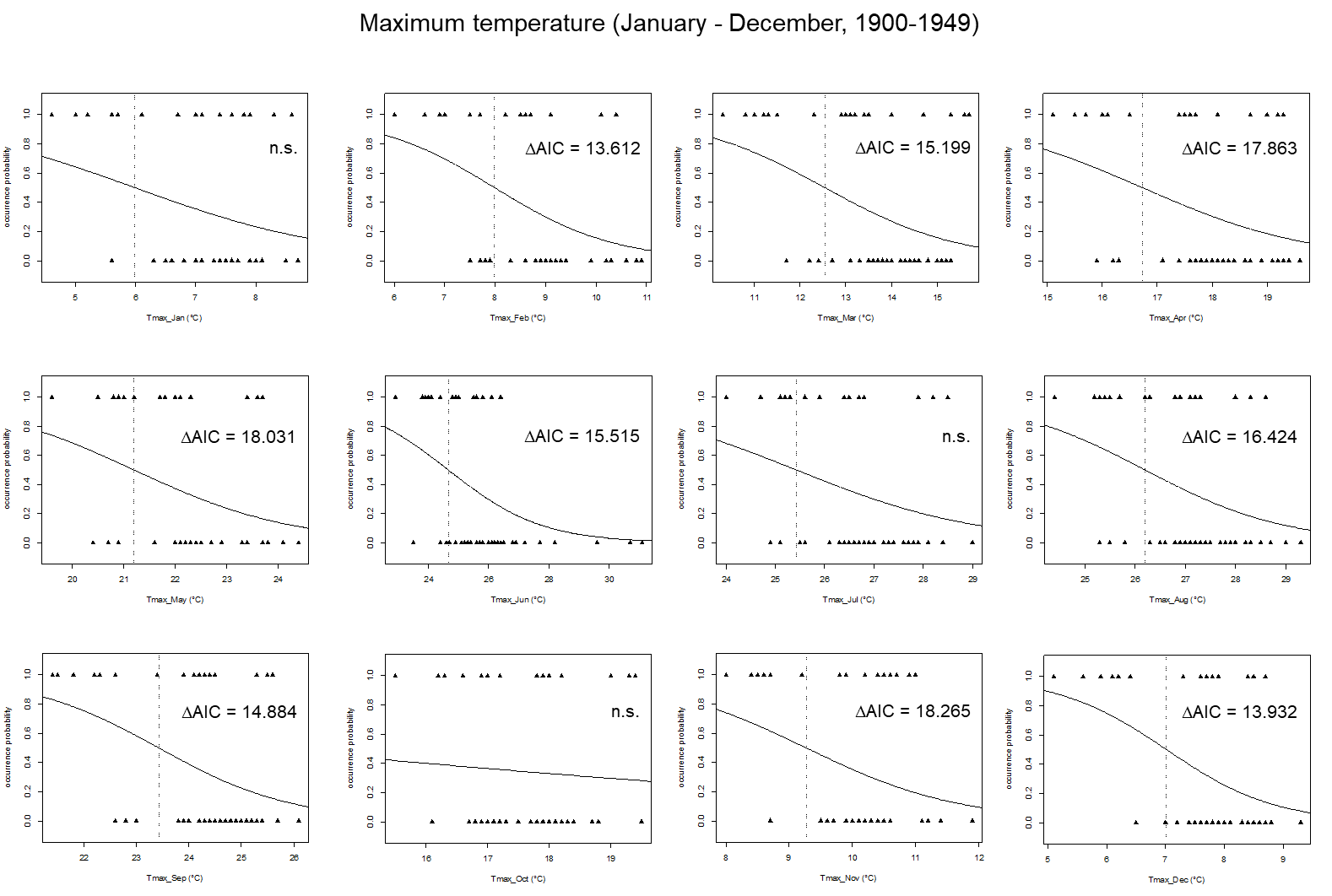


D)


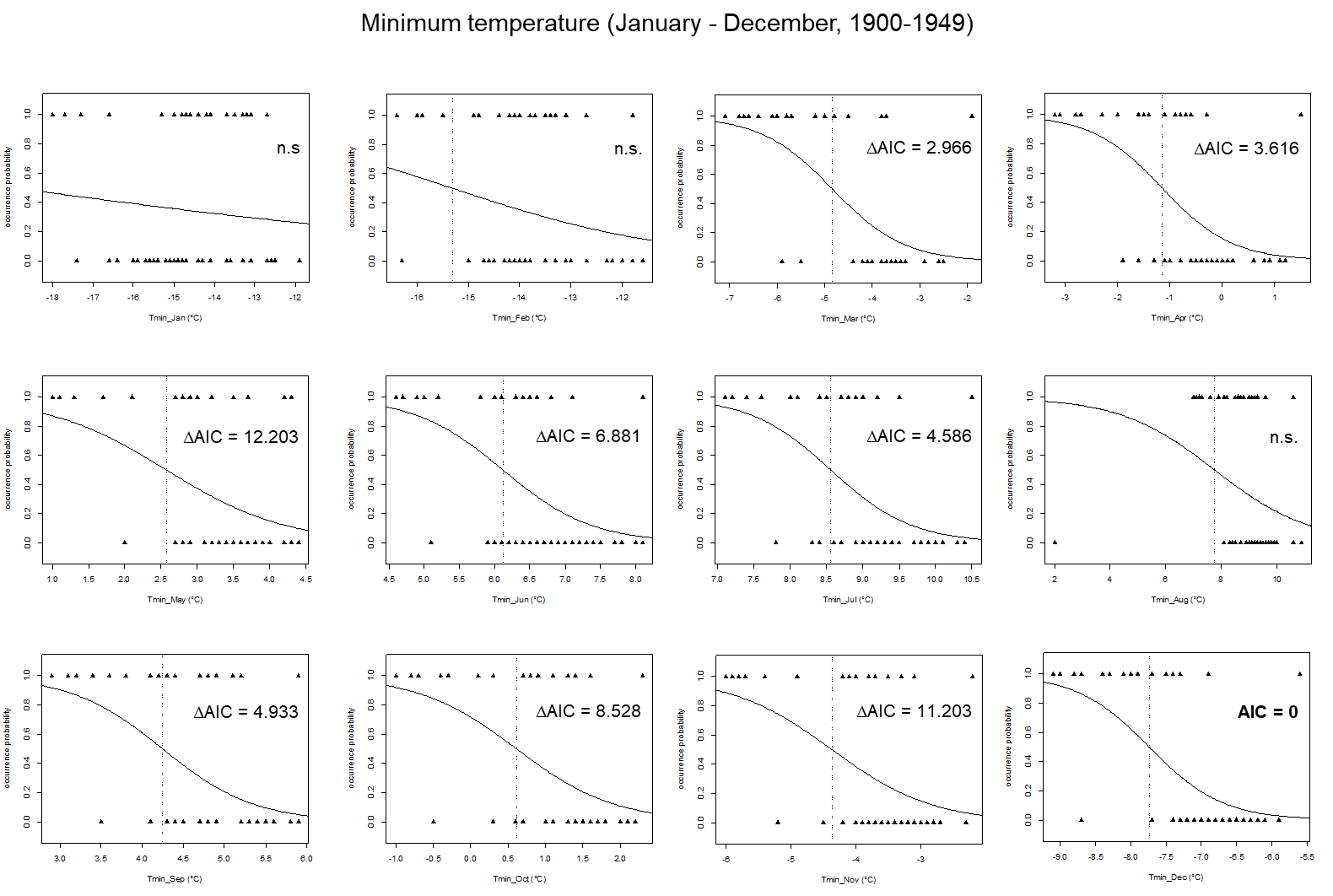


E)


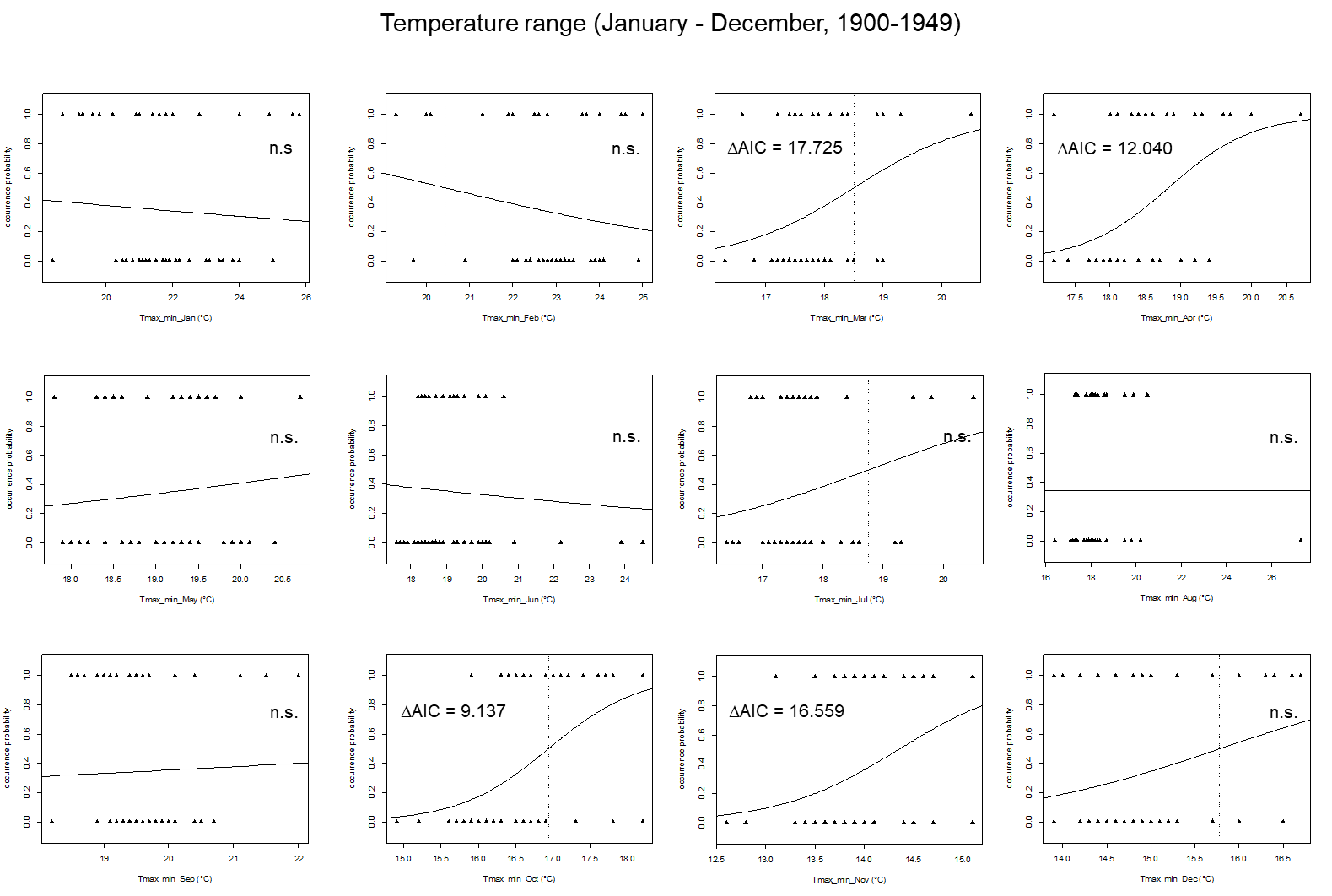


F)


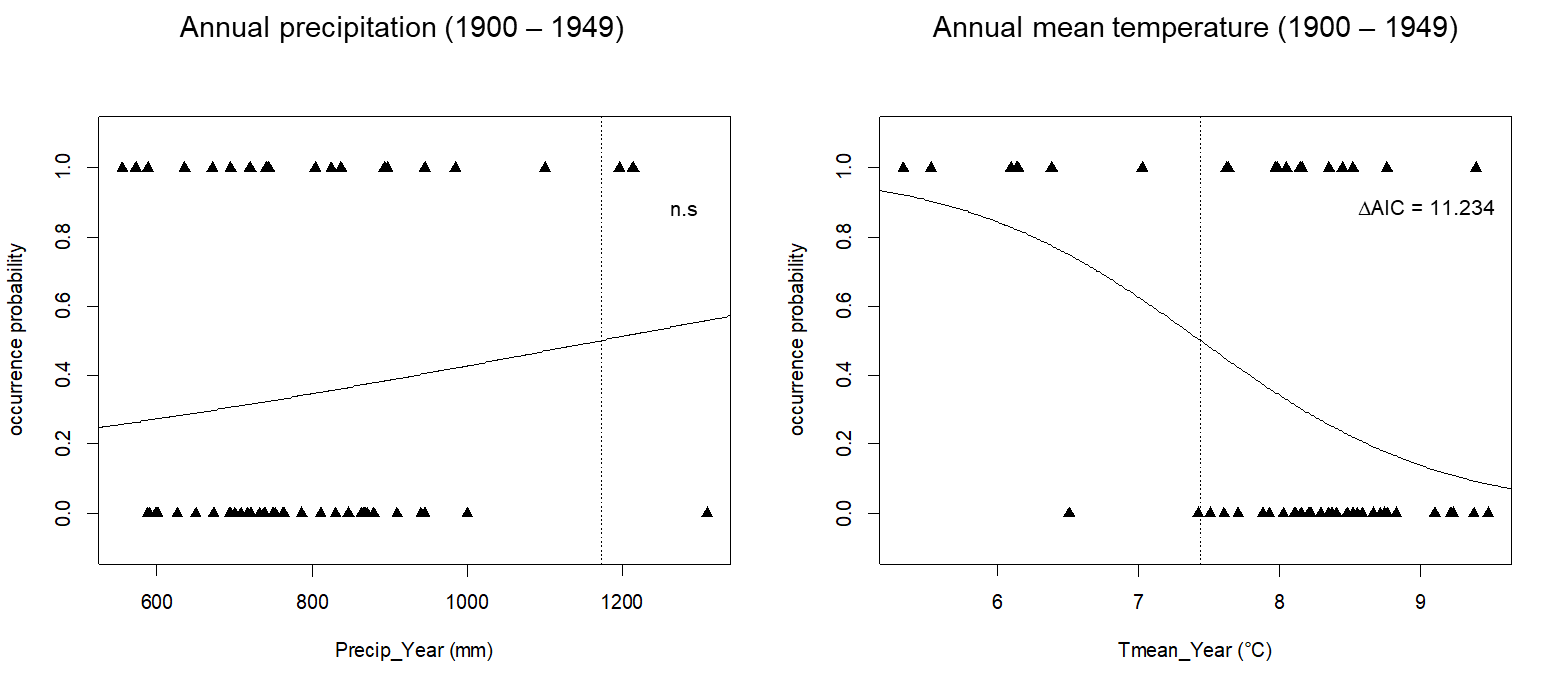


**Table S5**. Results of the logistic regression analysis of the relation between climatic variables and presences/absences of *T. helenitis* for the period 1900–1949. Shown are the intercepts and predictors (slope) with their standard errors (s.e.) and significance tests (p), as well as AIC and ΔAIC values. Residual deviance, null deviance, LRT and p are the results of the respective likelihood-ratio test. Only models with significant predictors and likelihood-ratio tests were passed to AIC-based model selection. Red = non-significant p-value or ΔAIC value, respectively. Blue = 2 ≤ ΔAIC < 10 (model selection, Burnham & Anderson, 2002). The number of presences is 19 and the number of absences is 36.

|  | intercept | s.e. | p | predictor | s.e. | p | AIC | Residual  deviance | Null  deviance | LRT | p | ΔAIC |
| --- | --- | --- | --- | --- | --- | --- | --- | --- | --- | --- | --- | --- |
| Precip_Jan | -1.510 | 1.140 | 0.185 | 0.013 | 0.016 | 0.427 | 74.273 | 70.273 | 70.905 | 0.632 | 0.427 | n.s. |
| Precip_Feb | -1.790 | 1.230 | 0.146 | 0.021 | 0.021 | 0.333 | 73.960 | 69.960 | 70.905 | 0.945 | 0.331 | n.s. |
| Precip_Mar | -1.877 | 1.299 | 0.149 | 0.024 | 0.025 | 0.325 | 73.928 | 69.928 | 70.905 | 0.977 | 0.323 | n.s. |
| Precip_Apr | -2.497 | 1.460 | 0.087 | 0.031 | 0.024 | 0.191 | 73.152 | 69.152 | 70.905 | 1.752 | 0.186 | n.s. |
| Precip_May | -3.277 | 1.832 | 0.074 | 0.041 | 0.028 | 0.143 | 72.667 | 68.667 | 70.905 | 2.238 | 0.135 | n.s. |
| Precip_Jun | -1.644 | 1.649 | 0.319 | 0.014 | 0.022 | 0.534 | 74.520 | 70.520 | 70.905 | 0.385 | 0.535 | n.s. |
| Precip_Jul | -2.689 | 1.555 | 0.084 | 0.025 | 0.018 | 0.177 | 73.037 | 69.037 | 70.905 | 1.867 | 0.172 | n.s. |
| Precip_Aug | -1.797 | 1.549 | 0.246 | 0.014 | 0.019 | 0.444 | 74.319 | 70.319 | 70.905 | 0.585 | 0.444 | n.s. |
| Precip_Sep | -1.448 | 1.396 | 0.300 | 0.013 | 0.022 | 0.552 | 74.552 | 70.552 | 70.905 | 0.352 | 0.553 | n.s. |
| Precip_Oct | -1.849 | 1.385 | 0.182 | 0.018 | 0.020 | 0.369 | 74.092 | 70.092 | 70.905 | 0.813 | 0.367 | n.s. |
| Precip_Nov | -1.490 | 1.299 | 0.252 | 0.013 | 0.019 | 0.500 | 74.451 | 70.451 | 70.905 | 0.454 | 0.501 | n.s. |
| Precip_Dec | -1.878 | 1.195 | 0.116 | 0.018 | 0.017 | 0.282 | 73.733 | 69.733 | 70.905 | 1.171 | 0.279 | n.s. |
| Precip_Year | -2.004 | 1.435 | 0.162 | 0.002 | 0.002 | 0.329 | 73.942 | 69.942 | 70.905 | 0.962 | 0.327 | n.s. |
|  |  |  |  |  |  |  |  |  |  |  |  |  |
| Tmean_Jan | -1.300 | 0.383 | 7.0*10^-4^ | -1.273 | 0.447 | 0.004 | 63.065 | 59.065 | 70.905 | 11.840 | 5.8*10^-4^ | 11.275 |
| Tmean_Feb | -0.271 | 0.349 | 0.437 | -1.150 | 0.401 | 0.004 | 63.144 | 59.144 | 70.905 | 11.761 | 6.1*10^-4^ | 11.354 |
| Tmean_Mar | 3.205 | 1.356 | 0.018 | -1.059 | 0.362 | 0.003 | 62.845 | 58.845 | 70.905 | 12.059 | 5.1*10^-4^ | 11.055 |
| Tmean_Apr | 6.874 | 2.564 | 0.007 | -1.008 | 0.341 | 0.003 | 62.843 | 58.843 | 70.905 | 12.061 | 5.1*10^-4^ | 11.053 |
| Tmean_May | 12.793 | 4.533 | 0.005 | -1.097 | 0.370 | 0.003 | 62.642 | 58.642 | 70.905 | 12.262 | 4.6*10^-4^ | 10.852 |
| Tmean_Jun | 15.413 | 5.377 | 0.004 | -1.070 | 0.358 | 0.003 | 62.598 | 58.598 | 70.905 | 12.307 | 4.5*10^-4^ | 10.808 |
| Tmean_Jul | 16.849 | 5.946 | 0.005 | -1.045 | 0.355 | 0.003 | 63.054 | 59.054 | 70.905 | 11.850 | 5.8*10^-4^ | 11.264 |
| Tmean_Aug | 16.957 | 6.028 | 0.005 | -1.101 | 0.377 | 0.003 | 63.182 | 59.182 | 70.905 | 11.722 | 6.2*10^-4^ | 11.392 |
| Tmean_Sep | 15.318 | 5.626 | 0.006 | -1.238 | 0.436 | 0.005 | 63.859 | 59.859 | 70.905 | 11.046 | 8.9*10^-4^ | 12.069 |
| Tmean_Oct | 10.307 | 3.915 | 0.008 | -1.341 | 0.477 | 0.005 | 63.736 | 59.736 | 70.905 | 11.168 | 8.3*10^-4^ | 11.946 |
| Tmean_Nov | 3.705 | 1.593 | 0.020 | -1.275 | 0.453 | 0.005 | 63.546 | 59.546 | 70.905 | 11.359 | 7.5*10^-4^ | 11.756 |
| Tmean_Dec | 0.016 | 0.405 | 0.969 | -1.238 | 0.448 | 0.006 | 64.320 | 60.320 | 70.905 | 10.585 | 0.001 | 12.530 |
| Tmean_Year | 8.703 | 3.240 | 0.007 | -1.170 | 0.403 | 0.004 | 63.024 | 59.024 | 70.905 | 11.880 | 5.7*10^-4^ | 11.234 |
|  |  |  |  |  |  |  |  |  |  |  |  |  |
| Tmax_Jan | 3.556 | 2.329 | 0.127 | -0.594 | 0.329 | 0.071 | 71.412 | 67.412 | 70.905 | 3.492 | 0.062 | n.s. |
| Tmax_Feb | 6.720 | 2.655 | 0.011 | -0.841 | 0.304 | 0.006 | 65.402 | 61.402 | 70.905 | 9.503 | 0.002 | 13.612 |
| Tmax_Mar | 8.551 | 3.571 | 0.017 | -0.682 | 0.265 | 0.010 | 66.989 | 62.989 | 70.905 | 7.915 | 0.005 | 15.199 |
| Tmax_Apr | 10.932 | 5.337 | 0.041 | -0.653 | 0.302 | 0.030 | 69.653 | 65.653 | 70.905 | 5.252 | 0.022 | 17.863 |
| Tmax_May | 13.795 | 6.780 | 0.042 | -0.651 | 0.306 | 0.034 | 69.821 | 65.821 | 70.905 | 5.084 | 0.024 | 18.031 |
| Tmax_Jun | 15.902 | 7.266 | 0.029 | -0.645 | 0.285 | 0.024 | 67.305 | 63.305 | 70.905 | 7.600 | 0.006 | 15.515 |
| Tmax_Jul | 13.744 | 7.707 | 0.075 | -0.541 | 0.290 | 0.063 | 71.108 | 67.108 | 70.905 | 3.800 | 0.051 | n.s. |
| Tmax_Aug | 18.834 | 8.072 | 0.020 | -0.719 | 0.299 | 0.016 | 68.214 | 64.214 | 70.905 | 6.690 | 0.010 | 16.424 |
| Tmax_Sep | 18.430 | 7.310 | 0.012 | -0.787 | 0.302 | 0.009 | 66.674 | 62.674 | 70.905 | 8.231 | 0.004 | 14.884 |
| Tmax_Oct | 2.039 | 5.950 | 0.732 | -0.153 | 0.339 | 0.653 | 74.701 | 70.701 | 70.905 | 0.204 | 0.652 | n.s. |
| Tmax_Nov | 7.692 | 4.000 | 0.055 | -0.830 | 0.399 | 0.037 | 70.055 | 66.055 | 70.905 | 4.849 | 0.028 | 18.265 |
| Tmax_Dec | 7.581 | 3.095 | 0.014 | -1.080 | 0.405 | 0.008 | 65.722 | 61.722 | 70.905 | 9.182 | 0.002 | 13.932 |
|  |  |  |  |  |  |  |  |  |  |  |  |  |
| Tmin_Jan | -2.815 | 3.092 | 0.363 | -0.148 | 0.209 | 0.479 | 74.400 | 70.400 | 70.905 | 0.505 | 0.478 | n.s. |
| Tmin_Feb | -7.122 | 3.977 | 0.073 | -0.465 | 0.283 | 0.100 | 71.958 | 67.958 | 70.905 | 2.946 | 0.086 | n.s. |
| Tmin_Mar | -6.405 | 1.664 | 1.2*10^-4^ | -1.324 | 0.375 | 4.2*10^-4^ | 54.756 | 50.756 | 70.905 | 20.149 | 7.2*10^-6^ | 2.966 |
| Tmin_Apr | -1.722 | 0.476 | 2.9*10^-4^ | -1.493 | 0.441 | 7.1*10^-4^ | 55.406 | 51.406 | 70.905 | 19.498 | 1.0*10^-5^ | 3.616 |
| Tmin_May | 3.136 | 1.351 | 0.020 | -1.219 | 0.424 | 0.004 | 63.993 | 59.993 | 70.905 | 10.912 | 9.6*10^-4^ | 12.203 |
| Tmin_Jun | 9.752 | 3.184 | 0.002 | -1.594 | 0.488 | 0.001 | 58.671 | 54.671 | 70.905 | 16.233 | 5.6*10^-5^ | 6.881 |
| Tmin_Jul | 15.387 | 4.765 | 0.001 | -1.798 | 0.535 | 7.7*10^-4^ | 56.376 | 52.376 | 70.905 | 18.528 | 1.7*10^-5^ | 4.586 |
| Tmin_Aug | 4.543 | 2.823 | 0.108 | -0.585 | 0.317 | 0.064 | 70.007 | 66.007 | 70.905 | 4.898 | 0.027 | n.s. |
| Tmin_Sep | 7.640 | 2.403 | 0.001 | -1.799 | 0.521 | 5.5*10^-4^ | 56.723 | 52.723 | 70.905 | 18.182 | 2.0*10^-5^ | 4.933 |
| Tmin_Oct | 0.930 | 0.591 | 0.116 | -1.523 | 0.475 | 0.001 | 60.318 | 56.318 | 70.905 | 14.586 | 1.3*10^-4^ | 8.528 |
| Tmin_Nov | -5.604 | 1.736 | 0.001 | -1.283 | 0.444 | 0.004 | 62.993 | 58.993 | 70.905 | 11.912 | 5.6*10^-4^ | 11.203 |
| Tmin_Dec | -14.910 | 3.831 | 1.0*10^-4^ | -1.927 | 0.513 | 1.7*10^-4^ | **51.790** | 47.790 | 70.905 | 23.114 | 1.5*10^-6^ | **0** |
|  |  |  |  |  |  |  |  |  |  |  |  |  |
| Tmax - Tmin_Jan | 1.170 | 3.946 | 0.767 | -0.083 | 0.181 | 0.646 | 74.691 | 70.691 | 70.905 | 0.213 | 0.644 | n.s. |
| Tmax - Tmin_Feb | 5.793 | 5.179 | 0.263 | -0.284 | 0.229 | 0.214 | 73.324 | 69.324 | 70.905 | 1.580 | 0.209 | n.s. |
| Tmax - Tmin_Mar | -18.713 | 8.566 | 0.029 | 1.011 | 0.479 | 0.035 | 69.515 | 65.515 | 70.905 | 5.390 | 0.020 | 17.725 |
| Tmax - Tmin_Apr | -31.594 | 11.063 | 0.004 | 1.678 | 0.600 | 0.005 | 63.83 | 59.830 | 70.905 | 11.074 | 8.8*10^-4^ | 12.040 |
| Tmax - Tmin_May | -6.675 | 8.600 | 0.438 | 0.316 | 0.449 | 0.482 | 74.404 | 70.404 | 70.905 | 0.500 | 0.479 | n.s. |
| Tmax - Tmin_Jun | 1.455 | 4.031 | 0.718 | -0.109 | 0.209 | 0.603 | 74.62 | 70.620 | 70.905 | 0.284 | 0.594 | n.s. |
| Tmax - Tmin_Jul | -11.524 | 6.552 | 0.079 | 0.614 | 0.369 | 0.096 | 71.909 | 67.909 | 70.905 | 2.995 | 0.084 | n.s. |
| Tmax - Tmin_Aug | -0.608 | 3.557 | 0.864 | -0.002 | 0.194 | 0.993 | 74.905 | 70.905 | 70.905 | 7.7*10^-5^ | 0.993 | n.s. |
| Tmax - Tmin_Sep | -2.607 | 8.099 | 0.748 | 0.100 | 0.412 | 0.808 | 74.846 | 70.846 | 70.905 | 0.059 | 0.808 | n.s. |
| Tmax - Tmin_Oct | -28.314 | 8.727 | 0.001 | 1.672 | 0.526 | 0.001 | 60.927 | 56.927 | 70.905 | 13.977 | 1.9*10^-4^ | 9.137 |
| Tmax - Tmin_Nov | -23.549 | 9.840 | 0.017 | 1.642 | 0.703 | 0.020 | 68.349 | 64.349 | 70.905 | 6.556 | 0.010 | 16.559 |
| Tmax - Tmin_Dec | -12.893 | 6.325 | 0.042 | 0.817 | 0.420 | 0.052 | 70.856 | 66.856 | 70.905 | 4.049 | 0.044 | n.s. |

**Table S6**. Sensitivity, specificity and correct classification rates of logistic regression models relating climatic variables and presences/absences of *T. helenitis* for the period 1900–1949 (Table S5). In this period, the number of presences is 19 and the number of absences is 36. Regressions were applied to predict the occurrence probability of each of the presences and absences, and then a threshold was used to classify a record as a predicted presence or absence. Shown are ΔAIC values of models, the threshold value (the predicted occurrence probability is 0.5 for this value), sensitivity (the percentage of correctly predicted presences), specificity (the percentage of correctly predicted absences) and the correct classification rate (CCR, the percentage of correctly predicted presences and absences). Blue: 2 ≤ ΔAIC < 10 (model selection, Burnham & Anderson, 2002). Red = the logistic regression was not significant (Table S5).

|  | ΔAIC | Threshold | Sensitivity (%) | Specifity (%) | CCR (%) |
| --- | --- | --- | --- | --- | --- |
| Precip_Jan | n.s. | 115.804 | 0.00 | 97.22 | 63.64 |
| Precip_Feb | n.s. | 86.504 | 10.53 | 97.22 | 67.27 |
| Precip_Mar | n.s. | 77.690 | 10.53 | 97.22 | 67.27 |
| Precip_Apr | n.s. | 79.883 | 15.79 | 97.22 | 69.09 |
| Precip_May | n.s. | 79.986 | 15.79 | 97.22 | 69.09 |
| Precip_Jun | n.s. | 118.492 | 0.00 | 100.00 | 65.45 |
| Precip_Jul | n.s. | 108.329 | 15.79 | 97.22 | 69.09 |
| Precip_Aug | n.s. | 124.793 | 0.00 | 100.00 | 65.45 |
| Precip_Sep | n.s. | 111.232 | 0.00 | 100.00 | 65.45 |
| Precip_Oct | n.s. | 103.367 | 5.26 | 97.22 | 65.45 |
| Precip_Nov | n.s. | 117.268 | 0.00 | 97.22 | 63.64 |
| Precip_Dec | n.s. | 104.131 | 15.79 | 97.22 | 69.09 |
| Precip_Year | n.s. | 1172.094 | 10.53 | 97.22 | 67.27 |
|  |  |  |  |  |  |
| Tmean_Jan | 11.275 | -1.021 | 36.84 | 97.22 | 76.36 |
| Tmean _Feb | 11.354 | -0.236 | 36.84 | 97.22 | 76.36 |
| Tmean _Mar | 11.055 | 3.026 | 36.84 | 91.67 | 72.73 |
| Tmean _Apr | 11.053 | 6.821 | 36.84 | 88.89 | 70.91 |
| Tmean _May | 10.852 | 11.661 | 36.84 | 91.67 | 72.73 |
| Tmean _Jun | 10.808 | 14.399 | 36.84 | 88.89 | 70.91 |
| Tmean _Jul | 11.264 | 16.130 | 36.84 | 91.67 | 72.73 |
| Tmean _Aug | 11.392 | 15.398 | 36.84 | 91.67 | 72.73 |
| Tmean _Sep | 12.069 | 12.378 | 36.84 | 91.67 | 72.73 |
| Tmean _Oct | 11.946 | 7.685 | 36.84 | 94.44 | 74.55 |
| Tmean _Nov | 11.756 | 2.905 | 36.84 | 97.22 | 76.36 |
| Tmean _Dec | 12.530 | 0.013 | 36.84 | 97.22 | 76.36 |
| Tmean _Year | 11.234 | 7.442 | 36.84 | 91.67 | 72.73 |
|  |  |  |  |  |  |
| Tmax_Jan | n.s. | 5.989 | 31.58 | 97.22 | 74.55 |
| Tmax _Feb | 13.612 | 7.989 | 42.11 | 86.11 | 70.91 |
| Tmax _Mar | 15.199 | 12.543 | 36.84 | 91.67 | 72.73 |
| Tmax _Apr | 17.863 | 16.734 | 31.58 | 91.67 | 70.91 |
| Tmax _May | 18.031 | 21.199 | 31.58 | 88.89 | 69.09 |
| Tmax _Jun | 15.515 | 24.660 | 31.58 | 88.89 | 69.09 |
| Tmax _Jul | n.s. | 25.424 | 31.58 | 94.44 | 72.73 |
| Tmax _Aug | 16.424 | 26.203 | 36.84 | 91.67 | 72.73 |
| Tmax _Sep | 14.884 | 23.428 | 36.84 | 91.67 | 72.73 |
| Tmax _Oct | n.s. | 13.357 | 0.00 | 100.00 | 65.45 |
| Tmax _Nov | 18.265 | 9.268 | 36.84 | 97.22 | 76.36 |
| Tmax _Dec | 13.932 | 7.019 | 36.84 | 91.67 | 72.73 |
|  |  |  |  |  |  |
| Tmin_Jan | n.s. | -18.988 | 0.00 | 100.00 | 65.45 |
| Tmin _Feb | n.s. | -15.318 | 21.05 | 97.22 | 70.91 |
| Tmin _Mar | 2.966 | -4.838 | 57.89 | 94.44 | 81.82 |
| Tmin _Apr | 3.616 | -1.153 | 52.63 | 91.67 | 78.18 |
| Tmin _May | 12.203 | 2.574 | 36.84 | 97.22 | 76.36 |
| Tmin _Jun | 6.881 | 6.117 | 52.63 | 88.89 | 76.36 |
| Tmin _Jul | 4.586 | 8.558 | 52.63 | 91.67 | 78.18 |
| Tmin _Aug | n.s. | 7.761 | 31.58 | 97.22 | 74.55 |
| Tmin _Sep | 4.933 | 4.247 | 57.89 | 91.67 | 80.00 |
| Tmin _Oct | 8.528 | 0.610 | 47.37 | 91.67 | 76.36 |
| Tmin _Nov | 11.203 | -4.367 | 36.84 | 94.44 | 74.55 |
| Tmin _Dec | **0** | -7.737 | **63.16** | 94.44 | **83.64** |
|  |  |  |  |  |  |
| Tmax - Tmin_Jan | n.s. | 14.051 | 0.00 | 100.00 | 65.45 |
| Tmax - Tmin_Feb | n.s. | 20.424 | 21.05 | 97.22 | 70.91 |
| Tmax - Tmin_Mar | 17.725 | 18.505 | 26.32 | 94.44 | 70.91 |
| Tmax - Tmin_Apr | 12.040 | 18.825 | 36.84 | 91.67 | 72.73 |
| Tmax - Tmin_May | n.s. | 21.156 | 0.00 | 100.00 | 65.45 |
| Tmax - Tmin_Jun | n.s. | 13.394 | 0.00 | 100.00 | 65.45 |
| Tmax - Tmin_Jul | n.s. | 18.757 | 15.79 | 94.44 | 67.27 |
| Tmax - Tmin_Aug | n.s. | -357.608 | 0.00 | 100.00 | 65.45 |
| Tmax - Tmin_Sep | n.s. | 26.002 | 0.00 | 100.00 | 65.45 |
| Tmax - Tmin_Oct | 9.137 | 16.933 | 52.63 | 91.67 | 78.18 |
| Tmax - Tmin_Nov | 16.559 | 14.345 | 36.84 | 88.89 | 70.91 |
| Tmax - Tmin_Dec | n.s. | 15.781 | 31.58 | 94.44 | 72.73 |

**Figure S5**. Results of the logistic regression analysis of the relation between climatic variables and presences/absences of *T. helenitis* for the period 1950–1979. A) Monthly precipitation sums, B) monthly mean temperatures, C) monthly maximum temperatures, D) monthly minimum temperatures, E) monthly temperature ranges (maximum – minimum monthly temperature), F) annual precipitation sum and annual mean temperature. Shown are presences (N=10) and absences (N=45) used to establish models, the logistic regression (logit) curve fitted, the threshold of the model (i.e., the value of the climatic variable for which the model predicts an occurrence probability of 0.5, dotted line) as well as the model’s ΔAIC value for goodness of fit or n.s. when the fit was not significant. For detailed statistics and goodness of fit of models see Tables S7 and S8.

A)


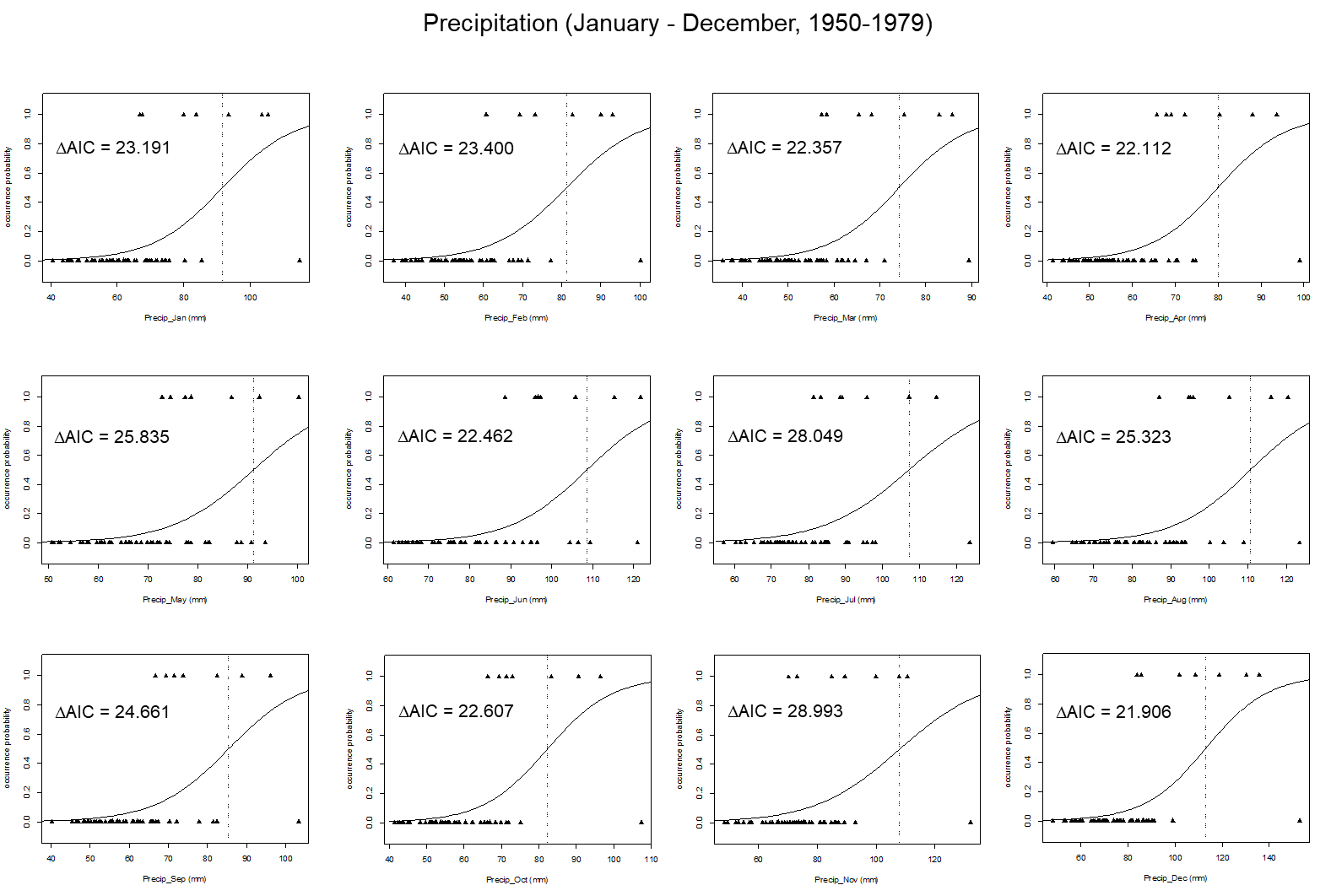


B)


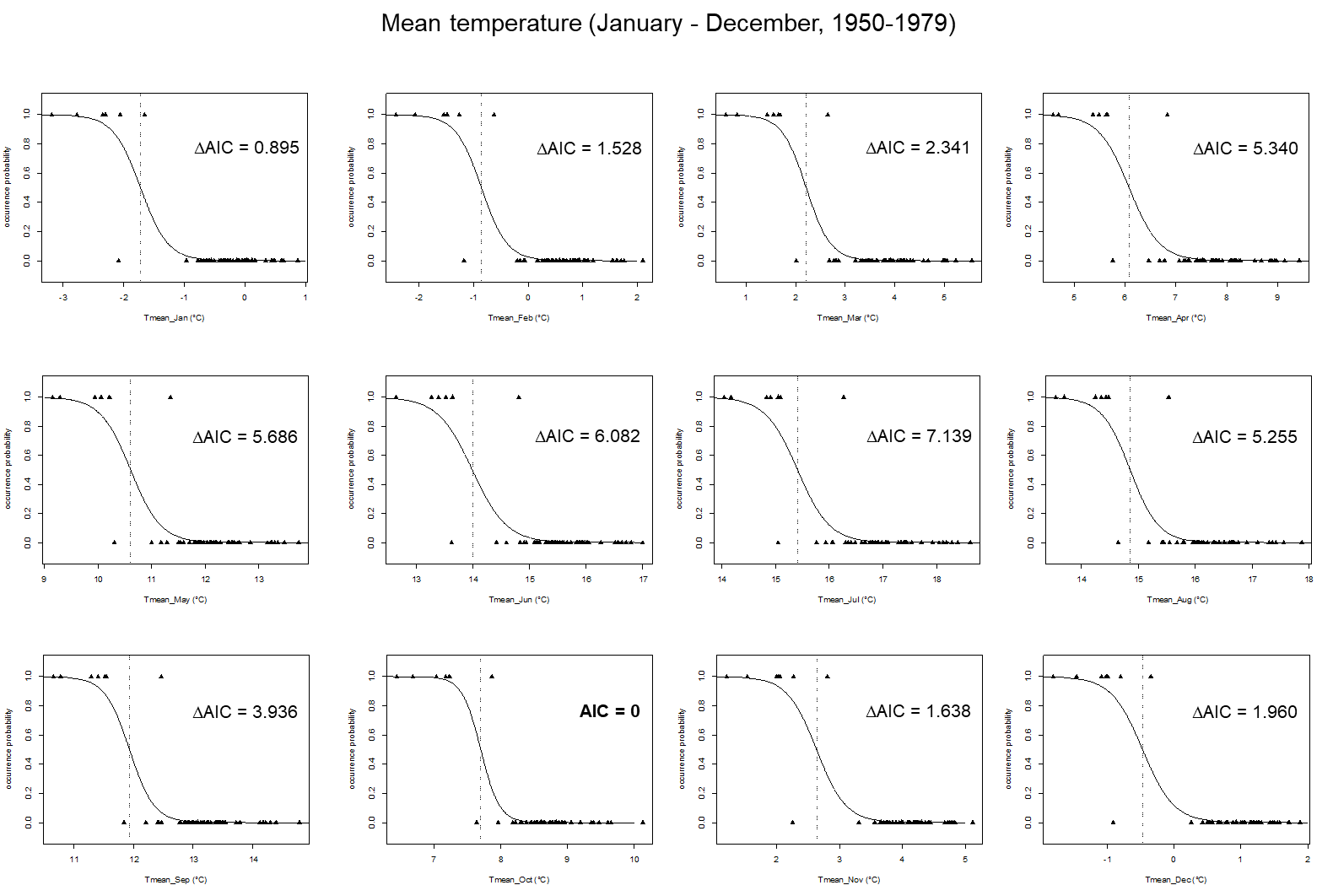


C)


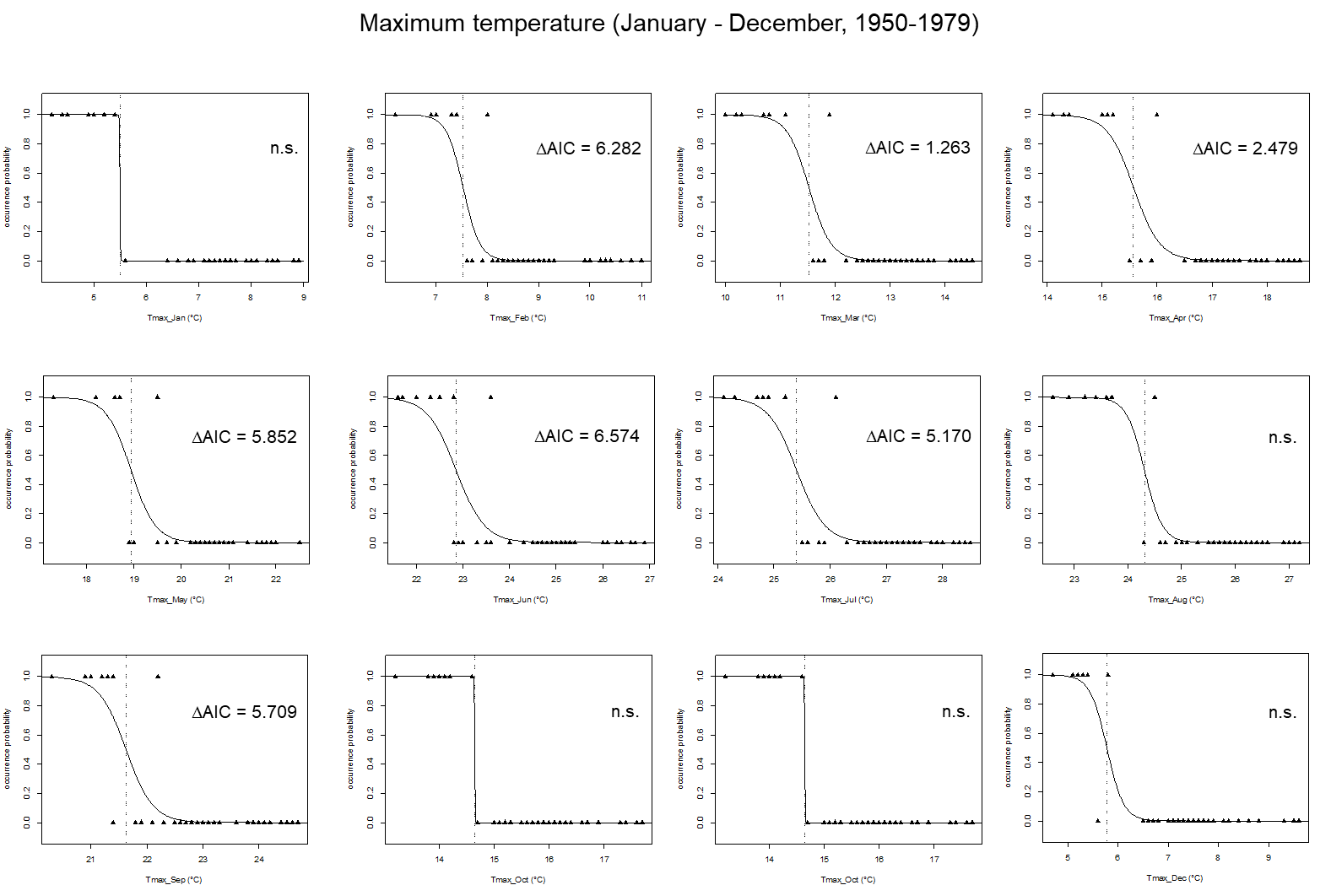


D)


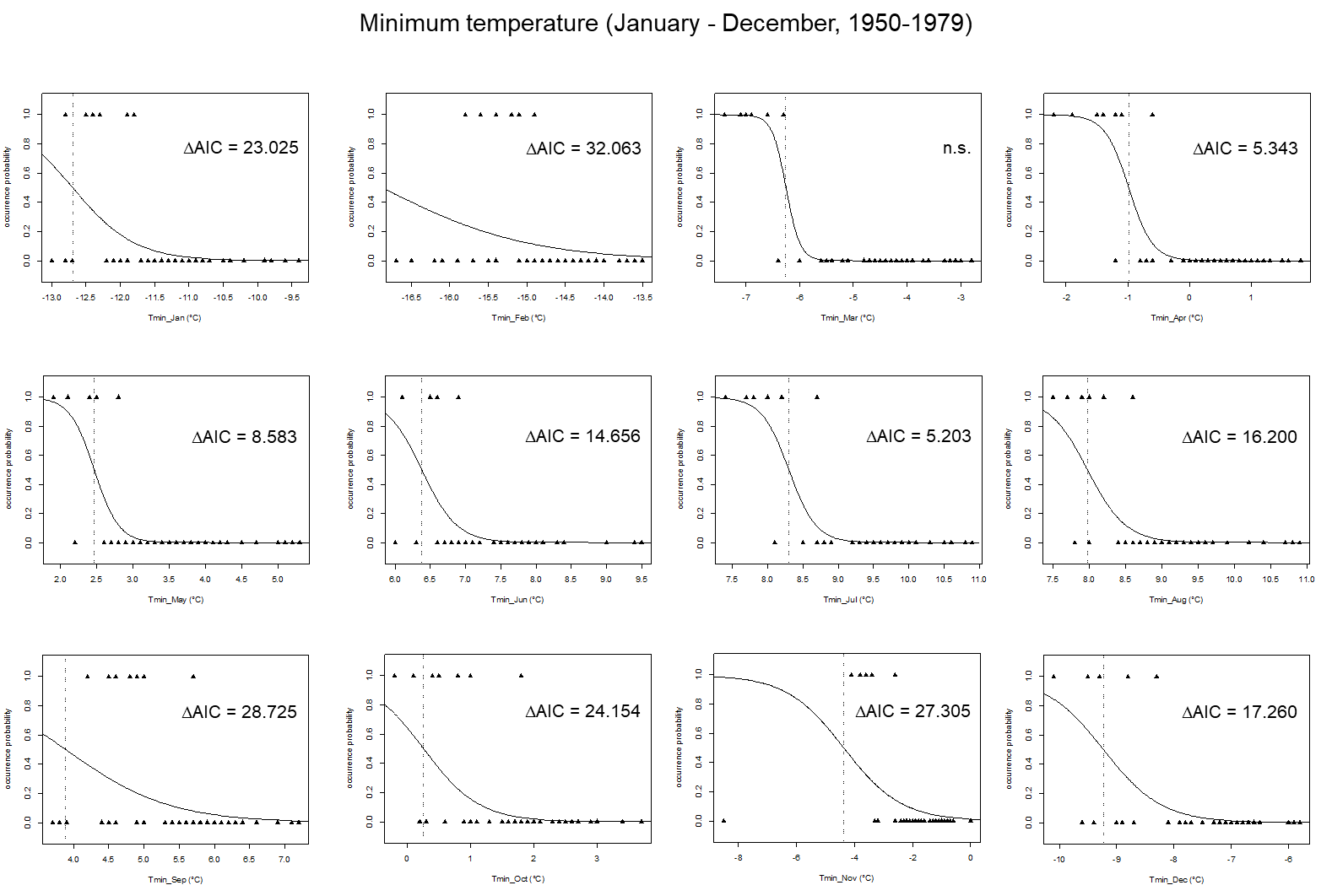


E)


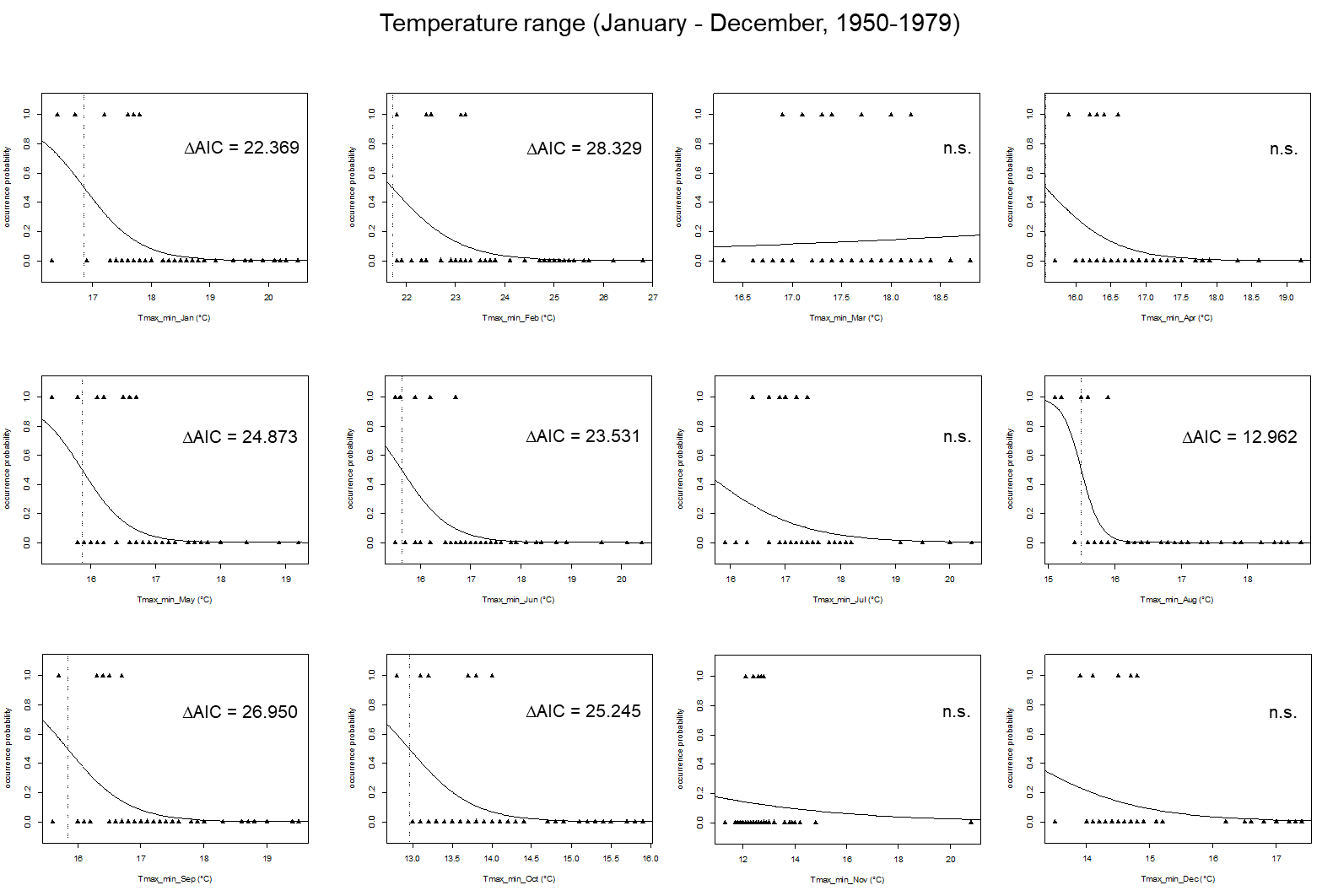


F)


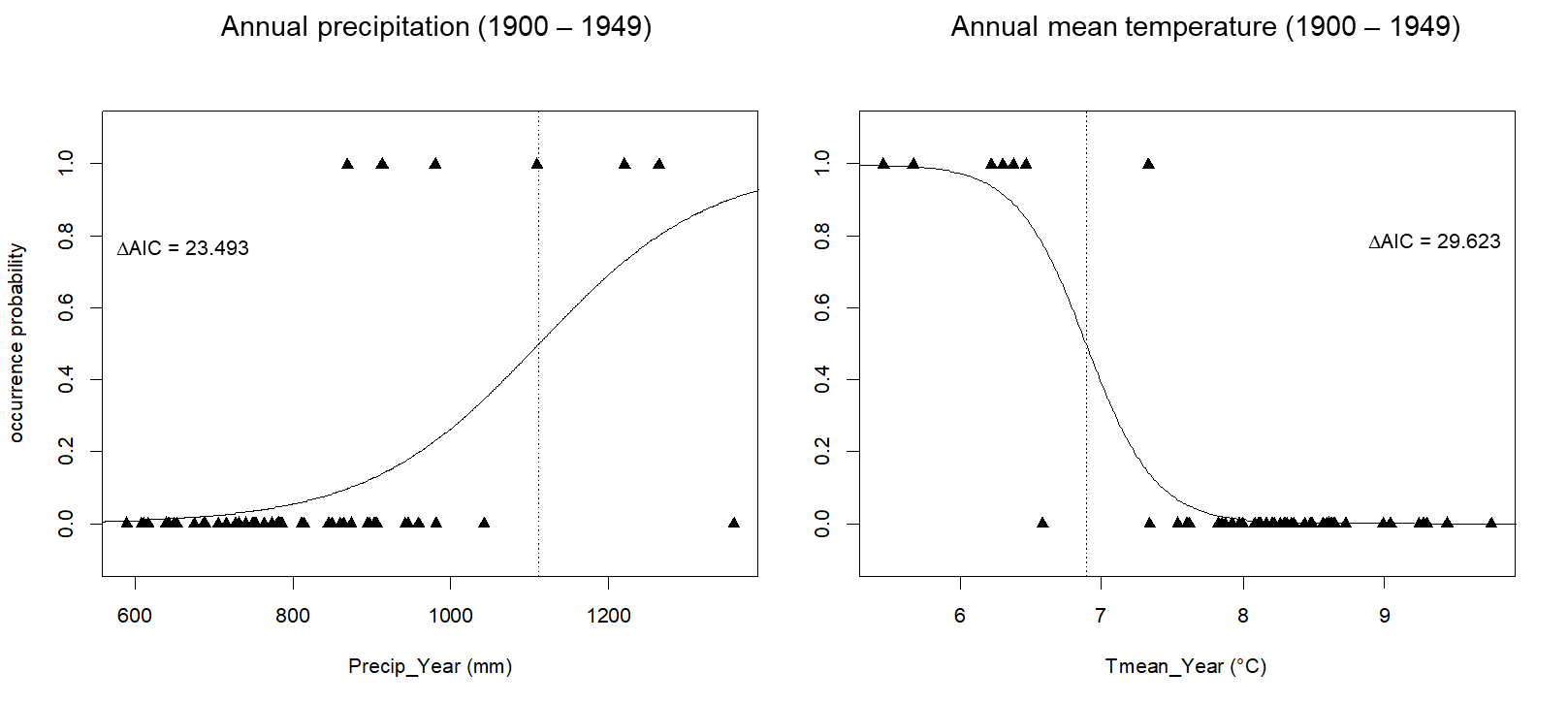


**Table S7**. Results of the logistic regression analysis of the relation between climatic variables and presences/absences of *T. helenitis* for the period 1950–1979. Shown are the intercepts and predictors (slope) with their standard errors (s.e.) and significance tests (p), as well as AIC and ΔAIC values. Residual deviance, null deviance, LRT and p are the results of the respective likelihood-ratio test. Only models with significant predictors and likelihood-ratio tests were passed to AIC-based model selection. Red = non-significant p-value or ΔAIC value, respectively. Blue = 2 ≤ ΔAIC < 10 (model selection, Burnham & Anderson, 2002). The number of presences is 10 and the number of absences 45.

|  | intercept | s.e. | p | predictor | s.e. | p | AIC | Residual  deviance | Null  deviance | LRT | p | ΔAIC |
| --- | --- | --- | --- | --- | --- | --- | --- | --- | --- | --- | --- | --- |
| Precip_Jan | -8.727 | 2.491 | 4.5*10^-4^ | 0.095 | 0.032 | 0.003 | 32.714 | 28.714 | 41.929 | 13.214 | 2.8*10^-4^ | 23.191 |
| Precip_Feb | -8.820 | 2.518 | 4.6*10^-4^ | 0.109 | 0.037 | 0.003 | 32.923 | 28.923 | 41.929 | 13.005 | 3.1*10^-4^ | 23.400 |
| Precip_Mar | -9.866 | 2.863 | 5.6*10^-4^ | 0.133 | 0.045 | 0.003 | 31.880 | 27.880 | 41.929 | 14.049 | 1.8*10^-4^ | 22.357 |
| Precip_Apr | -10.264 | 3.040 | 7.4*10^-4^ | 0.128 | 0.044 | 0.003 | 31.635 | 27.635 | 41.929 | 14.293 | 1.6*10^-4^ | 22.112 |
| Precip_May | -11.136 | 3.531 | 0.002 | 0.122 | 0.044 | 0.005 | 35.358 | 31.358 | 41.929 | 10.571 | 0.001 | 25.835 |
| Precip_Jun | -11.588 | 3.433 | 7.4*10^-4^ | 0.107 | 0.035 | 0.002 | 31.985 | 27.985 | 41.929 | 13.943 | 1.9*10^-4^ | 22.462 |
| Precip_Jul | -9.368 | 3.058 | 0.002 | 0.088 | 0.034 | 0.010 | 37.572 | 33.572 | 41.929 | 8.3564 | 0.004 | 28.049 |
| Precip_Aug | -11.227 | 3.477 | 0.001 | 0.102 | 0.036 | 0.004 | 34.846 | 30.846 | 41.929 | 11.082 | 8.7*10^-4^ | 25.323 |
| Precip_Sep | -9.217 | 2.726 | 7.2*10^-4^ | 0.108 | 0.038 | 0.004 | 34.184 | 30.184 | 41.929 | 11.745 | 6.1*10^-4^ | 24.661 |
| Precip_Oct | -9.549 | 2.840 | 7.7*10^-4^ | 0.116 | 0.041 | 0.004 | 32.130 | 28.130 | 41.929 | 13.798 | 2.0*10^-4^ | 22.607 |
| Precip_Nov | -7.571 | 2.489 | 0.002 | 0.070 | 0.029 | 0.017 | 38.516 | 34.516 | 41.929 | 7.413 | 0.006 | 28.993 |
| Precip_Dec | -8.648 | 2.430 | 3.7*10^-4^ | 0.076 | 0.026 | 0.003 | 31.429 | 27.429 | 41.929 | 14.499 | 1.4*10^-4^ | 21.906 |
| Precip_Year | -10.173 | 3.040 | 8.2*10^-4^ | 0.009 | 0.003 | 0.004 | 33.016 | 29.016 | 41.929 | 12.913 | 3.3*10^-4^ | 23.493 |
|  |  |  |  |  |  |  |  |  |  |  |  |  |
| Tmean_Jan | -7.676 | 3.540 | 0.030 | -4.461 | 1.886 | 0.018 | 10.418 | 6.418 | 41.929 | 35.511 | 2.5*10^-9^ | 0.895 |
| Tmean_Feb | -3.673 | 1.676 | 0.028 | -4.285 | 1.657 | 0.010 | 11.051 | 7.051 | 41.929 | 34.878 | 3.5*10^-9^ | 1.528 |
| Tmean_Mar | 9.073 | 3.896 | 0.020 | -4.097 | 1.598 | 0.010 | 11.864 | 7.864 | 41.929 | 34.065 | 5.3*10^-9^ | 2.341 |
| Tmean_Apr | 20.451 | 7.229 | 0.005 | -3.363 | 1.143 | 0.003 | 14.863 | 10.863 | 41.929 | 31.066 | 2.5*10^-8^ | 5.340 |
| Tmean_May | 37.840 | 12.970 | 0.004 | -3.566 | 1.196 | 0.003 | 15.209 | 11.209 | 41.929 | 30.720 | 3.0*10^-8^ | 5.686 |
| Tmean_Jun | 46.206 | 15.202 | 0.002 | -3.303 | 1.066 | 0.002 | 15.605 | 11.605 | 41.929 | 30.323 | 3.7*10^-8^ | 6.082 |
| Tmean_Jul | 51.845 | 17.265 | 0.003 | -3.363 | 1.103 | 0.002 | 16.662 | 12.662 | 41.929 | 29.267 | 6.3*10^-8^ | 7.139 |
| Tmean_Aug | 59.601 | 20.587 | 0.004 | -4.011 | 1.368 | 0.003 | 14.778 | 10.778 | 41.929 | 31.151 | 2.4*10^-8^ | 5.255 |
| Tmean_Sep | 57.891 | 21.539 | 0.007 | -4.853 | 1.779 | 0.006 | 13.459 | 9.459 | 41.929 | 32.469 | 1.2*10^-8^ | 3.936 |
| Tmean_Oct | 55.341 | 26.607 | 0.038 | -7.190 | 3.408 | 0.035 | **9.523** | 5.523 | 41.929 | 36.406 | 1.6*10^-9^ | **0** |
| Tmean_Nov | 11.346 | 4.431 | 0.010 | -4.308 | 1.611 | 0.008 | 11.161 | 7.161 | 41.929 | 34.768 | 3.7*10^-9^ | 1.638 |
| Tmean_Dec | -2.024 | 1.246 | 0.104 | -4.284 | 1.586 | 0.007 | 11.483 | 7.483 | 41.929 | 34.446 | 4.4*10^-9^ | 1.960 |
| Tmean_Year | 28.069 | 10.085 | 0.005 | -4.075 | 1.424 | 0.004 | 12.809 | 8.809 | 41.929 | 33.120 | 8.7*10^-9^ | 29.623 |
|  |  |  |  |  |  |  |  |  |  |  |  |  |
| Tmax_Jan | 1035.700 | 290317.200 | 0.997 | -188.300 | 52892.000 | 0.997 | 4.000 | 0.000 | 41.929 | 41.929 | 9.0*10^-11^ | n.s. |
| Tmax_Feb | 46.479 | 21.794 | 0.033 | -6.176 | 2.869 | 0.031 | 15.805 | 11.805 | 41.929 | 30.124 | 4.1*10^-8^ | 6.282 |
| Tmax_Mar | 58.078 | 29.393 | 0.048 | -5.039 | 2.510 | 0.045 | 10.786 | 6.786 | 41.929 | 35.142 | 3.1*10^-9^ | 1.263 |
| Tmax_Apr | 65.572 | 29.937 | 0.029 | -4.210 | 1.915 | 0.028 | 12.002 | 8.002 | 41.929 | 33.926 | 5.7*10^-9^ | 2.479 |
| Tmax_May | 73.697 | 30.868 | 0.017 | -3.891 | 1.627 | 0.017 | 15.375 | 11.375 | 41.929 | 30.553 | 3.2*10^-8^ | 5.852 |
| Tmax_Jun | 74.732 | 31.857 | 0.019 | -3.270 | 1.388 | 0.019 | 16.097 | 12.097 | 41.929 | 29.832 | 4.7*10^-8^ | 6.574 |
| Tmax_Jul | 100.780 | 41.537 | 0.015 | -3.968 | 1.627 | 0.015 | 14.693 | 10.693 | 41.929 | 31.236 | 2.3*10^-8^ | 5.170 |
| Tmax_Aug | 145.456 | 95.349 | 0.127 | -5.984 | 3.898 | 0.125 | 8.997 | 4.997 | 41.929 | 36.932 | 1.2*10^-9^ | n.s. |
| Tmax_Sep | 90.357 | 32.948 | 0.006 | -4.177 | 1.517 | 0.006 | 15.232 | 11.232 | 41.929 | 30.697 | 3.0*10^-8^ | 5.709 |
| Tmax_Oct | 5377.800 | 1216291.400 | 0.996 | -367.100 | 83010.500 | 0.996 | 4.000 | 0.000 | 41.929 | 41.929 | 9.5*10^-11^ | n.s. |
| Tmax_Nov | 1758.400 | 602358.400 | 0.998 | -185.100 | 63406.100 | 0.998 | 6.773 | 2.773 | 41.929 | 39.156 | 3.9*10^-10^ | n.s. |
| Tmax_Dec | 34.636 | 21.640 | 0.109 | -5.993 | 3.844 | 0.119 | 8.830 | 4.830 | 41.929 | 37.098 | 1.1*10^-9^ | n.s. |
|  |  |  |  |  |  |  |  |  |  |  |  |  |
| Tmin_Jan | -27.944 | 9.657 | 0.004 | -2.201 | 0.792 | 0.006 | 32.548 | 28.548 | 41.929 | 13.381 | 2.5*10^-4^ | 23.025 |
| Tmin_Feb | -17.720 | 7.950 | 0.026 | -1.050 | 0.520 | 0.044 | 41.586 | 37.586 | 41.929 | 4.3426 | 0.037 | 32.063 |
| Tmin_Mar | -48.204 | 35.680 | 0.177 | -7.709 | 5.661 | 0.173 | 9.389 | 5.389 | 41.929 | 36.539 | 1.5*10^-9^ | n.s. |
| Tmin_Apr | -5.129 | 2.111 | 0.015 | -5.208 | 2.175 | 0.017 | 14.866 | 10.866 | 41.929 | 31.063 | 2.5*10^-8^ | 5.343 |
| Tmin_May | 14.728 | 5.760 | 0.011 | -5.970 | 2.232 | 0.008 | 18.106 | 14.106 | 41.929 | 27.822 | 1.3*10^-7^ | 8.583 |
| Tmin_Jun | 25.557 | 9.807 | 0.009 | -4.006 | 1.497 | 0.008 | 24.179 | 20.179 | 41.929 | 21.750 | 3.1*10^-6^ | 14.656 |
| Tmin_Jul | 43.095 | 17.204 | 0.012 | -5.191 | 2.053 | 0.012 | 14.726 | 10.726 | 41.929 | 31.203 | 2.3*10^-8^ | 5.203 |
| Tmin_Aug | 30.303 | 10.798 | 0.005 | -3.798 | 1.320 | 0.004 | 25.723 | 21.723 | 41.929 | 20.205 | 7.0*10^-6^ | 16.200 |
| Tmin_Sep | 5.290 | 2.745 | 0.054 | -1.360 | 0.541 | 0.012 | 38.248 | 34.248 | 41.929 | 7.681 | 0.006 | 28.725 |
| Tmin_Oct | 0.597 | 0.830 | 0.472 | -2.282 | 0.855 | 0.008 | 33.677 | 29.677 | 41.929 | 12.252 | 4.6*10^-4^ | 24.154 |
| Tmin_Nov | -4.408 | 1.273 | 5.3*10^-4^ | -1.010 | 0.448 | 0.024 | 36.828 | 32.828 | 41.929 | 9.1011 | 0.003 | 27.305 |
| Tmin_Dec | -18.125 | 5.777 | 0.002 | -1.964 | 0.652 | 0.003 | 26.783 | 22.783 | 41.929 | 19.145 | 1.2*10^-5^ | 17.260 |
|  |  |  |  |  |  |  |  |  |  |  |  |  |
| Tmax - Tmin_Jan | 35.801 | 13.076 | 0.006 | -2.124 | 0.750 | 0.005 | 31.892 | 27.892 | 41.929 | 14.036 | 1.8*10^-4^ | 22.369 |
| Tmax - Tmin_Feb | 32.048 | 15.268 | 0.036 | -1.476 | 0.673 | 0.028 | 37.852 | 33.852 | 41.929 | 8.076 | 0.004 | 28.329 |
| Tmax - Tmin_Mar | -6.599 | 12.804 | 0.606 | 0.268 | 0.731 | 0.714 | 45.796 | 41.796 | 41.929 | 0.133 | 0.716 | n.s. |
| Tmax - Tmin_Apr | 31.619 | 17.744 | 0.075 | -2.031 | 1.086 | 0.062 | 40.170 | 36.170 | 41.929 | 5.759 | 0.016 | n.s. |
| Tmax - Tmin_May | 44.351 | 16.934 | 0.009 | -2.794 | 1.035 | 0.007 | 34.360 | 30.360 | 41.929 | 11.569 | 6.7*10^-4^ | 24.873 |
| Tmax - Tmin_Jun | 32.834 | 12.273 | 0.008 | -2.100 | 0.758 | 0.006 | 33.054 | 29.054 | 41.929 | 12.875 | 3.3*10^-4^ | 23.531 |
| Tmax - Tmin_Jul | 17.543 | 11.786 | 0.137 | -1.134 | 0.693 | 0.102 | 42.670 | 38.670 | 41.929 | 3.259 | 0.071 | n.s. |
| Tmax - Tmin_Aug | 107.760 | 46.706 | 0.021 | -6.952 | 2.994 | 0.020 | 22.485 | 18.485 | 41.929 | 23.444 | 1.3*10^-6^ | 12.962 |
| Tmax - Tmin_Sep | 33.134 | 14.418 | 0.022 | -2.092 | 0.874 | 0.017 | 36.473 | 32.473 | 41.929 | 9.456 | 0.002 | 26.950 |
| Tmax - Tmin_Oct | 32.686 | 13.942 | 0.019 | -2.522 | 1.036 | 0.015 | 34.768 | 30.768 | 41.929 | 11.161 | 8.4*10^-4^ | 25.245 |
| Tmax - Tmin_Nov | 1.047 | 6.385 | 0.870 | -0.236 | 0.510 | 0.643 | 45.630 | 41.630 | 41.929 | 0.298 | 0.585 | n.s. |
| Tmax - Tmin_Dec | 13.216 | 12.092 | 0.274 | -1.037 | 0.837 | 0.215 | 43.484 | 39.484 | 41.929 | 2.445 | 0.118 | n.s. |

**Table S8**. Sensitivity, specificity and correct classification rates of logistic regression models relating climatic variables and presences/absences of *T. helenitis* for the period 1950–1979 (Table S6). In this period, the number of presences is 10 and the number of absences is 45. Regressions were applied to predict the occurrence probability of each of these presences and absences, and a threshold was used to classify a record as a predicted presence or absence. Shown are ΔAIC values of models, the threshold value (the predicted occurrence probability is 0.5 for this value), sensitivity (the percentage of correctly predicted presences), specificity (the percentage of correctly predicted absences) and the correct classification rate (CCR, the percentage of correctly predicted presences and absences). Blue = 2 ≤ ΔAIC < 10 (model selection, Burnham & Anderson, 2002). Red = the logistic regression was not significant (Table S7).

|  | ΔAIC | Threshold | Sensitivity (%) | Specifity (%) | CCR (%) |
| --- | --- | --- | --- | --- | --- |
| Precip_Jan | 23.191 | 91.649 | 30.00 | 97.78 | 85.45 |
| Precip_Feb | 23.400 | 81.205 | 30.00 | 97.78 | 85.45 |
| Precip_Mar | 22.357 | 74.164 | 30.00 | 97.78 | 85.45 |
| Precip_Apr | 22.112 | 79.970 | 30.00 | 97.78 | 85.45 |
| Precip_May | 25.835 | 91.195 | 20.00 | 97.78 | 83.64 |
| Precip_Jun | 22.462 | 108.625 | 20.00 | 95.56 | 81.18 |
| Precip_Jul | 28.049 | 107.072 | 20.00 | 97.78 | 83.64 |
| Precip_Aug | 25.323 | 110.598 | 20.00 | 97.78 | 83.64 |
| Precip_Sep | 24.661 | 85.462 | 20.00 | 97.78 | 83.64 |
| Precip_Oct | 22.607 | 82.180 | 30.00 | 97.78 | 85.45 |
| Precip_Nov | 28.993 | 107.875 | 10.00 | 97.78 | 81.82 |
| Precip_Dec | 21.906 | 113.301 | 30.00 | 97.78 | 85.45 |
| Precip_Year | 23.493 | 1112.332 | 20.00 | 97.78 | 83.64 |
|  |  |  |  |  |  |
| Tmean_Jan | 0.895 | -1.721 | 60.00 | 97.78 | 90.91 |
| Tmean _Feb | 1.528 | -0.857 | 60.00 | 97.78 | 90.91 |
| Tmean _Mar | 2.341 | 2.214 | 60.00 | 97.78 | 90.91 |
| Tmean _Apr | 5.340 | 6.081 | 60.00 | 97.78 | 90.91 |
| Tmean _May | 5.686 | 10.611 | 60.00 | 97.78 | 90.91 |
| Tmean _Jun | 6.082 | 13.990 | 60.00 | 97.78 | 90.91 |
| Tmean _Jul | 7.139 | 15.415 | 60.00 | 97.78 | 90.91 |
| Tmean _Aug | 5.255 | 14.859 | 60.00 | 97.78 | 90.91 |
| Tmean _Sep | 3.936 | 11.930 | 60.00 | 97.78 | 90.91 |
| Tmean _Oct | **0** | 7.697 | 60.00 | 97.78 | 90.91 |
| Tmean _Nov | 1.638 | 2.634 | 60.00 | 97.78 | 90.91 |
| Tmean _Dec | 1.960 | -0.473 | 60.00 | 97.78 | 90.91 |
| Tmean _Year | 29.623 | 3.759 | 60.00 | 97.78 | 90.91 |
|  |  |  |  |  |  |
| Tmax_Jan | n.s. | 5.499 | 70.00 | 100.00 | 94.54 |
| Tmax _Feb | 6.282 | 7.526 | 60.00 | 100.00 | 92.72 |
| Tmax _Mar | 1.263 | 11.526 | 60.00 | 100.00 | 92.72 |
| Tmax _Apr | 2.479 | 15.575 | 60.00 | 97.78 | 90.91 |
| Tmax _May | 5.852 | 19.941 | 70.00 | 95.56 | 90.91 |
| Tmax _Jun | 6.574 | 22.852 | 60.00 | 97.78 | 90.91 |
| Tmax _Jul | 5.170 | 25.401 | 60.00 | 100.00 | 92.72 |
| Tmax _Aug | n.s. | 24.307 | 60.00 | 97.78 | 90.91 |
| Tmax _Sep | 5.709 | 21.631 | 60.00 | 97.78 | 90.91 |
| Tmax _Oct | n.s. | 14.650 | 70.00 | 100.00 | 94.54 |
| Tmax _Nov | n.s. | 9.500 | 60.00 | 100.00 | 92.72 |
| Tmax _Dec | n.s. | 5.780 | 60.00 | 97.78 | 90.91 |
|  |  |  |  |  |  |
| Tmin_Jan | 23.025 | -12.694 | 30.00 | 84.44 | 74.55 |
| Tmin _Feb | 32.063 | -16.880 | 0.00 | 100.00 | 81.82 |
| Tmin _Mar | n.s. | -6.253 | 70.00 | 97.78 | 92.72 |
| Tmin _Apr | 5.343 | -0.985 | 60.00 | 97.78 | 90.91 |
| Tmin _May | 8.583 | 2.467 | 50.00 | 97.78 | 89.09 |
| Tmin _Jun | 14.656 | 6.380 | 40.00 | 95.56 | 85.45 |
| Tmin _Jul | 5.203 | 8.301 | 60.00 | 97.78 | 90.91 |
| Tmin _Aug | 16.200 | 7.979 | 40.00 | 95.56 | 85.45 |
| Tmin _Sep | 28.725 | 3.891 | 10.00 | 95.56 | 80.00 |
| Tmin _Oct | 24.154 | 0.262 | 20.00 | 97.78 | 83.64 |
| Tmin _Nov | 27.305 | -4.363 | 0.00 | 97.78 | 80.00 |
| Tmin _Dec | 17.260 | -9.228 | 50.00 | 93.33 | 85.45 |
|  |  |  |  |  |  |
| Tmax - Tmin_Jan | 22.369 | 16.859 | 30.00 | 97.78 | 85.45 |
| Tmax - Tmin_Feb | 28.329 | 21.713 | 0.00 | 100.00 | 81.82 |
| Tmax - Tmin_Mar | n.s. | 24.671 | 30.00 | 100.00 | 87.27 |
| Tmax - Tmin_Apr | n.s. | 15.572 | 0.00 | 100.00 | 81.82 |
| Tmax - Tmin_May | 24.873 | 15.872 | 20.00 | 97.78 | 83.64 |
| Tmax - Tmin_Jun | 23.531 | 15.633 | 40.00 | 97.78 | 87.27 |
| Tmax - Tmin_Jul | n.s. | 15.471 | 0.00 | 100.00 | 81.82 |
| Tmax - Tmin_Aug | 12.962 | 15.501 | 50.00 | 97.78 | 89.09 |
| Tmax - Tmin_Sep | 26.950 | 15.841 | 10.00 | 97.78 | 81.82 |
| Tmax - Tmin_Oct | 25.245 | 12.960 | 20.00 | 100.00 | 85.45 |
| Tmax - Tmin_Nov | n.s. | 4.432 | 0.00 | 100.00 | 81.82 |
| Tmax - Tmin_Dec | n.s. | 12.747 | 0.00 | 100.00 | 81.82 |

**Table S9**. Application of the logistic regression models established for the periods 1900–1949 and 1950–1979, respectively, to 2000–2020. In 2000–2020, *T. helenitis* was recorded in three QTK25s (47,20; 47,26; 55,26) in Hessia. Conditions: value of the variable for each of the three QTK25s; p(,) = occurrence probability calculated from conditions by the model for each QTK25; sensitivity (sens.) and specificity (spec.): numbers of correctly predicted presences and absences in the study area of *T. helenitis*, an occurrence probability of 0.5 is used as threshold. Blue: 2 ≤ ΔAIC < 10 (model selection, Burnham & Anderson, 2002). Red = the logistic regression was not significant (Tables S5 and S7).

|  | Conditions in 2000–2020 | | | Models on 1900–1949 | | | | | | Models 1950–1979 | | | | | |
| --- | --- | --- | --- | --- | --- | --- | --- | --- | --- | --- | --- | --- | --- | --- | --- |
|  | (47,20) | (47,26) | (55,26) | ΔAIC | p_(47,20)_ | p_(47,26)_ | p_(55,26)_ | sens. | spec. | ΔAIC | p_(47,20)_ | p_(47,26)_ | p_(55,26)_ | sens. | spec. |
| Precip_Jan | 61.1 | 84.8 | 120.2 | n.s. | 0.329 | 0.400 | 0.514 | 1 | 50 | 23.191 | 0.052 | 0.341 | 0.938 | 1 | 47 |
| Precip_Feb | 42.3 | 64.0 | 83.1 | n.s. | 0.286 | 0.386 | 0.482 | 0 | 50 | 23.400 | 0.014 | 0.134 | 0.551 | 1 | 50 |
| Precip_Mar | 50.3 | 79.4 | 100.6 | n.s. | 0.340 | 0.510 | 0.635 | 2 | 41 | 22.357 | 0.040 | 0.667 | 0.971 | 2 | 33 |
| Precip_Apr | 45.6 | 70.3 | 82.1 | n.s. | 0.255 | 0.426 | 0.517 | 1 | 50 | 22.112 | 0.012 | 0.224 | 0.567 | 1 | 50 |
| Precip_May | 63.7 | 81.2 | 86.3 | n.s. | 0.339 | 0.513 | 0.565 | 2 | 43 | 25.835 | 0.034 | 0.229 | 0.356 | 0 | 48 |
| Precip_Jun | 62.9 | 93.0 | 103.0 | n.s. | 0.316 | 0.412 | 0.446 | 0 | 51 | 22.462 | 0.008 | 0.158 | 0.354 | 0 | 50 |
| Precip_Jul | 59.9 | 92.9 | 111.4 | n.s. | 0.231 | 0.405 | 0.519 | 1 | 50 | 28.049 | 0.016 | 0.224 | 0.594 | 1 | 50 |
| Precip_Aug | 52.2 | 77.0 | 77.9 | n.s. | 0.260 | 0.335 | 0.337 | 0 | 52 | 25.323 | 0.003 | 0.032 | 0.035 | 0 | 52 |
| Precip_Sep | 63.2 | 82.5 | 94.0 | n.s. | 0.349 | 0.408 | 0.444 | 0 | 51 | 24.661 | 0.083 | 0.423 | 0.715 | 1 | 47 |
| Precip_Oct | 57.7 | 76. 6 | 105.3 | n.s. | 0.306 | 0.382 | 0.509 | 1 | 50 | 22.607 | 0.055 | 0.342 | 0.937 | 1 | 33 |
| Precip_Nov | 55.5 | 80.9 | 101.8 | n.s. | 0.313 | 0.387 | 0.451 | 0 | 51 | 28.993 | 0.025 | 0.131 | 0.394 | 0 | 50 |
| Precip_Dec | 65.5 | 99.6 | 136.6 | n.s. | 0.332 | 0.480 | 0.643 | 1 | 47 | 21.906 | 0.025 | 0.260 | 0.856 | 1 | 50 |
| Precip_Year | 679.9 | 982.2 | 1202.3 | n.s. | 0.301 | 0.420 | 0.513 | 1 | 50 | 23.493 | 0.019 | 0.233 | 0.695 | 1 | 50 |
|  |  |  |  |  |  |  |  |  |  |  |  |  |  |  |  |
| Tmean_Jan | -0.3 | -0.3 | -2.4 | 11.275 | 0.275 | 0.283 | 0.852 | 1 | 47 | 0.895 | 0.001 | 0.002 | 0.953 | 1 | 51 |
| Tmean _Feb | 0.1 | -0.1 | -2.2 | 11.354 | 0.403 | 0.460 | 0.909 | 1 | 47 | 1.528 | 0.016 | 0.037 | 0.997 | 1 | 47 |
| Tmean _Mar | 3.9 | 3.5 | 1.3 | 11.055 | 0.294 | 0.365 | 0.863 | 1 | 47 | 2.341 | 0.001 | 0.004 | 0.978 | 1 | 50 |
| Tmean _Apr | 7.2 | 6.9 | 4.9 | 11.053 | 0.402 | 0.471 | 0.873 | 1 | 46 | 5.340 | 0.021 | 0.053 | 0.981 | 1 | 47 |
| Tmean _May | 11.9 | 11.7 | 9.8 | 10.852 | 0.436 | 0.482 | 0.887 | 1 | 45 | 5.686 | 0.010 | 0.018 | 0.950 | 1 | 51 |
| Tmean _Jun | 14.4 | 14.4 | 12.4 | 10.808 | 0.497 | 0.512 | 0.900 | 2 | 43 | 6.082 | 0.199 | 0.231 | 0.996 | 1 | 46 |
| Tmean _Jul | 16.9 | 16.6 | 14.8 | 11.264 | 0.318 | 0.384 | 0.807 | 1 | 47 | 7.139 | 0.008 | 0.019 | 0.900 | 1 | 51 |
| Tmean _Aug | 16.6 | 16.3 | 14.6 | 11.392 | 0.213 | 0.280 | 0.705 | 1 | 50 | 5.255 | 0.001 | 0.004 | 0.734 | 1 | 51 |
| Tmean _Sep | 12.8 | 12.6 | 11.0 | 12.069 | 0.376 | 0.417 | 0.850 | 1 | 46 | 3.936 | 0.015 | 0.029 | 0.990 | 1 | 47 |
| Tmean _Oct | 8.3 | 8.4 | 6.5 | 11.946 | 0.297 | 0.271 | 0.837 | 1 | 47 | **0** | 0.011 | 0.005 | 0.999 | 1 | 47 |
| Tmean _Nov | 3.4 | 3.4 | 1.1 | 11.756 | 0.343 | 0.348 | 0.906 | 1 | 47 | 1.638 | 0.033 | 0.036 | 0.999 | 1 | 47 |
| Tmean _Dec | 1.0 | 0.9 | -1.3 | 12.530 | 0.226 | 0.240 | 0.837 | 1 | 47 | 1.960 | 0.002 | 0.002 | 0.973 | 1 | 49 |
| Tmean _Year | 8.0 | 7.9 | 5.9 | 11.234 | 0.338 | 0.378 | 0.864 | 1 | 47 | 29.623 | 0.010 | 0.018 | 0.985 | 1 | 49 |
|  |  |  |  |  |  |  |  |  |  |  |  |  |  |  |  |
| Tmax_Jan | 6.2 | 6.8 | 3.9 | n.s. | 0.469 | 0.382 | 0.776 | 1 | 43 | n.s. | 5.0*10^-58^ | 4.2*10^-107^ | 1.000 | 1 | 47 |
| Tmax _Feb | 9.5 | 10.4 | 7.3 | 13.612 | 0.219 | 0.116 | 0.641 | 1 | 51 | 6.282 | 5.1*10^-6^ | 2.0*10^-8^ | 0.802 | 1 | 52 |
| Tmax _Mar | 11.9 | 12.4 | 9.6 | 15.199 | 0.608 | 0.524 | 0.881 | 3 | 34 | 1.263 | 0.132 | 0.012 | 0.999 | 1 | 45 |
| Tmax _Apr | 15.5 | 16.9 | 14.3 | 17.863 | 0.692 | 0.473 | 0.831 | 2 | 26 | 2.479 | 0.578 | 0.004 | 0.995 | 2 | 52 |
| Tmax _May | 19.9 | 21.3 | 18.9 | 18.031 | 0.700 | 0.484 | 0.817 | 2 | 38 | 5.852 | 0.023 | 1.0*10^-4^ | 0.540 | 1 | 28 |
| Tmax _Jun | 21.4 | 23.0 | 20.0 | 15.515 | 0.891 | 0.745 | 0.953 | 3 | 0 | 6.574 | 0.991 | 0.382 | 0.999 | 2 | 52 |
| Tmax _Jul | 27.4 | 28.5 | 26.3 | n.s. | 0.256 | 0.159 | 0.384 | 0 | 52 | 5.170 | 3.6*10^-4^ | 4.6*10^-6^ | 0.027 | 0 | 51 |
| Tmax _Aug | 25.4 | 26.6 | 24.0 | 16.424 | 0.640 | 0.429 | 0.830 | 2 | 42 | n.s. | 1.4*10^-3^ | 1.1*10^-6^ | 0.863 | 1 | 49 |
| Tmax _Sep | 21.9 | 23.9 | 20.9 | 14.884 | 0.769 | 0.408 | 0.880 | 2 | 34 | 5.709 | 0.245 | 7.6*10^-5^ | 0.955 | 1 | 52 |
| Tmax _Oct | 15.5 | 16.9 | 14.2 | n.s. | 0.419 | 0.368 | 0.468 | 0 | 52 | n.s. | 3.2*10^-136^ | 0.000 | 1.000 | 1 | 48 |
| Tmax _Nov | 9.9 | 10.3 | 8.5 | 18.265 | 0.372 | 0.298 | 0.654 | 1 | 50 | n.s. | 7.0*10^-33^ | 4.9*10^-65^ | 1.000 | 1 | 47 |
| Tmax _Dec | 5.9 | 6.8 | 4.5 | 13.932 | 0.770 | 0.559 | 0.938 | 3 | 13 | n.s. | 0.327 | 0.002 | 0.999 | 1 | 47 |
|  |  |  |  |  |  |  |  |  |  |  |  |  |  |  |  |
| Tmin_Jan | -9.4 | -11.5 | -11.5 | n.s. | 0.194 | 0.248 | 0.248 | 0 | 52 | 23.025 | 0.001 | 0.067 | 0.067 | 0 | 52 |
| Tmin _Feb | -10.6 | -13.0 | -12.8 | n.s. | 0.100 | 0.254 | 0.237 | 0 | 52 | 32.063 | 0.001 | 0.017 | 0.014 | 0 | 52 |
| Tmin _Mar | -5.0 | -8.1 | -7.2 | 2.966 | 0.553 | 0.987 | 0.958 | 3 | 36 | n.s. | 6.4*10^-5^ | 0.999 | 0.999 | 2 | 45 |
| Tmin _Apr | 0.8 | -0.9 | -0.9 | 3.616 | 0.051 | 0.407 | 0.407 | 0 | 51 | 5.343 | 9.2*10^-5^ | 0.391 | 0.391 | 0 | 51 |
| Tmin _May | 3.9 | 3.0 | 1.9 | 12.203 | 0.166 | 0.373 | 0.694 | 1 | 50 | 8.583 | 1.9*10^-4^ | 0.040 | 0.967 | 1 | 50 |
| Tmin _Jun | 7.8 | 7.0 | 6.1 | 6.881 | 0.064 | 0.196 | 0.507 | 1 | 51 | 14.656 | 0.003 | 0.077 | 0.754 | 1 | 49 |
| Tmin _Jul | 9.6 | 8.7 | 8.2 | 4.586 | 0.133 | 0.437 | 0.656 | 1 | 50 | 5.203 | 0.001 | 0.112 | 0.629 | 1 | 51 |
| Tmin _Aug | 9.3 | 9.1 | 8.4 | n.s. | 0.289 | 0.314 | 0.408 | 0 | 52 | 16.200 | 0.007 | 0.014 | 0.168 | 0 | 52 |
| Tmin _Sep | 6.2 | 5.0 | 4.7 | 4.933 | 0.029 | 0.205 | 0.307 | 0 | 52 | 28.725 | 0.041 | 0.181 | 0.250 | 0 | 52 |
| Tmin _Oct | 2.5 | 2.1 | 1.2 | 8.528 | 0.053 | 0.094 | 0.290 | 0 | 52 | 24.154 | 0.006 | 0.015 | 0.105 | 0 | 52 |
| Tmin _Nov | -3.1 | -4.0 | -5.1 | 11.203 | 0.164 | 0.385 | 0.719 | 1 | 47 | 27.305 | 0.218 | 0.409 | 0.678 | 1 | 47 |
| Tmin _Dec | -5.6 | -7.0 | -7.4 | **0** | 0.016 | 0.195 | 0.343 | 0 | 52 | 17.260 | 8.0*10^-4^ | 0.012 | 0.027 | 0 | 52 |
|  |  |  |  |  |  |  |  |  |  |  |  |  |  |  |  |
| Tmax - Tmin_Jan | 15.6 | 18.3 | 15.4 | n.s. | 0.468 | 0.412 | 0.472 | 0 | 51 | 22.369 | 0.935 | 0.045 | 0.960 | 2 | 4 |
| Tmax - Tmin_Feb | 20.1 | 23.4 | 20.1 | n.s. | 0.523 | 0.301 | 0.523 | 2 | 17 | 28.329 | 0.915 | 0.077 | 0.915 | 2 | 5 |
| Tmax - Tmin_Mar | 16.9 | 20.5 | 16.8 | 17.725 | 0.165 | 0.883 | 0.151 | 1 | 47 | n.s. | 0.111 | 0.247 | 0.109 | 0 | 52 |
| Tmax - Tmin_Apr | 14.7 | 17.8 | 15.2 | 12.040 | 0.001 | 0.152 | 0.002 | 0 | 52 | n.s. | 0.854 | 0.011 | 0.680 | 2 | 36 |
| Tmax - Tmin_May | 16.0 | 18.3 | 17.0 | n.s. | 0.164 | 0.289 | 0.212 | 0 | 52 | 24.873 | 0.412 | 0.001 | 0.041 | 0 | 50 |
| Tmax - Tmin_Jun | 13.6 | 16.0 | 13.9 | n.s. | 0.494 | 0.430 | 0.486 | 0 | 52 | 23.531 | 0.986 | 0.316 | 0.974 | 2 | 6 |
| Tmax - Tmin_Jul | 17.8 | 19.8 | 18.1 | n.s. | 0.357 | 0.655 | 0.400 | 1 | 40 | n.s. | 0.067 | 0.007 | 0.048 | 0 | 52 |
| Tmax - Tmin_Aug | 16.1 | 17.5 | 15.6 | n.s. | 0.346 | 0.346 | 0.346 | 0 | 52 | 12.962 | 0.015 | 9.2*10^-7^ | 0.335 | 0 | 51 |
| Tmax - Tmin_Sep | 15.7 | 18.9 | 16.2 | n.s. | 0.262 | 0.329 | 0.272 | 0 | 52 | 26.950 | 0.573 | 0.002 | 0.321 | 1 | 52 |
| Tmax - Tmin_Oct | 13.0 | 14.8 | 13.0 | 9.137 | 0.001 | 0.027 | 0.001 | 0 | 52 | 25.245 | 0.475 | 0.010 | 0.475 | 0 | 35 |
| Tmax - Tmin_Nov | 13.0 | 14.3 | 13.6 | 16.559 | 0.099 | 0.481 | 0.227 | 0 | 48 | n.s. | 0.117 | 0.089 | 0.103 | 0 | 52 |
| Tmax - Tmin_Dec | 11.5 | 13.8 | 11.9 | n.s. | 0.029 | 0.165 | 0.040 | 0 | 52 | n.s. | 0.785 | 0.251 | 0.706 | 2 | 6 |

**Table S10**. Climatic conditions in QTK25s of the entire study area as well as in QTK25s with presences and absences of *T. helenitis* for the periods 1900–1949 and 1950–1979. m_1900_49,_ m_1950_79_: median value of the variable in the entire area for 1900–1949 and 1950–1979, respectively. Δm: m_1950_79_ - m_1900_49_; m_presence_1900_49_, m_presence_1950_79,_ m_absence_1900_49,_ m_absence_1950_79_: medians of presences and absences of the respective period; W, p: results of Wilcoxon tests of differences between presences (absences) between periods; significance levels: red p > 0.05, * p ≤ 0.05, ** p ≤ 0.01, *** p ≤ 0.001. For a graphical representation see Figure S3.

|  | m_1900_49_ | m_1950_79_ | Δm | m_presence_1900_49_ | m_presence_1950_79_ | W | p | m_absence_1900_49_ | m_absence_1950_79_ | W | p |
| --- | --- | --- | --- | --- | --- | --- | --- | --- | --- | --- | --- |
| Precip_Jan | 61.86 | 60.55 | -1.31*** | 62.40 | 83.74 | 35 | 0.073 | 63.53 | 59.37 | 1025 | 0.148 |
| Precip_Feb | 51.55 | 53.75 | 2.20*** | 50.41 | 73.18 | 24 | 0.013 | 51.82 | 53.29 | 834 | 0.791 |
| Precip_Mar | 48.29 | 50.38 | 2.09*** | 52.63 | 68.22 | 19 | 0.004 | 47.54 | 49.98 | 765 | 0.375 |
| Precip_Apr | 55.02 | 55.16 | 0.14 | 58.13 | 72.23 | 27 | 0.022 | 54.68 | 53.96 | 921 | 0.611 |
| Precip_May | 62.33 | 67.70 | 5.37*** | 63.55 | 78.66 | 18 | 0.004 | 61.52 | 66.47 | 584 | 0.011 |
| Precip_Jun | 69.19 | 77.68 | 8.49*** | 71.81 | 97.25 | 8 | 2.0*10^-4^ | 69.13 | 76.94 | 528 | 0.002 |
| Precip_Jul | 76.77 | 76.64 | -0.13 | 77.51 | 89.03 | 44 | 0.209 | 76.82 | 75.22 | 904 | 0.723 |
| Precip_Aug | 77.00 | 82.66 | 5.66*** | 79.69 | 95.82 | 19 | 0.004 | 76.57 | 81.84 | 675 | 0.088 |
| Precip_Sep | 58.46 | 58.51 | 0.05 | 61.10 | 73.79 | 23 | 0.010 | 57.58 | 57.27 | 919 | 0.624 |
| Precip_Oct | 63.59 | 55.54 | -8.05*** | 67.92 | 72.92 | 41 | 0.152 | 63.91 | 54.69 | 1286 | 9.6*10^-5^ |
| Precip_Nov | 61.71 | 73.38 | 11.67*** | 66.56 | 89.34 | 23 | 0.010 | 61.93 | 73.10 | 541 | 0.004 |
| Precip_Dec | 62.93 | 75.83 | 12.90*** | 65.51 | 108.79 | 14 | 0.001 | 64.30 | 75.31 | 524 | 0.002 |
| Precip_Year | 749.90 | 782.20 | 32.30*** | 804.00 | 980.50 | 22 | 0.008 | 749.90 | 776.30 | 771 | 0.405 |
|  |  |  |  |  |  |  |  |  |  |  |  |
| Tmean_Jan | -0.40 | -0.24 | 0.16 | -0.56 | -2.30 | 106 | 0.022 | -0.19 | -0.17 | 853 | 0.925 |
| Tmean_Feb | 0.60 | 0.65 | 0.05 | 0.21 | -1.49 | 103 | 0.035 | 0.69 | 0.70 | 863 | 0.996 |
| Tmean_Mar | 4.01 | 3.89 | -0.12*** | 3.49 | 1.56 | 110 | 0.010 | 4.05 | 3.90 | 1001 | 0.217 |
| Tmean_Apr | 7.80 | 7.74 | -0.06 | 7.40 | 5.48 | 105 | 0.025 | 7.99 | 7.76 | 983 | 0.286 |
| Tmean_May | 12.39 | 12.10 | -0.29*** | 12.18 | 10.06 | 119 | 0.001 | 12.72 | 12.13 | 1210 | 0.002 |
| Tmean_Jun | 15.30 | 15.46 | 0.16*** | 14.92 | 13.39 | 97 | 0.083 | 15.54 | 15.53 | 788 | 0.497 |
| Tmean_Jul | 17.09 | 16.80 | -0.29** | 16.72 | 14.91 | 101 | 0.048 | 17.25 | 16.95 | 1007 | 0.199 |
| Tmean_Aug | 16.32 | 16.15 | -0.17 | 16.01 | 14.35 | 98 | 0.073 | 16.41 | 16.27 | 968 | 0.351 |
| Tmean_Sep | 13.03 | 13.14 | 0.11 | 12.86 | 11.40 | 102 | 0.041 | 13.23 | 13.18 | 910 | 0.683 |
| Tmean_Oct | 8.43 | 8.73 | 0.30*** | 8.17 | 7.17 | 94 | 0.120 | 8.47 | 8.74 | 523 | 0.002 |
| Tmean_Nov | 3.70 | 4.10 | 0.40*** | 3.34 | 2.03 | 98 | 0.073 | 3.75 | 4.11 | 483 | 4.6*10^-4^ |
| Tmean_Dec | 0.82 | 0.93 | 0.11*** | 0.52 | -1.02 | 102 | 0.041 | 0.84 | 1.01 | 705 | 0.153 |
| Tmean_Year | 8.29 | 8.30 | 0.01 | 7.97 | 6.30 | 102 | 0.041 | 8.39 | 8.31 | 896 | 0.777 |
|  |  |  |  |  |  |  |  |  |  |  |  |
| Tmax_Jan | 7.40 | 7.40 | 0.00 | 7.00 | 4.90 | 125 | 0.001 | 7.35 | 7.40 | 764.5 | 0.370 |
| Tmax_Feb | 9.10 | 9.00 | -0.10 | 8.50 | 7.30 | 97.5 | 0.077 | 9.20 | 9.00 | 1044.5 | 0.103 |
| Tmax_Mar | 13.90 | 12.90 | -1.00*** | 13.10 | 10.70 | 121 | 0.002 | 14.00 | 13.05 | 1388 | 2.2*10^-6^ |
| Tmax_Apr | 17.90 | 17.20 | -0.70*** | 17.60 | 15.00 | 128 | 4.1*10^-4^ | 18.10 | 17.25 | 1290.5 | 1.2*10^-4^ |
| Tmax_May | 22.30 | 20.50 | -1.80*** | 22.00 | 18.60 | 133 | 1.3*10^-4^ | 22.50 | 20.60 | 1614 | 1.2*10^-11^ |
| Tmax_Jun | 25.70 | 25.00 | -0.70*** | 25.50 | 22.30 | 132 | 1.7*10^-4^ | 25.90 | 25.05 | 1251 | 4.7*10^-4^ |
| Tmax_Jul | 26.80 | 27.00 | 0.20*** | 26.40 | 24.80 | 110.5 | 0.012 | 26.90 | 27.00 | 691.5 | 0.120 |
| Tmax_Aug | 27.30 | 26.00 | -1.30*** | 26.90 | 23.40 | 132 | 1.7*10^-4^ | 27.50 | 26.20 | 1552.5 | 4.8*10^-10^ |
| Tmax_Sep | 24.50 | 23.00 | -1.50*** | 24.10 | 21.20 | 129 | 3.3*10^-4^ | 24.70 | 23.00 | 1562 | 2.8*10^-10^ |
| Tmax_Oct | 17.80 | 15.90 | -1.90*** | 17.80 | 14.00 | 133 | 1.3*10^-4^ | 17.6 | 15.90 | 1640 | 2.3*10^-12^ |
| Tmax_Nov | 10.30 | 10.80 | 0.50*** | 10.20 | 9.00 | 97 | 0.082 | 10.30 | 10.80 | 367.5 | 7.1*10^-6^ |
| Tmax_Dec | 7.90 | 7.60 | -0.30*** | 7.60 | 5.30 | 125.5 | 7.1*10^-4^ | 7.90 | 7.70 | 1079.5 | 0.051 |
|  |  |  |  |  |  |  |  |  |  |  |  |
| Tmin_Jan | -14.70 | -11.20 | 3.50*** | -14.60 | -12.40 | 2 | 2.1*10^-4^ | -14.70 | -11.20 | 24 | 3.1*10^-14^ |
| Tmin_Feb | -14.00 | -14.60 | -0.60*** | -14.10 | -15.40 | 107.5 | 0.019 | -14.00 | -14.50 | 1386.5 | 2.3*10^-6^ |
| Tmin_Mar | -3.80 | -4.40 | -0.60*** | -5.20 | -6.90 | 117 | 0.004 | -3.65 | -4.30 | 1310.5 | 5.4*10^-5^ |
| Tmin_Apr | -0.30 | 0.40 | 0.70*** | -1.40 | -1.40 | 66 | 1.000 | -0.10 | 0.50 | 439.5 | 1.2*10^-4^ |
| Tmin_May | 3.50 | 3.60 | 0.10*** | 2.90 | 2.10 | 85 | 0.296 | 3.50 | 3.60 | 714 | 0.176 |
| Tmin_Jun | 6.80 | 7.80 | 1.00*** | 6.10 | 6.10 | 51 | 0.383 | 7.00 | 7.90 | 362 | 5.7*10^-6^ |
| Tmin_Jul | 9.20 | 9.70 | 0.50*** | 8.50 | 7.80 | 85.5 | 0.284 | 9.30 | 9.70 | 598 | 0.016 |
| Tmin_Aug | 9.20 | 9.20 | 0.00 | 8.50 | 8.00 | 82.5 | 0.370 | 9.30 | 9.20 | 955 | 0.413 |
| Tmin_Sep | 4.90 | 6.00 | 1.10*** | 4.10 | 4.80 | 32.5 | 0.053 | 5.05 | 6.00 | 337 | 1.9*10^-6^ |
| Tmin_Oct | 1.40 | 1.70 | 0.30*** | 0.70 | 0.50 | 62 | 0.817 | 1.50 | 1.75 | 664 | 0.071 |
| Tmin_Nov | -3.60 | -1.70 | 1.90*** | -4.10 | -3.60 | 41.5 | 0.156 | -3.50 | -1.65 | 78.5 | 1.2*10^-12^ |
| Tmin_Dec | -7.00 | -7.00 | 0.00 | -8.10 | -9.30 | 118 | 0.003 | -6.80 | -6.90 | 1021.5 | 0.155 |
|  |  |  |  |  |  |  |  |  |  |  |  |
| Tmax - Tmin_Jan | 21.50 | 18.40 | -3.10*** | 21.40 | 17.20 | 133 | 1.4*10^-4^ | 21.75 | 18.45 | 1695 | 6.0*10^-14^ |
| Tmax - Tmin_Feb | 22.90 | 23.30 | 0.40*** | 22.50 | 22.50 | 68.5 | 0.931 | 23.00 | 23.40 | 548.5 | 0.004 |
| Tmax - Tmin_Mar | 17.70 | 17.40 | -0.30*** | 18.10 | 17.40 | 97.5 | 0.077 | 17.65 | 17.45 | 1098 | 0.034 |
| Tmax - Tmin_Apr | 18.20 | 16.70 | -1.50*** | 18.60 | 16.30 | 133 | 1.3*10^-4^ | 18.10 | 16.70 | 1572.5 | 1.5*10^-10^ |
| Tmax - Tmin_May | 19.10 | 16.90 | -2.20*** | 19.30 | 16.20 | 133 | 1.3*10^-4^ | 19.05 | 17.00 | 1685.5 | 1.1*10^-13^ |
| Tmax - Tmin_Jun | 18.90 | 17.30 | -1.60*** | 19.20 | 15.90 | 133 | 1.3*10^-4^ | 18.75 | 17.30 | 1569.5 | 1.8*10^-10^ |
| Tmax - Tmin_Jul | 17.50 | 17.30 | -0.20*** | 17.70 | 16.90 | 119.5 | 0.002 | 17.45 | 17.40 | 938 | 0.506 |
| Tmax - Tmin_Aug | 18.10 | 16.70 | -1.40*** | 18.20 | 15.50 | 133 | 1.3*10^-4^ | 18.05 | 16.80 | 1471.5 | 4.0e*10^-8^ |
| Tmax - Tmin_Sep | 19.60 | 17.20 | -2.40*** | 19.40 | 16.50 | 133 | 1.3*10^-4^ | 19.60 | 17.20 | 1682 | 1.4*10^-13^ |
| Tmax - Tmin_Oct | 16.30 | 14.10 | -2.20*** | 17.00 | 13.20 | 133 | 1.3*10^-4^ | 16.10 | 14.15 | 1665 | 4.6*10^-13^ |
| Tmax - Tmin_Nov | 13.90 | 12.40 | -1.50*** | 14.10 | 12.60 | 133 | 1.3*10^-4^ | 13.80 | 12.35 | 1506 | 6.6*10^-9^ |
| Tmax - Tmin_Dec | 14.90 | 14.60 | -0.30*** | 15.00 | 14.50 | 104.5 | 0.030 | 14.80 | 14.60 | 964.5 | 0.365 |

**Table S11**. Results of Ansari-Bradley tests of variance homogeneity of climatic conditions in 1900–1949 and in 1950–1979 for presences. 1900–1949: N=19, 1950–1979: N=10. Red = non-significant p-value, blue = 0.1 ≥ p > 0.05. For graphical representation see Figure S3.

|  | AB | p |
| --- | --- | --- |
| Precip_Jan | 127.0 | 0.532 |
| Precip_Feb | 131.0 | 0.865 |
| Precip_Mar | 135.0 | 0.865 |
| Precip_Apr | 128.0 | 0.610 |
| Precip_May | 137.0 | 0.692 |
| Precip_Jun | 146.0 | 0.151 |
| Precip_Jul | 118.0 | 0.093 |
| Precip_Aug | 136.0 | 0.777 |
| Precip_Sep | 134.0 | 0.955 |
| Precip_Oct | 121.0 | 0.187 |
| Precip_Nov | 134.0 | 0.955 |
| Precip_Dec | 140.0 | 0.460 |
| Precip_Year | 133.0 | 1.000 |
|  |  |  |
| Tmean_Jan | 127.0 | 0.532 |
| Tmean_Feb | 125.0 | 0.393 |
| Tmean_Mar | 132.0 | 0.955 |
| Tmean_Apr | 127.0 | 0.532 |
| Tmean_May | 140.0 | 0.460 |
| Tmean_Jun | 123.0 | 0.278 |
| Tmean_Jul | 127.0 | 0.532 |
| Tmean_Aug | 124.0 | 0.333 |
| Tmean_Sep | 126.0 | 0.460 |
| Tmean_Oct | 116.0 | 0.054 |
| Tmean_Nov | 120.0 | 0.151 |
| Tmean_Dec | 124.0 | 0.333 |
| Tmean_Year | 124.0 | 0.333 |
|  |  |  |
| Tmax_Jan | 146.0 | 0.131 |
| Tmax_Feb | 121.5 | 0.179 |
| Tmax_Mar | 142.0 | 0.296 |
| Tmax_Apr | 149.0 | 0.063 |
| Tmax_May | 154.0 | 0.014 |
| Tmax_Jun | 153.0 | 0.019 |
| Tmax_Jul | 134.5 | 0.861 |
| Tmax_Aug | 153.0 | 0.020 |
| Tmax_Sep | 150.0 | 0.048 |
| Tmax_Oct | 154.0 | 0.014 |
| Tmax_Nov | 119.0 | 0.102 |
| Tmax_Dec | 146.5 | 0.116 |
|  |  |  |
| Tmin_Jan | 152.0 | 0.027 |
| Tmin_Feb | 128.5 | 0.601 |
| Tmin_Mar | 138.0 | 0.561 |
| Tmin_Apr | 115.0 | 0.043 |
| Tmin_May | 110.0 | 0.007 |
| Tmin_Jun | 116.0 | 0.052 |
| Tmin_Jul | 116.5 | 0.054 |
| Tmin_Aug | 113.5 | 0.023 |
| Tmin_Sep | 125.5 | 0.383 |
| Tmin_Oct | 119.0 | 0.103 |
| Tmin_Nov | 124.5 | 0.320 |
| Tmin_Dec | 142.0 | 0.293 |
|  |  |  |
| Tmax - Tmin_Jan | 154.0 | 0.015 |
| Tmax - Tmin_Feb | 117.5 | 0.081 |
| Tmax - Tmin_Mar | 132.5 | 0.953 |
| Tmax - Tmin_Apr | 154.0 | 0.015 |
| Tmax - Tmin_May | 155.0 | 0.013 |
| Tmax - Tmin_Jun | 154.0 | 0.014 |
| Tmax - Tmin_Jul | 140.5 | 0.382 |
| Tmax - Tmin_Aug | 154.0 | 0.014 |
| Tmax - Tmin_Sep | 155.0 | 0.014 |
| Tmax - Tmin_Oct | 154.0 | 0.015 |
| Tmax - Tmin_Nov | 154.0 | 0.015 |
| Tmax - Tmin_Dec | 126.5 | 0.465 |

**Figure S6**. Distribution of Tmin_Mar for 1900–1949, 1950–1979 and 2000–2020, and habitat suitability in 1900–1949 and 1950–1979 as predicted by logistic regression models in Hessia. Tmin_Mar refers to vernalization (see discussion). It is shown for all 892 QTK25s covering Hessia for three periods. Habitat suitability (occurrence probabilities, Prob.) for the periods 1900–1949 and 1950–1979 was calculated from the respective logit curve (Tables S5 and S7). White squares = absences and red squares = presences in the respective period. Boxplot: letters (a,b,c) give significances of matched-pairs Wilcoxon tests of median differences in Tmin_Mar between periods for Hessia. Dashed line = value for which the logit curve calculates an occurrence probability of 0.5 (threshold for period 1900–1949), dotted line = value for which the logit curve calculates an occurrence probability of 0.5 (threshold for period 1950–1979).


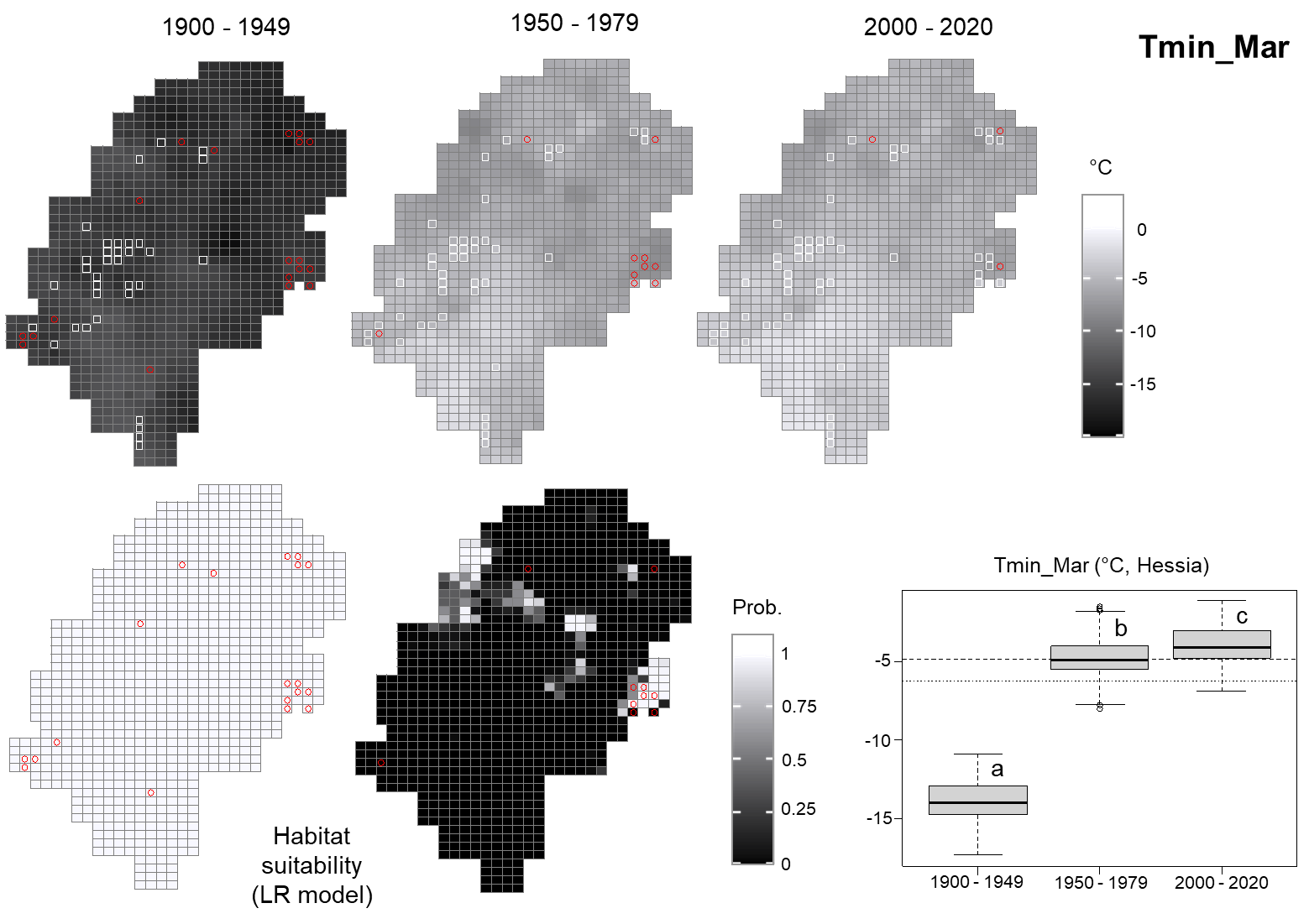


**Figure S7**. Distribution of Tmin_Apr for 1900–1949, 1950–1979 and 2000–2020, and habitat suitability in 1900–1949 and 1950–1979 as predicted by logistic regression models in Hessia. Tmin_Apr refers to vernalization (see discussion). It is shown for all 892 QTK25s covering Hessia for three periods. Habitat suitability (occurrence probabilities, Prob.) for the periods 1900–1949 and 1950–1979 was calculated from the respective logit curve (Tables S5 and S7). White squares = absences and red squares = presences in the respective period. Boxplot: letters (a,b,c) give significances of matched-pairs Wilcoxon tests of median differences in Tmin_Apr between periods for Hessia. Dashed line = value for which the logit curve calculates an occurrence probability of 0.5 (threshold for period 1900–1949), dotted line = value for which the logit curve calculates an occurrence probability of 0.5 (threshold for period 1950–1979).


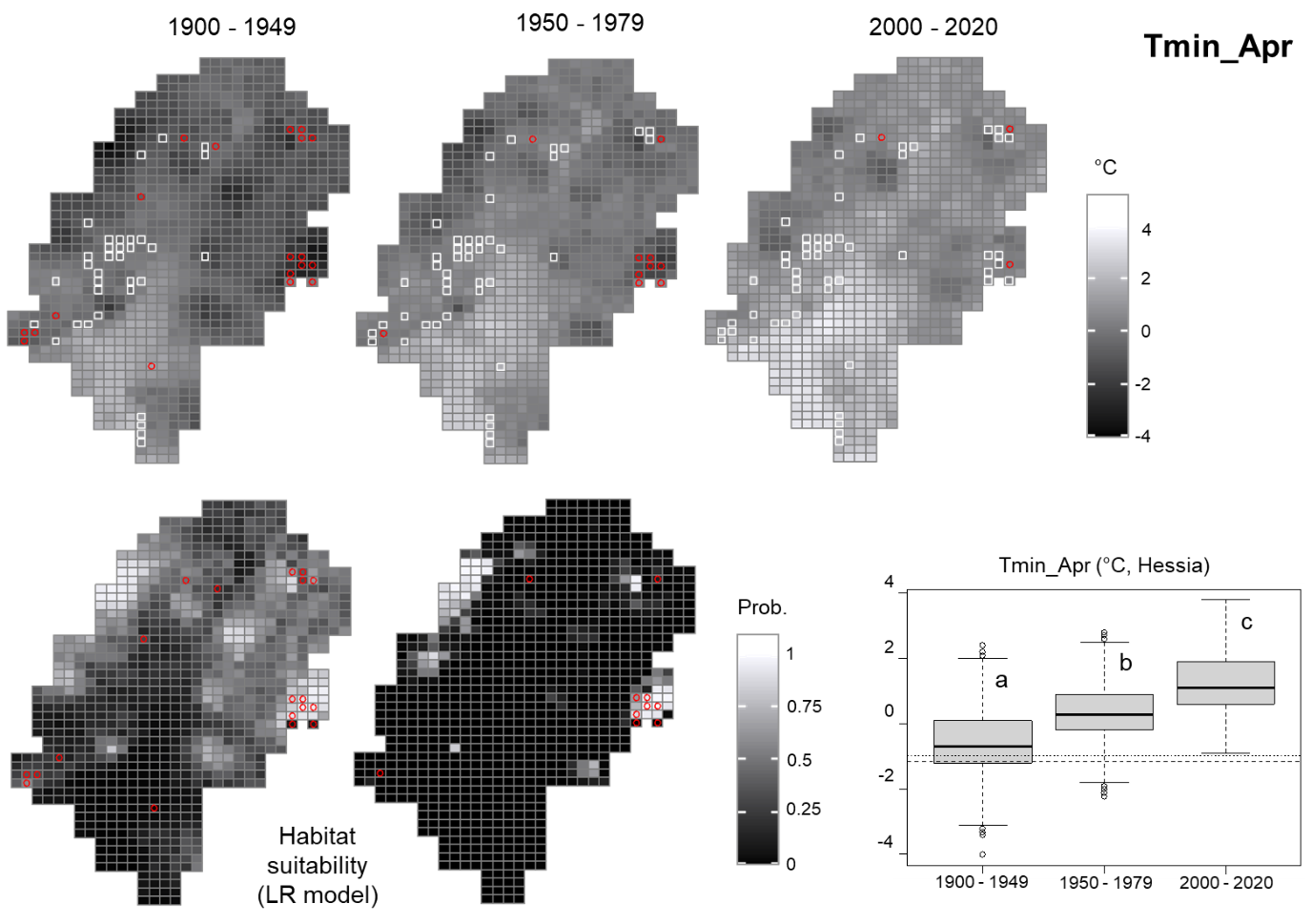


**Figure S8**. Distribution of Tmin_Jul for 1900–1949, 1950–1979 and 2000–2020, and habitat suitability in 1900–1949 and 1950–1979 as predicted by logistic regression models in Hessia. Tmin_Jul refers to seed germination (see discussion). It is shown for all 892 QTK25s covering Hessia for three periods, 1900–1949, 1950–1979 and 2000–2020. Habitat suitability (occurrence probabilities, Prob.) for the periods 1900–1949 and 1950–1979 were calculated from the respective logit curves (Tables S5 and S7). White squares = absences and red squares = presences in the respective period. Boxplot: letters (a,b,c) give significances of matched-pairs Wilcoxon tests of median differences in Tmin_Jul between periods for Hessia. Dashed line = value for which the logit curve calculates an occurrence probability of 0.5 (threshold for period 1900–1949), dotted line = value for which the logit curve calculates an occurrence probability of 0.5 (threshold for period 1950–1979).


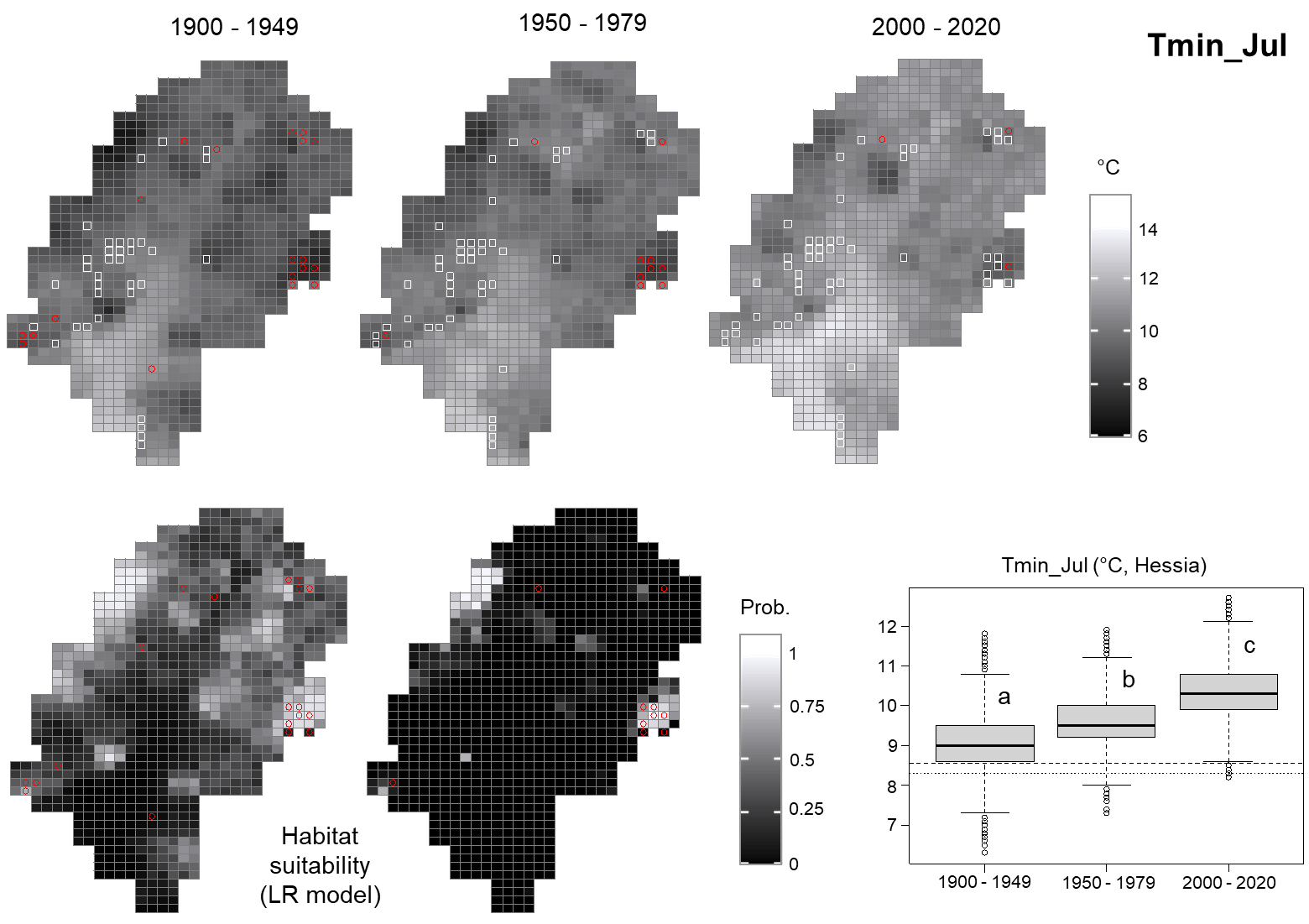


**Figure S9**. Habitat suitability for the period 2000–2020 in Germany (total area) and Hessia (yellow outline and bottom right of each partial figure). A) Tmin_Mar, B) Tmin_Apr (vernalization, see discussion) and C) Tmin_Jul (seed germination, see discussion). Habitat suitability (occurrence probabilities, Prob.) was calculated from respective logit curves for 1900–1949 (Table S5) and 1950–1979 (Table S7). Red squares = the three presences in 2000–2020.

A)


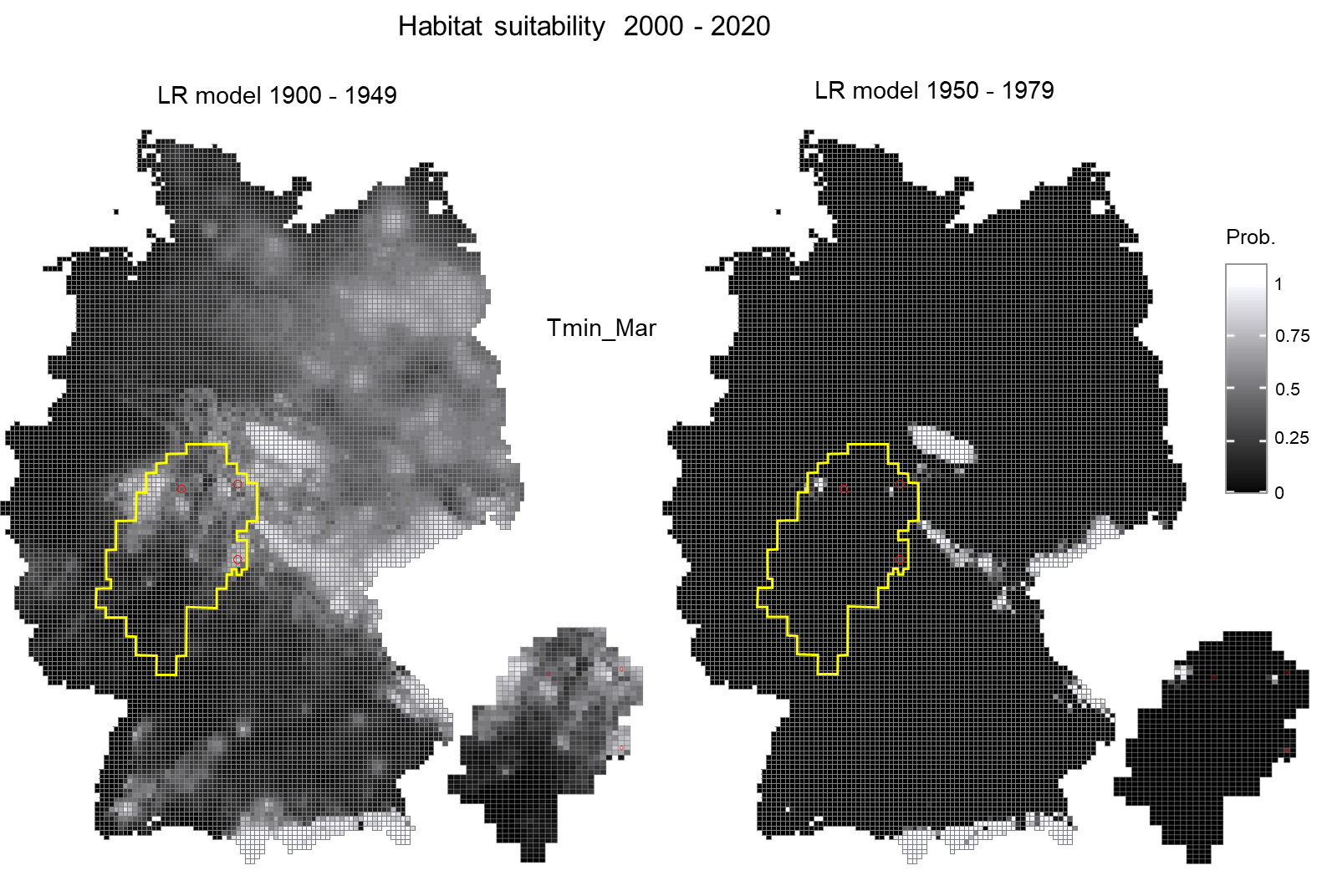


B)


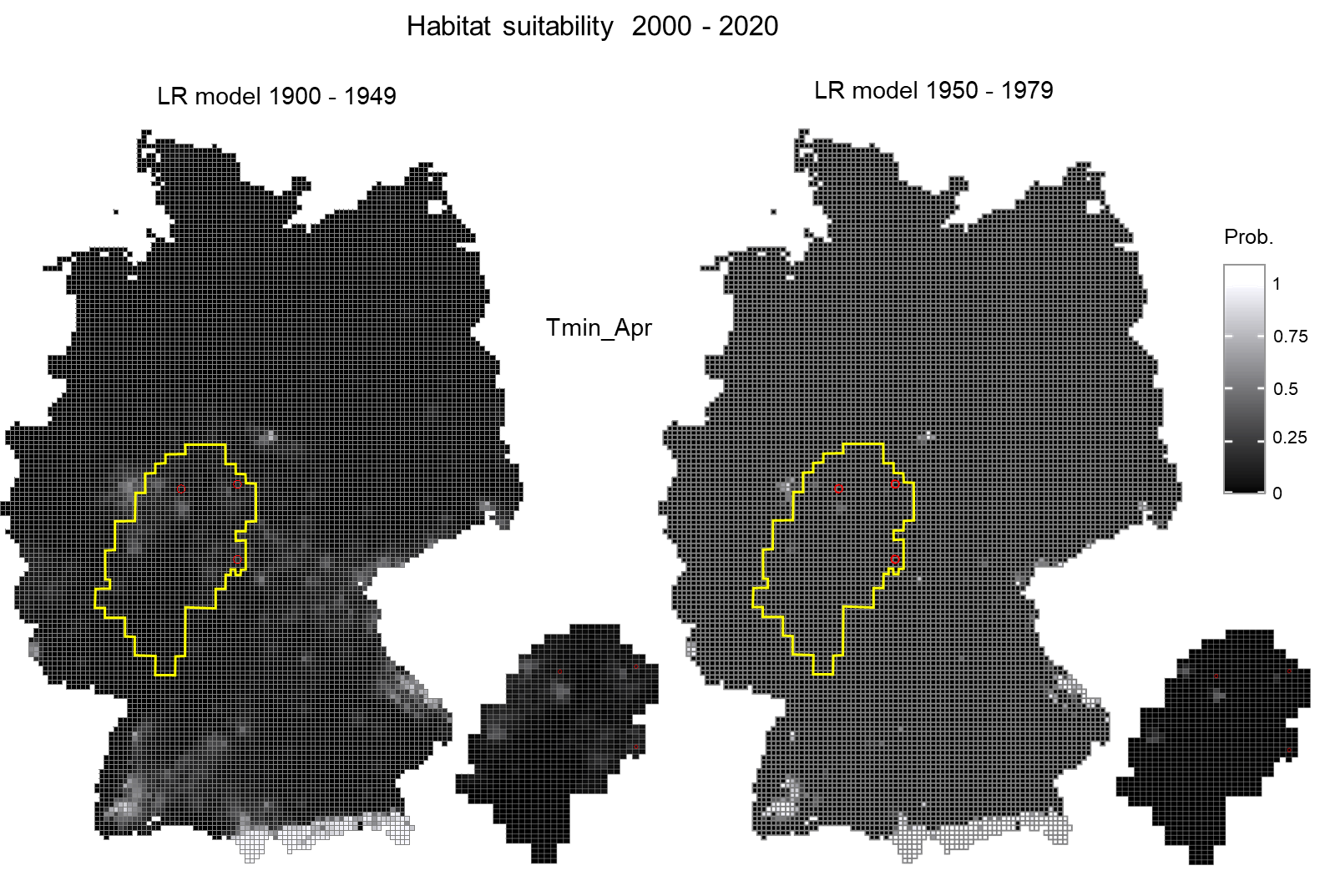


C)


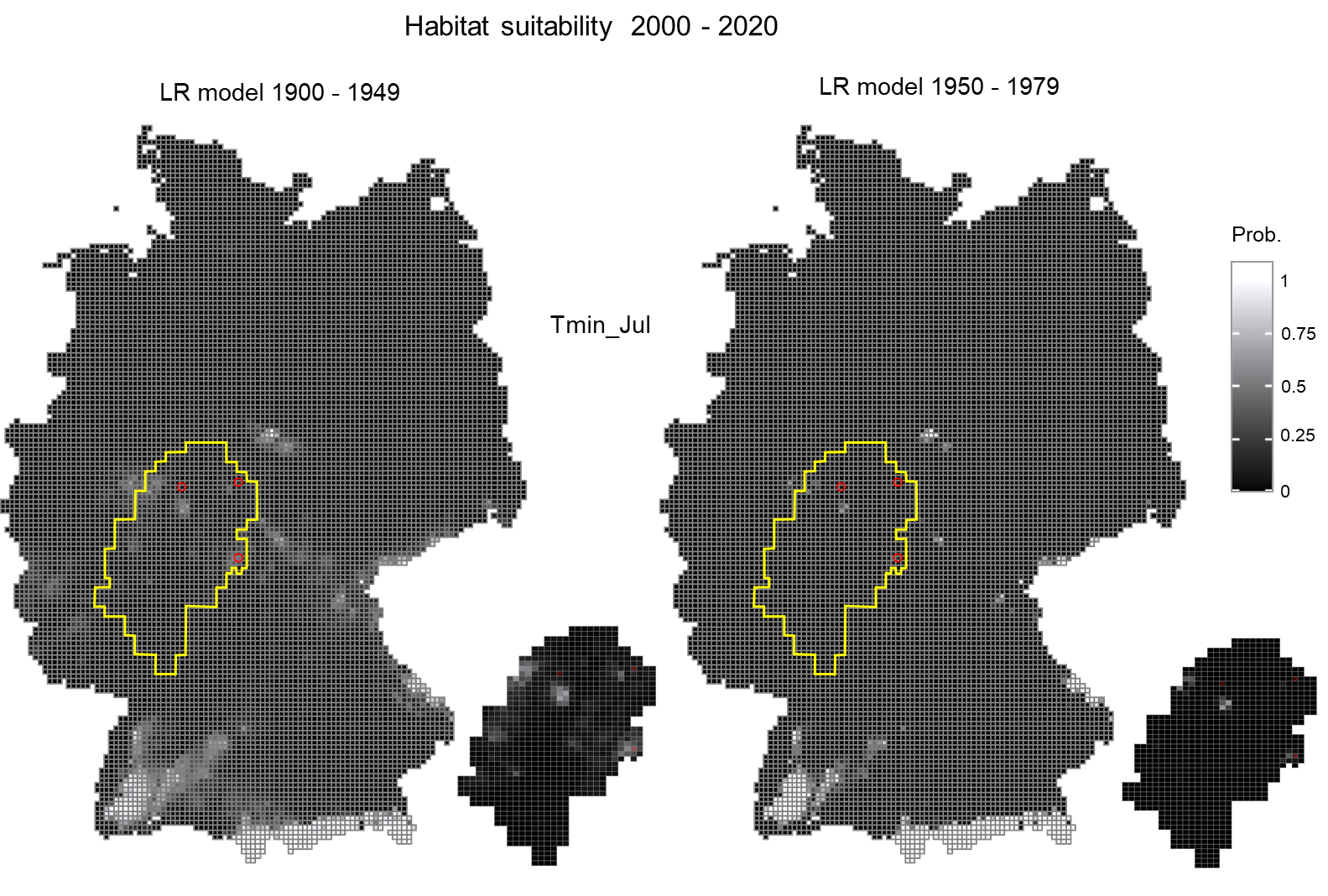

Supplement: Supplementary file 1 — Appendix S1 [file ECE3-13-e10769-s002.docx]
